# Supplementary material for: CAR-T triggers TAM reeducation and adaptive anti-tumor response via TREM2 deficiency or CD40 agonist
Source: Cell Rep Med. 2026 Jan 20;7(1):102539. doi: 10.1016/j.xcrm.2025.102539 (PMC12866111; doi:10.1016/j.xcrm.2025.102539)

Cell Reports Medicine, Volume *7*

Supplemental information

CAR-T triggers TAM reeducation and adaptive anti-tumor response via *TREM2* deficiency or CD40 agonist

Ting Liu, Huixin Gao, Zhihui Xi, TianTian Yu, Yimei Gu, Hanbing Mai, Hui Yuan, Yafang Liu, Haikuan Liu, Qiaoxuan Zhang, Xianzhang Huang, Wenzhe Fan, and Jizhou Tan

Supplemental information

Supplemental Figures and figure legends

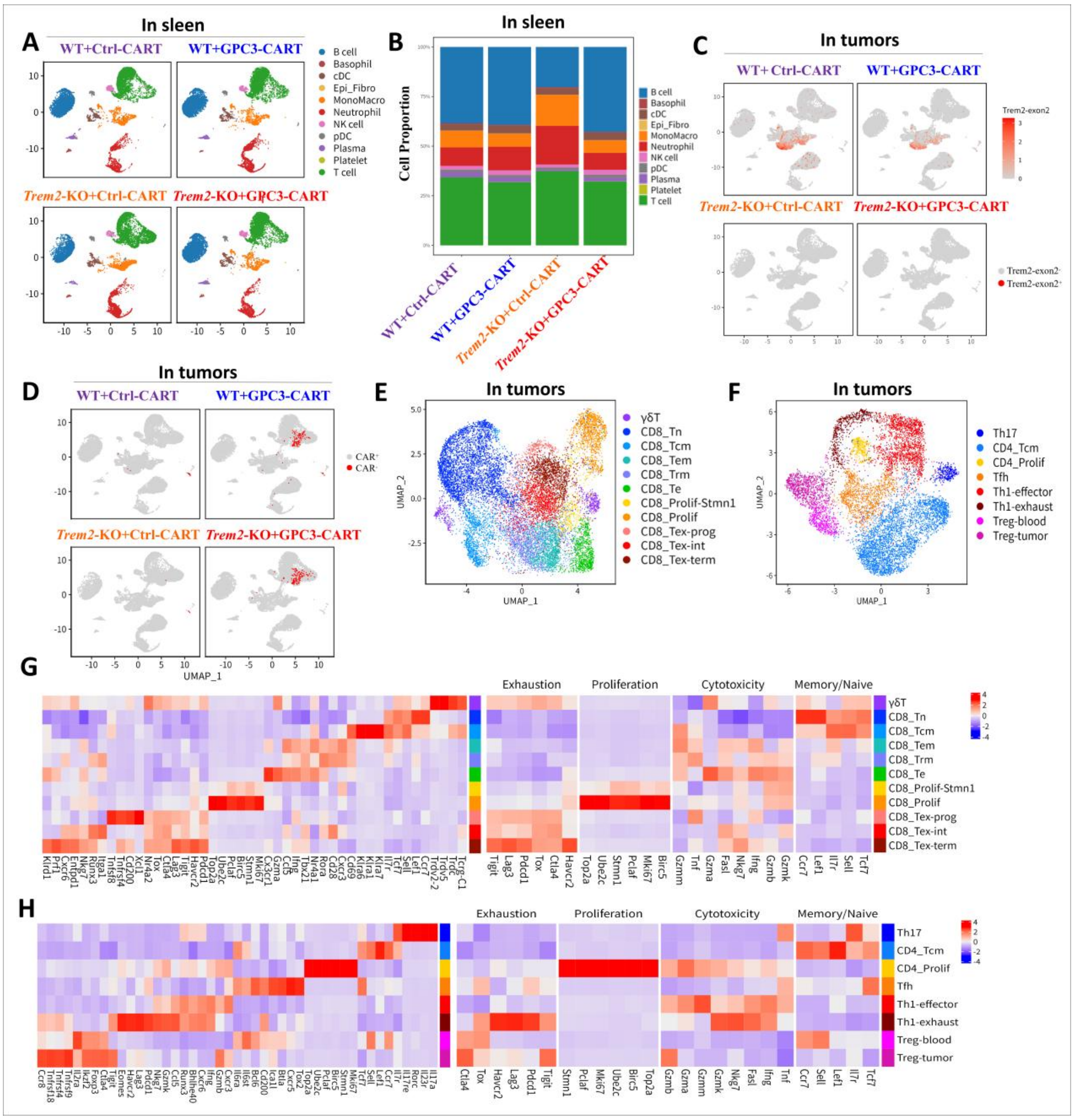

Figure S1. Cell clusters and corresponding markers in CD8⁺ and CD4⁺ T cells, related to Figure 1 and Figure 2.

(A) UMAP plots of individual group displayed distinct intrasplenic cell populations in spleen. N=2 for all treatment groups on scRNA sequence.

(B) Proportion of the distinct intrasplenic cell populations.

(C) Normalised expression of the indicated *Trem2*-exon2 in each tumor group.

(D) Normalised expression of the indicated CAR-T in each tumor group.

(E-F) Cell distribution in $CD8^+$ T (C) and $CD4^+$ T cells colored by distinct functional clusters.

(G) Heatmap of normalized expression level of top 4-6 cluster markers in each $CD8^+$ T cell clusters (left panel). Heatmaps express as Z score of gene collections on behalf of exhaustion, proliferation, cytotoxicity, and memory/naive phenotypes. $CD8^+$T subtypes are divided based on their function (right panel).

(H) Heatmap of normalized expression level of top 4-6 cluster markers in each $CD4^+$ T cell clusters (left panel). Heatmaps express as Z score of gene collections on behalf of exhaustion, proliferation, cytotoxicity, and memory/naive phenotypes from $CD4^+$T subtypes (right panel).

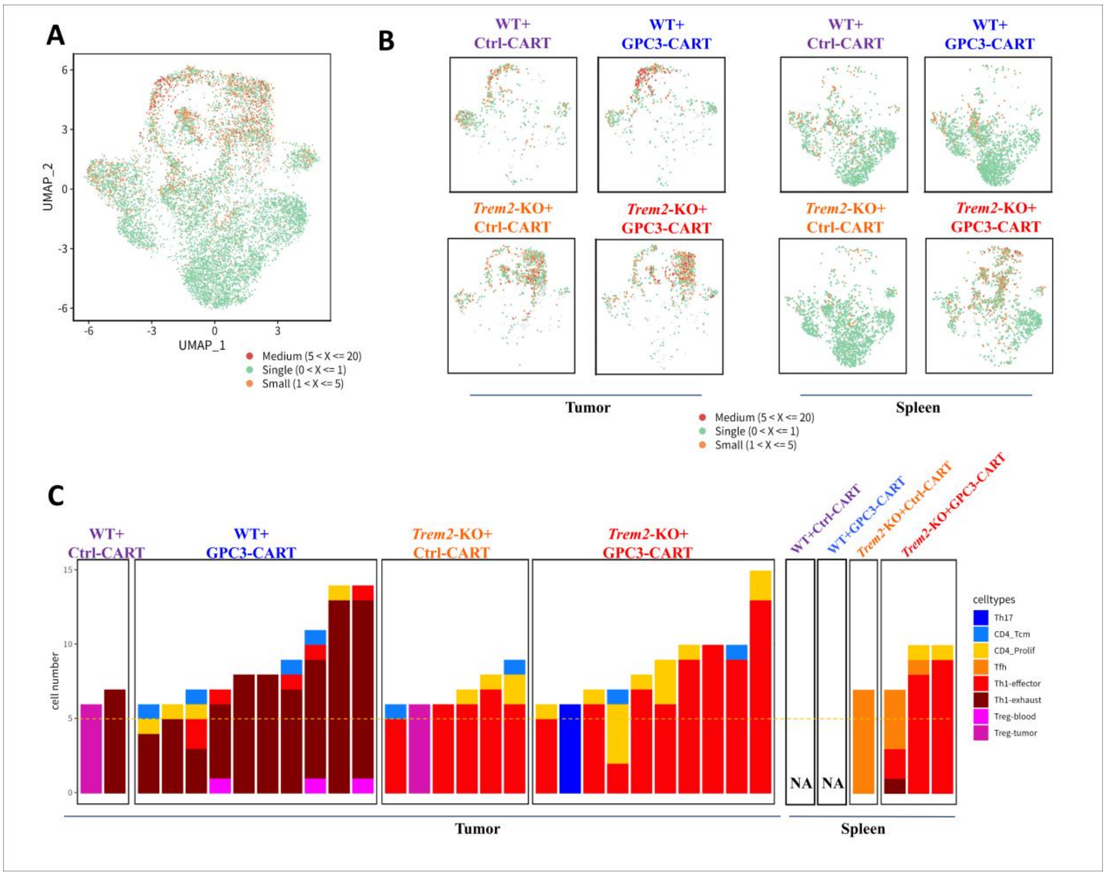

Figure S2. related to Figure 3.

(A) TCR expansion level of $CD4^+$ T cells in UMAP colored by different clonotype groups.

(B) Same to A. The plot was faceted by different groups.

(C) The bar chart revealed the number of TCR clones in each group. Each bar on the X-axis represents a TCR clone. The Y-axis represented the cell number of each TCR. The red dotted line represented the dividing line of large expansion TCR clone, and the orange dotted line represented the dividing line of

medium expansion TCR clone. Cell types were also colored.

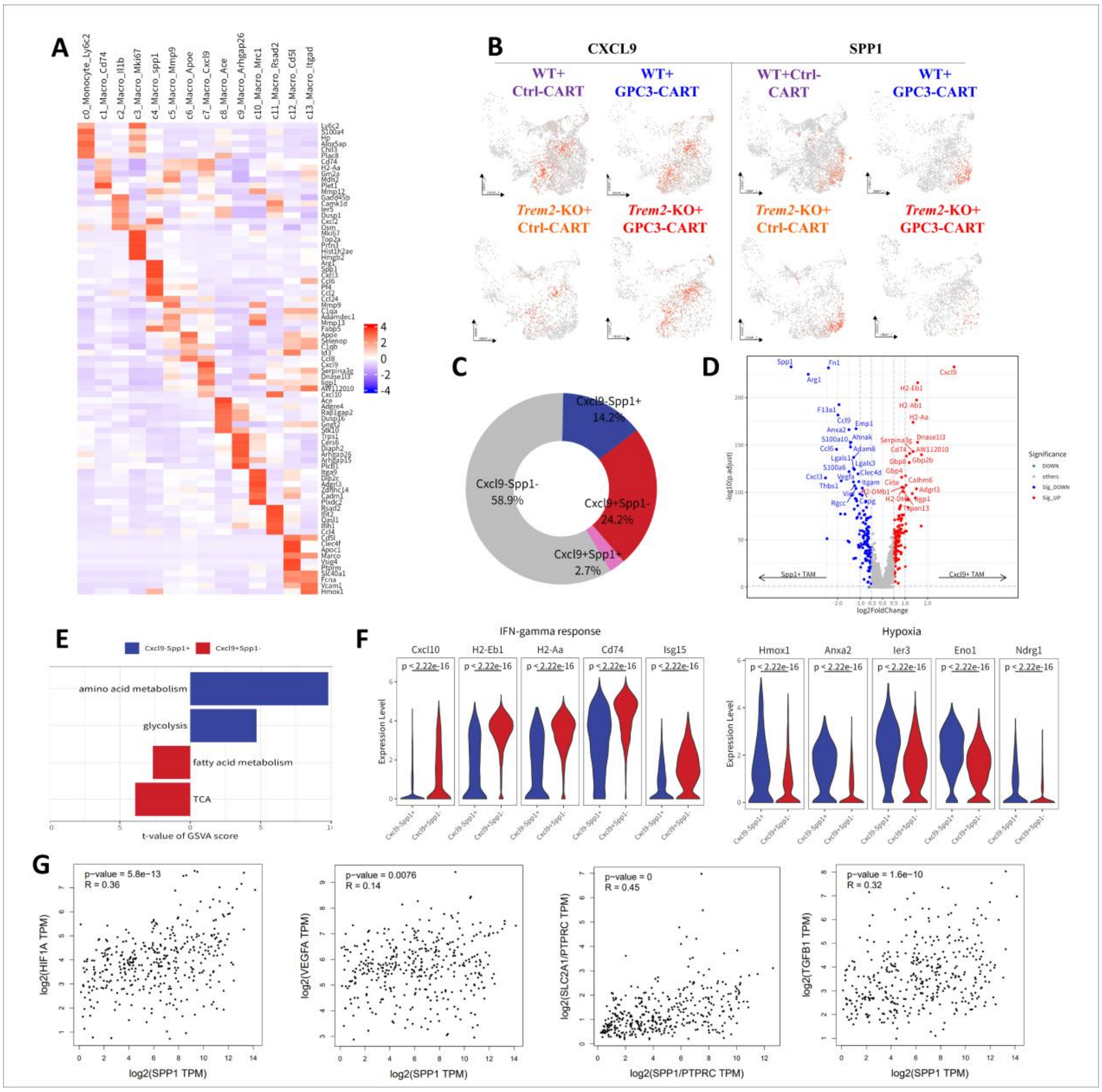

Figure S3. Different functions between *Cxcl9*$^{+}$ and *Spp1*$^{+}$ macrophages, related to Figure 4 and Figure 5.

(A) Heatmap of normalized expression level of top cluster markers in each monocyte or macrophage clusters.

(B) Normalised expression of *Cxcl9* and *Spp1* in TAMs among different groups.

(C) The cell proportion of *Cxcl9*$^{+}$*Spp1*$^{+}$, *Cxcl9*$^{+}$*Spp1*$^{-}$, *Cxcl9*$^{-}$*Spp1*$^{+}$ and *Cxcl9*$^{-}$*Spp1*$^{-}$ macrophages.

(D) Volcano plot shows the different expressed genes between *Cxcl9*$^{+}$*Spp1*$^{-}$ and *Cxcl9*$^{-}$*Spp1*$^{+}$ macrophages.

(E) Bar plot shows differential enriched metabolism pathways between *Cxcl9*$^{+}$*Spp1*$^{-}$ and *Cxcl9*$^{-}$*Spp1*$^{+}$ macrophages by Gene set variance analysis (GSVA). *Cxcl9*$^{+}$*Spp1*$^{-}$ macrophages exhibited increased levels in TCA cycle and fatty acid metabolism, while *Cxcl9*$^{-}$*Spp1*$^{+}$ macrophages increased in glycolysis

and amino acid metabolism.

(F) Violin plot shows the different expressed genes between *Cxcl9*$^{+}$*Spp1*$^{-}$ and *Cxcl9*$^{-}$*Spp1*$^{+}$ macrophages in multiple pathways including IFN-gamma response and hypoxia.

(G) Scatter plot shows the validation of the correlation between some genes related to pathways in E-G and *Spp1*$^{+}$ macrophages in TCGA-LIHC. TPM data was used as the expression level of each gene, and normalized to immune cells by PTPRC expression.

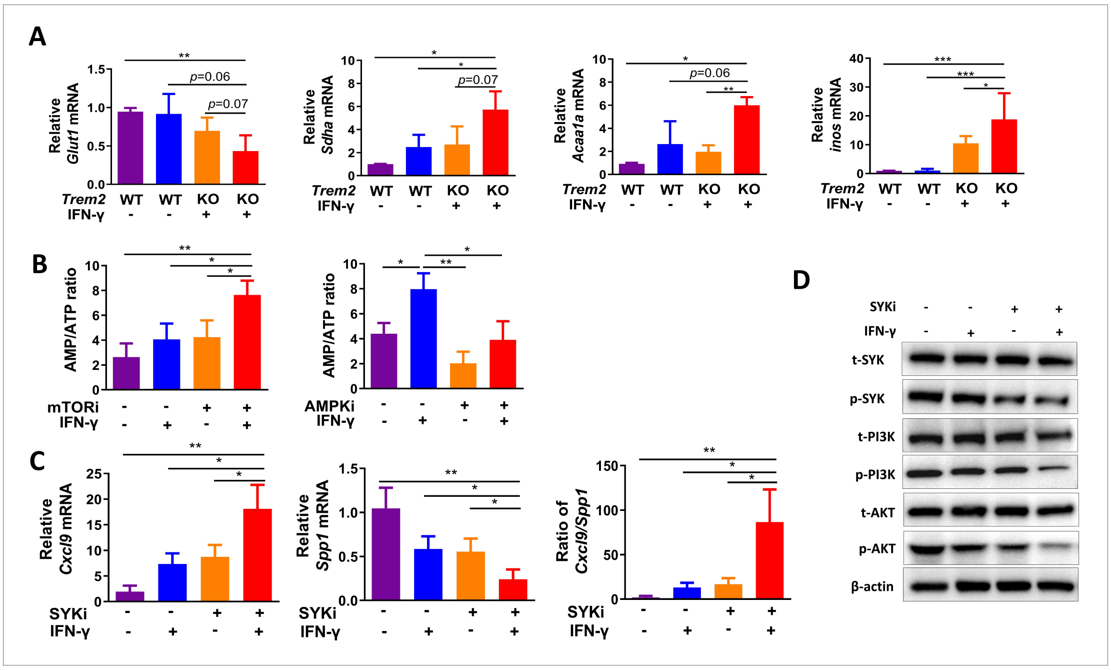

Figure S4. ***Trem2*-KO regulates metabolism by activating the AMPK signal pathway, related to Figure 5.**

(A) Relative mRNA expression levels of glycolysis, TCA and fatty acid oxidation marker genes (including *Ldha*, *Glut1*, *Sdha*, *Idh2*, Cpt1, and Acaa1a) in BMDMs were quantified by RT-qPCR. N=3, Wilcoxon tests, $*p<0.05$, $**p<0.01$, $***p<0.001$.

(B) AMP and ATP concentrations in BMDMs were measured using dedicated assay kits, and the AMP/ATP ratio was calculated from these values. N=3. Wilcoxon tests, $*p<0.05$, $**p<0.01$, $***p<0.001$.

(C) Relative mRNA expression levels of *Cxcl9, Spp1* in BMDMs were quantified by RT-qPCR, and the ratio of *Cxcl9/Spp1* was calculated. N=3. Wilcoxon tests, $*p<0.05$, $**p<0.01$, $***p<0.001$.

(D) BMDMs isolated from WT mice were educated for 24 hours in a transwell system with cancer cells, with or without IFN-γ (40 ng/mL). BMDMs were pre-treated with SYK inhibitor (R406, 1μM)

for 2 hours prior to co-culture. Relative protein expression levels of indicated molecular were assessed by WB and normalized to β-actin.

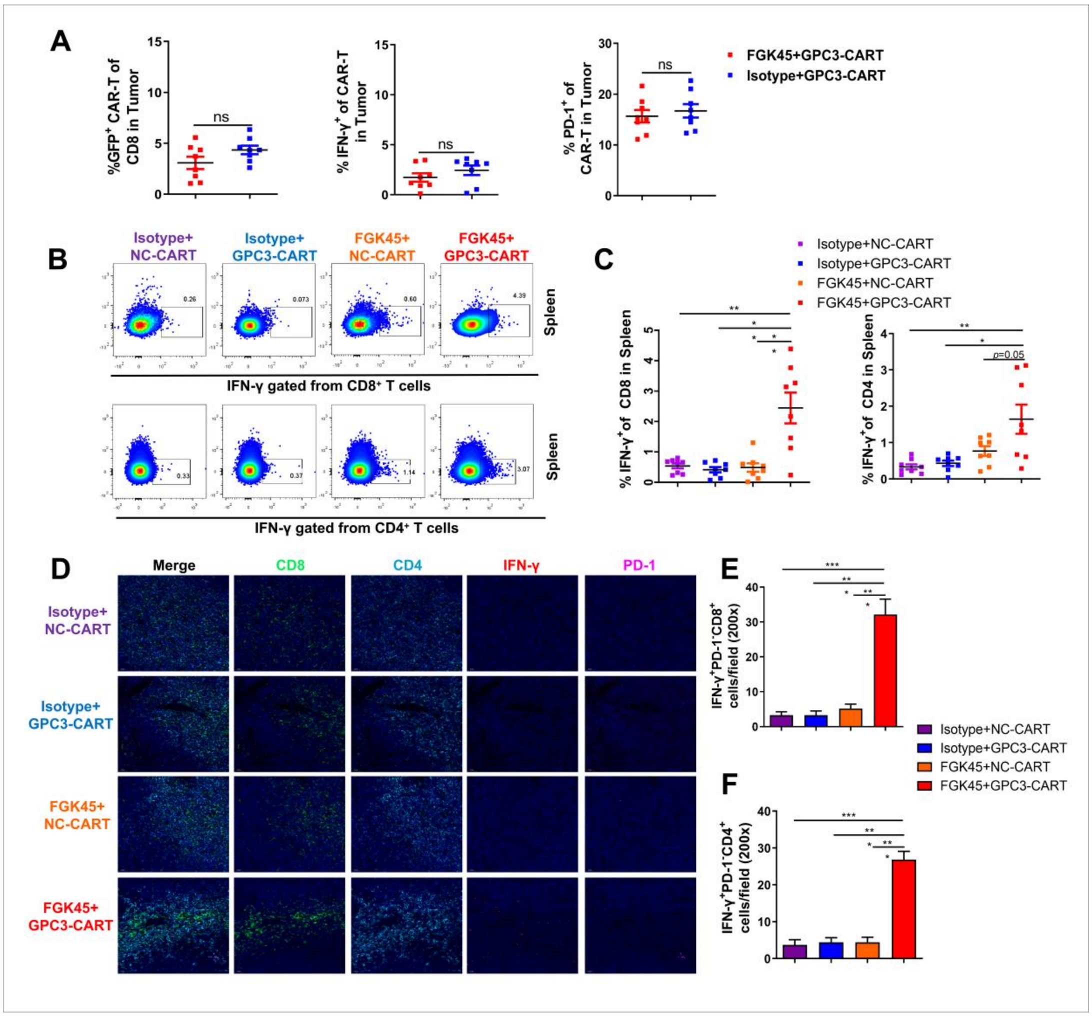

Figure S5. PD-1$^-$IFN-γ$^+$ CD8$^+$/CD4$^+$ T cells were increased in the splenic microenvironment of FGK45+GPC3-CAR-T group, related to Figure 7.

(A) Quantification of effector/exhausted CART cells from TILs, detecting by Flow cytometry. N = 8.

(B-C) Flow cytometry and quantification plots of IFN-γ$^+$CD8$^+$ T cells (effector CD8) and IFN-γ$^+$CD4$^+$ T cells (effector Th1), as well as IFN-γ$^-$CD8$^+$ T cells (exhausted CD8) and IFN-γ$^-$CD4$^+$ T cells (exhausted Th1) from spleen *in vivo*. N = 8.

(D-F) .Multiple immunofluorescence images of CD8$^+$ and CD4$^+$ T cells in spleen tissue between groups. Scale bar=50μm. Statistical plot representing the relative number of T cell subsets. (Quantification) Data are presented as mean ± SEM. The quantification of cell numbers was performed

by analyzing a minimum of three random low-power fields per condition per experiment. n = 3, *p <0.05, **p <0.01, ***p <0.001.

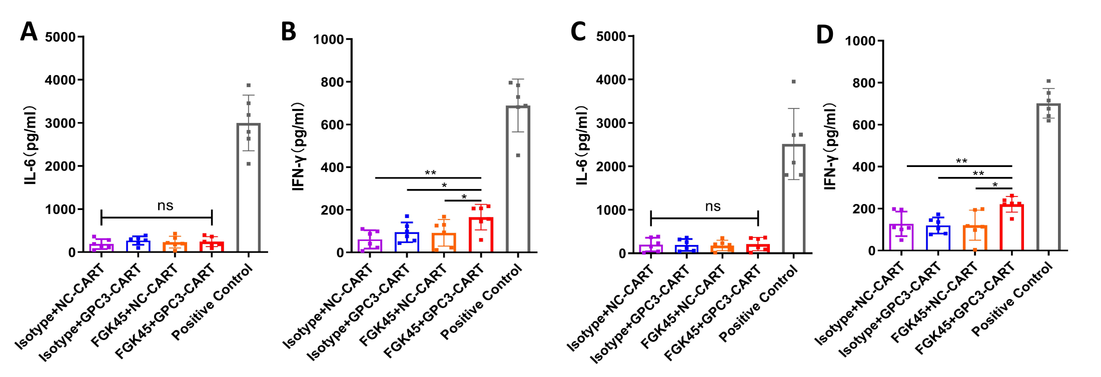

Figure S6. Safety Study of CD40 Agonist Combined with CAR-T Cell Therapy, related to Figure 7.

(A) Serum levels of mouse cytokines at 24 h following CAR T cell transfer (n= 6). A two-tailed unpaired two-sample t-test was used for statistical analysis. Positive control group for CRS. The murine lymphoma cell lines were transduced with recombinant retroviruses carrying human CD19. EL4 cells was used for C57 mice, and A20 cells was used for BALB/c mice. 3×10^6 cells was intraperitoneally injected into mice. Two weeks later, murine-derived αhCD19-CAR-T cells (3×10^7 cells) were adoptively transferred. After 24 hours, blood was collected and serum levels of mIL-6 and mIFN-γ were measured by ELISA.

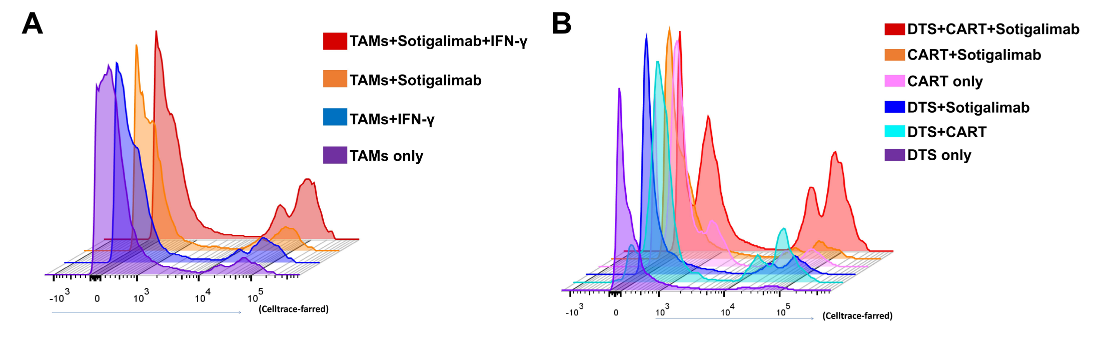

Figure S7. Detection of migrated $CD8^+$ T cells, related to Figure 7.

(A-B) Flow cytometry quantification of migrated $CD8^+$ T cells in different groups. (CellTrace-Far Red-labeled, n=5).

Table S1. Sequences of qRT-PCR Primers

Number	Gene	Primers
1	*Cxcl9*-5F	CCAGCCGAGGCACGATCC
2	*Cxcl9*-3R	ATCTCCGTTCTTCAGTGTAGCAATG
3	*Spp1*-5F	CCATCTCAGAAGCAGAATCTCCTTG
4	*Spp1*-3R	CTCCATCGTCATCATCATCGTCATC
5	*Ldha*-5F	GTGTGGAGTGGTGTGAATGTTGC
6	*Ldha*-3R	CTCCTTCCACTGCTCCTTGTCTG
7	*Idh2*-5F	GGATCATCTGGCAGTTCATCAAGG
8	*Idh2*-3R	CATTGGTCTGGTCACGGTTTGG
9	*Cpt1*-5F	ACGGCAGAGCAGAGGTTCAAG
10	*Cpt1*-3R	ACACCACATAGAGGCAGAAGAGG
11	*Glut1*-5F	TGAAAGAAGAGGGTCGGCAGATG
12	*Glut1*-3R	AGCACCACAGCGATGAGGATG
13	Sdha-5F	GGACATCAAGACTGGCAAGGTTAC
14	Sdha-3R	CAGTAGGAGCGGATAGCAGGAG
15	*Acaa1a*-5F	CCGCCTGCTGGAGAGTGAG
16	*Acaa1a*-3R	TCTGCCGTGAAATGCCAAACC
17	*Inos*-5F	TCACTCAGCCAAGCCCTCAC
18	*Inos*-3R	TCCAATCTCTGCCTATCCGTCTC

Data S1. Unedited gel images

Full unedited gel for Figure 5F

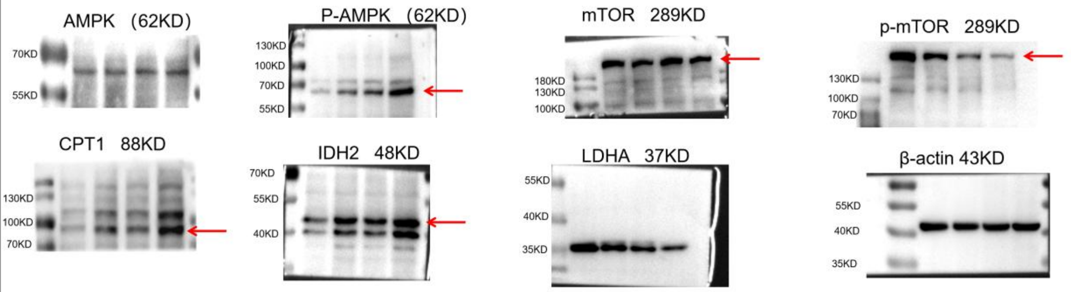

Full unedited gel for Figure 5G

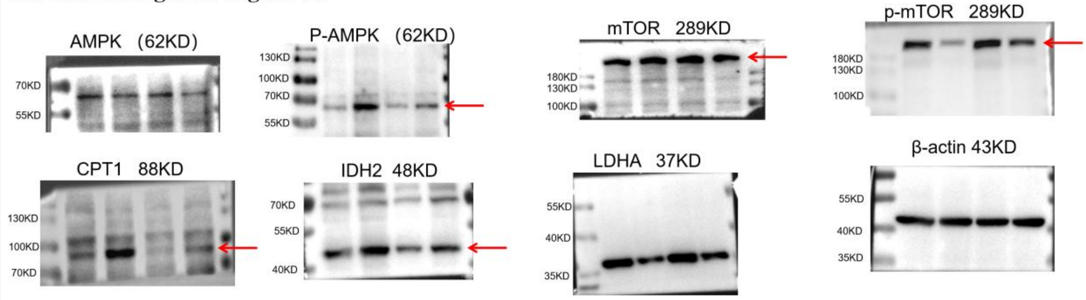

Full unedited gel for Figure 5H

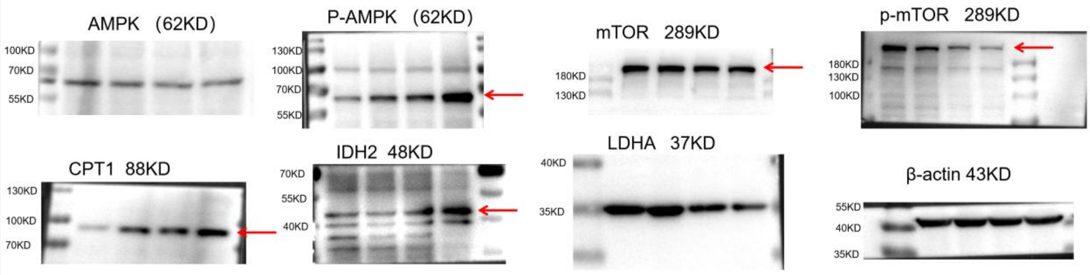

Full unedited gel for Figure 5M

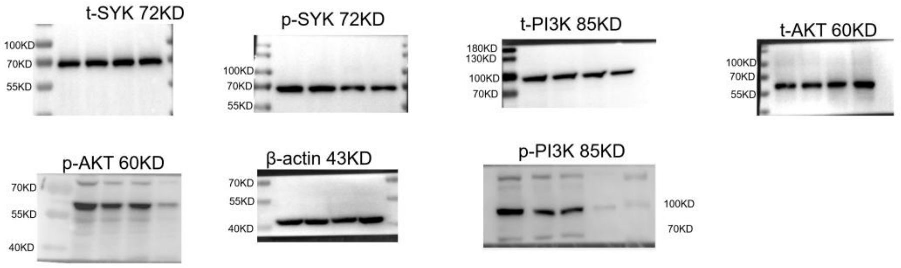

Full unedited gel for supplemental Figure S4D

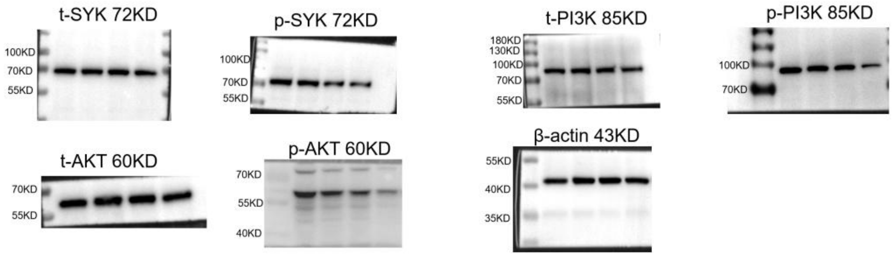

Full unedited gel for Figure 5N

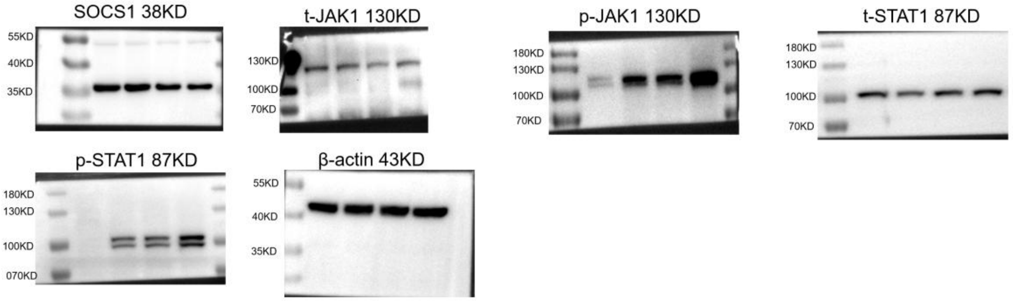

Full unedited gel for Figure 5O

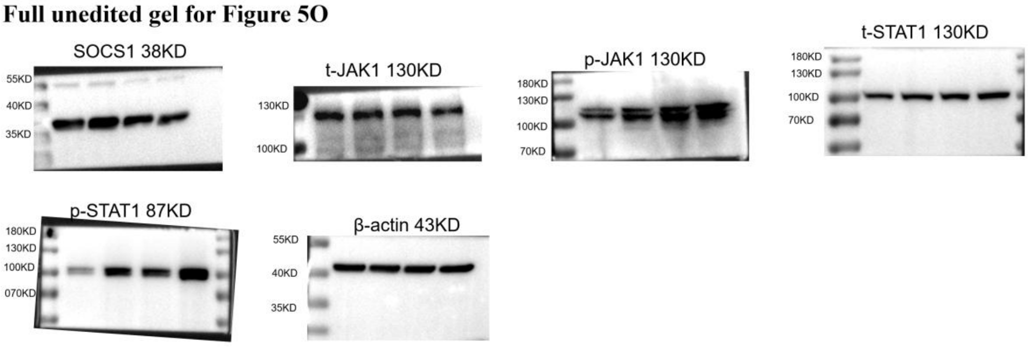

Full unedited gel for Figure 5P

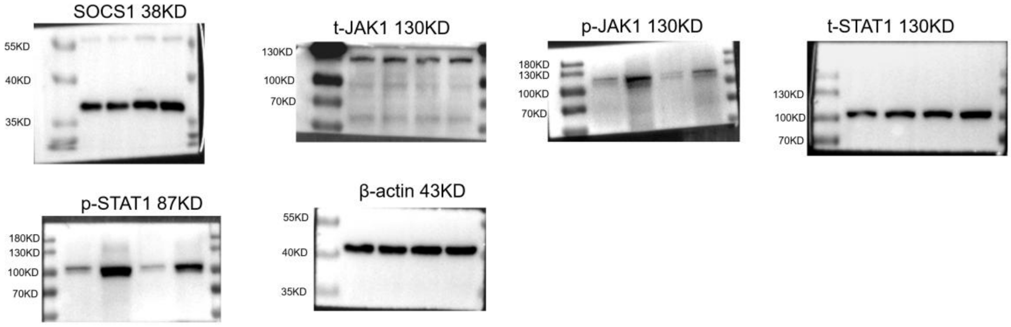

Full unedited gel for Figure 6K

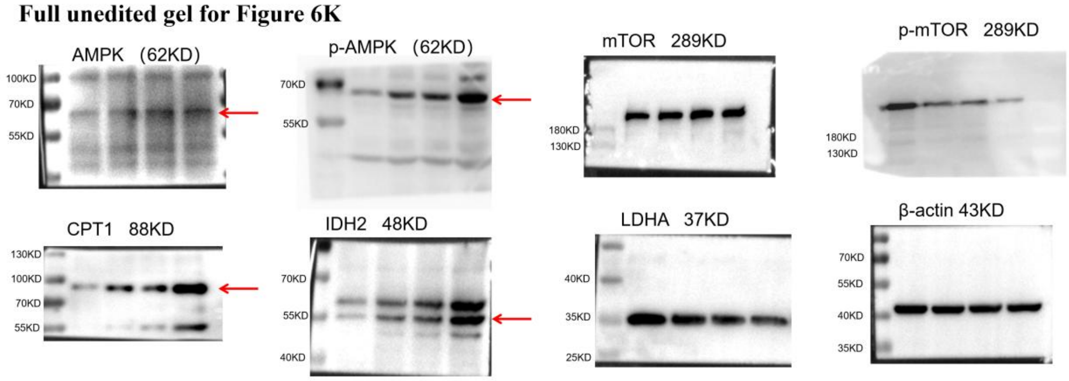

Full unedited gel for Figure 6L

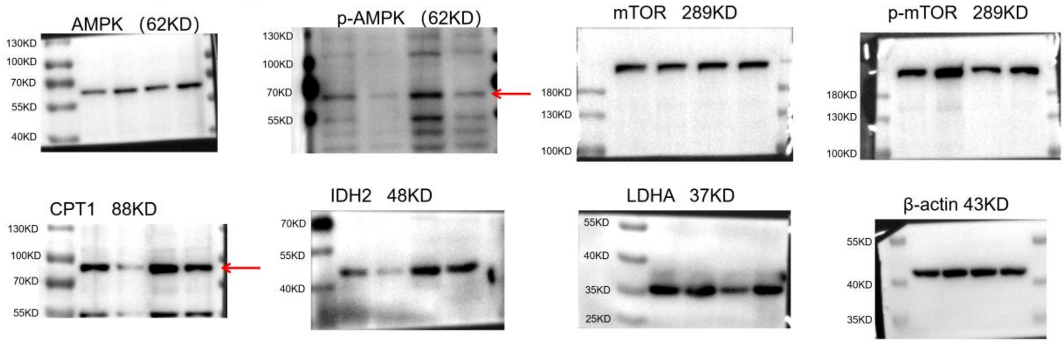

Full unedited gel for Figure 6D

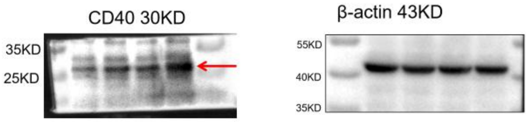

Full unedited gel for Figure 6O

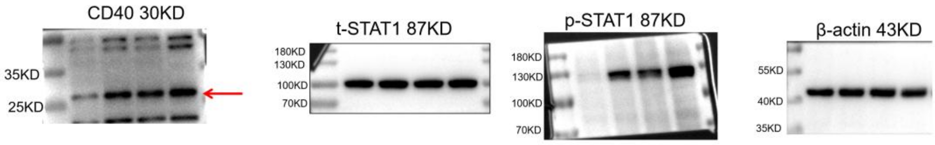

Full unedited gel for Figure 6P

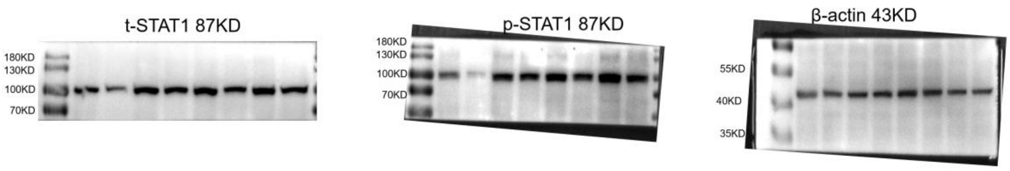

Supplement: Document S1. Figures S1–S7, Table S1, and Data S1 [file mmc1.pdf]
