# Supplementary material for: CAR-T triggers TAM reeducation and adaptive anti-tumor response via TREM2 deficiency or CD40 agonist
Source: Cell Rep Med. 2026 Jan 20;7(1):102539. doi: 10.1016/j.xcrm.2025.102539 (PMC12866111; doi:10.1016/j.xcrm.2025.102539)

Cell Reports
Medicine

Article

CAR-T triggers TAM reeducation and adaptive anti-tumor response via *TREM2* deficiency or CD40 agonist

Graphical abstract

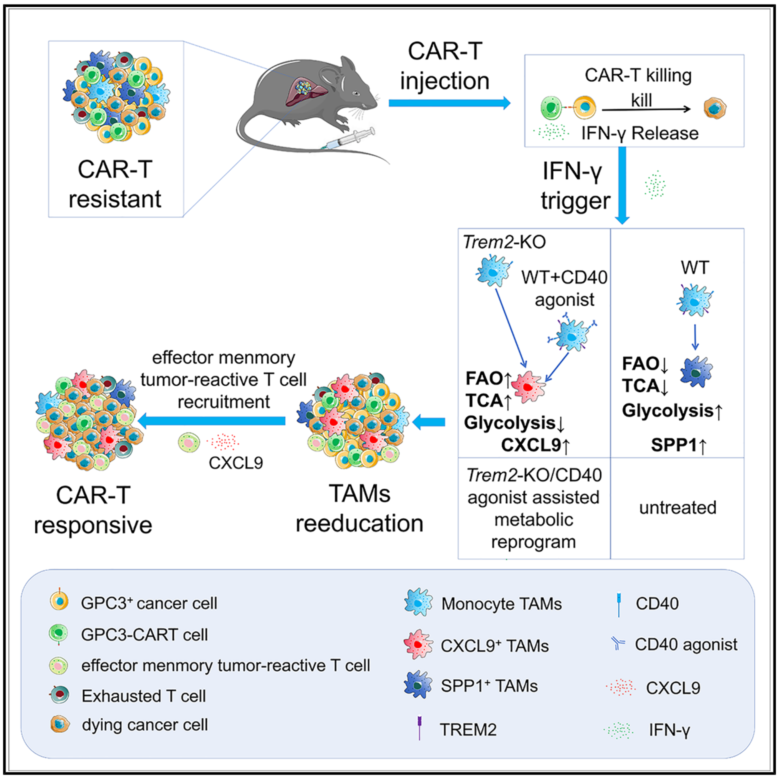

Highlights

- TREM2$^+$ macrophages drive GPC3-CAR-T resistance in hepatocellular carcinoma
- *Trem2* deficiency reprograms TAM metabolism and activates tumor-specific CD8$^+$ T cells
- CD40 agonism mimics *Trem2*-KO via AMPK activation and STAT1-driven CXCL9 production
- Sotigalimab enhances human CD8$^+$ T cell migration in combination with CAR-T

Authors

Ting Liu, Huixin Gao, Zhihui Xi, ..., Xianzhang Huang, Wenzhe Fan, Jizhou Tan

Correspondence

huangxz020@163.com (X.H.), fwzhe@mail.sysu.edu.cn (W.F.), tanjzh@mail.sysu.edu.cn (J.T.)

In brief

Ting Liu et al. show that TREM2$^+$ macrophages drive resistance to GPC3-CAR-T therapy in hepatocellular carcinoma. Targeting TREM2—or using a CD40 agonist—overcomes resistance by triggering TAM reeducation and adaptive anti-tumor response, proposing a promising combination strategy to boost CAR-T efficacy.

Liu et al., 2026, Cell Reports Medicine 7, 102539
January 20, 2026 © 2025 The Authors. Published by Elsevier Inc.
https://doi.org/10.1016/j.xcrm.2025.102539

CellPress

Cell Reports Medicine

CellPress
OPEN ACCESS

Article

CAR-T triggers TAM reeducation and adaptive anti-tumor response via *TREM2* deficiency or CD40 agonist

Ting Liu,[1,6,7] Huixin Gao,[3,7] Zhihui Xi,[4,5,7] TianTian Yu,[2] Yimei Gu,[1] Hanbing Mai,[1] Hui Yuan,[1] Yafang Liu,[1] Haikuan Liu,[2] Qiaoxuan Zhang,[1] Xianzhang Huang,[1,6,*] Wenzhe Fan,[2,*] and Jizhou Tan[2,8,*]

[1]The Second Clinical Medical College, Guangzhou University of Chinese Medicine, Clinical Laboratory/State Key Laboratory of Traditional Chinese Medicine Syndrome, Guangdong Provincial Hospital of Chinese Medicine, Guangzhou, Guangdong 510120, China
[2]The First Affiliated Hospital, Sun Yat-sen University, Guangzhou, Guangdong 510080, China
[3]Department of Clinical Laboratory, Guangzhou Women and Children Medical Center, Guangzhou Medical University, Guangzhou, Guangdong 510600, China
[4]School of Medicine, South China University of Technology, Guangzhou, Guangdong 510006, China
[5]Department of Gastrointestinal Surgery, Guangdong Provincial People's Hospital (Guangdong Academy of Medical Sciences), Southern Medical University, Guangzhou, Guangdong 510080, China
[6]State Key Laboratory of Dampness Syndrome of Chinese Medicine, Guangdong Provincial Hospital of Chinese Medicine, Guangzhou, Guangdong 510120, China
[7]These authors contributed equally
[8]Lead contact
*Correspondence: huangxz020@163.com (X.H.), fwzhe@mail.sysu.edu.cn (W.F.), tanjzh@mail.sysu.edu.cn (J.T.)
https://doi.org/10.1016/j.xcrm.2025.102539

SUMMARY

Chimeric antigen receptor (CAR)-T therapy targeting GPC3 shows unsatisfactory clinical efficacy in hepatocellular carcinoma (HCC). Combining clinical data and the immunocompetent orthotopic HCC model, we demonstrate that TREM2$^+$ tumor-associated macrophages (TAMs) are critical mediators of GPC3-CAR-T resistance. We find that Trem2 deficiency synergizes with GPC3-CAR-T to enhance tumor control by expanding endogenous tumor-specific CD8$^+$ T cells (not CAR-T amplification) and reeducating TAMs to an anti-tumor CXCL9^{hi}/SPP1^{lo} phenotype via metabolic reprogramming. Mechanistically, this combination enhances oxidative metabolism while suppressing glycolysis through JAK-STAT1 triggering, AMPK activation, and PI3K-AKT-mTOR inhibition. Crucially, Trem2 deficiency up-regulates CD40 expression, enabling CD40 agonism to phenocopy Trem2-deficiency effects via AMPK activation and STAT1-driven CXCL9 production. Notably, the clinical agonist sotigalimab similarly enhances human CD8$^+$ T cell migration *in vitro*. Our findings highlight the significance of combining GPC3-CAR-T therapy with CD40 agonist as a critical pre-requisite for eliciting reeducation of TAMs and enhancing the efficacy of CAR-T therapy in HCC.

INTRODUCTION

Hepatocellular carcinoma (HCC) is the most common form of liver cancer.[1] Current immunotherapy options for HCC, particularly cellular therapy, have shown limited effectiveness, which underscores the highly immunosuppressive nature of the hepatic tumor microenvironment (TME).[2] Glypican-3 (GPC3) demonstrates predominant expression in HCC cells, while being virtually absent in normal tissues. The distinctive characteristic should position GPC3-targeted chimeric antigen receptor (CAR)-T cell therapy as one of the most promising therapeutic strategies for HCC.[3–5] However, CAR-T cells have limited efficacy in solid tumors, including HCC.[5–7] David Steffin et al. found that the conventional GPC3-CAR-T was safe but produced no objective antitumor responses and reached peak expansion at 2 weeks.[8] The efficacy of CAR-T cells was limited in solid tumors due to the TME, which contained inhibitory signals that block immune responses.[9,10] Therefore, remodeling the TME of HCC to enhance GPC3-CAR-T efficacy will emerge as a critical breakthrough in this field.[11,12] Tumor-associated macrophages (TAMs) represent a crucial cellular component within the TME. Importantly, the functional modification of TAMs toward either anti- or pro-tumor phenotypes can be modulated through external interventions, a process termed *macrophage reeducation*.[13,14] Marit J. van Elsas et al. showed that immunotherapy induced skewed late-stage-activated M1-like macrophages via CCR5 signaling and recruitment of intratumoral CD8$^+$ T cells, which were critical for effective tumor control.[14] The synergistic cooperation between adaptive CD8$^+$T cells and anti-tumor TAMs is indispensable for effective immunotherapy, providing novel strategic insights for combination therapies.

Triggering receptor expressed on myeloid cells 2 (TREM2) is a molecule expressed on TAMs and has been reported to induce immunosuppression in many solid tumors, especially HCC.[15–17]

This is an open access article under the CC BY license (http://creativecommons.org/licenses/by/4.0/).

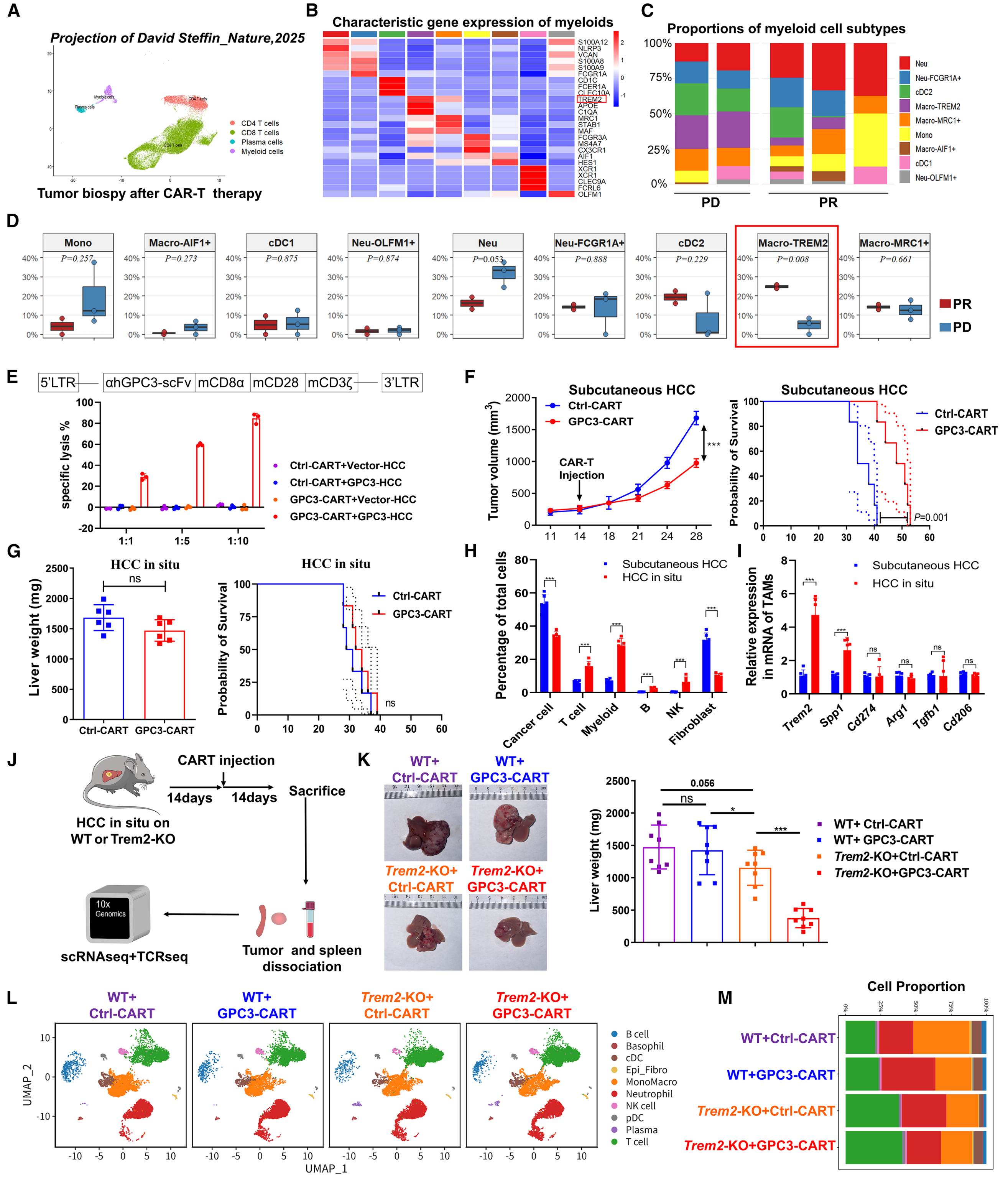

Figure 1. TREM2^{+} TAMs hinder the therapeutic effectiveness of GPC3-CAR-T cells

(A) UMAP visualization of scRNA-seq data from tumor biopsies following IL15-GPC3-CAR-T therapy (data from Steffin et al., *Nature* 2025; GEO: GSE253352).

(B) The heatmap displayed the signature differentially expressed genes across distinct myeloid cell subpopulations.

(C) The bar chart showed the proportions of myeloid cell subsets in the PD and PR groups.

(D) The boxplot demonstrated the statistical differences among various myeloid subpopulations.

(legend continued on next page)

Trem2 knockout (KO) or blockade can enhance antitumor immunity.[18,19] Our previous work revealed that TREM2$^+$ TAMs suppressed the efficacy of immune checkpoint blockers in HCC.[20] Based on this, we hypothesized that TREM2$^+$ TAMs in HCC may constitute a key immunosuppressive component within the TME that functionally impairs GPC3-CAR-T cell efficacy and *Trem2-KO* potentially enhances the therapeutic efficacy of CAR-T cells.

In the mechanistic studies, it was discovered that *Trem2*-KO combined with CAR-T therapy promoted the formation of anti-tumor TAMs, which secreted abundant CXCL9 to recruit endogenous CD8$^+$ T cells for sustained tumor killing. However, in clinical settings, TREM2 inhibitors carry the risk of central neurotoxicity. TREM2-dependent microglia hyper-activation promotes neuroprotection, while *Trem2* deficiency elevates synaptic loss and neurodegenerative biomarkers in cerebrospinal fluid.[21] The neuroprotective functions of microglia are closely associated with TREM2, as mutations or deficiency in TREM2 can increase the risk of neurodegenerative disorders including Alzheimer's disease (AD) and frontotemporal dementia-like syndromes.[22–24] Therefore, we have identified an alternative approach to TREM2 blockade that has demonstrated both safety and efficacy in clinical settings. Notably, we found that CD40 was highly expressed on *Trem2-KO* TAMs and it shared the transcription factors with CXCL9. This finding suggested that CD40 agonism combined with CAR-T therapy may similarly induce the generation of such anti-tumor TAMs, thereby achieving an effect similar to that of *Trem2-KO*. Studies had shown that CD40 agonism can activate and stimulate the expression of pro-inflammatory marker genes in TAMs, driving reeducation of TAMs toward an anti-tumor phenotype.[25–27] Pu-Ste Liu et al. found that CD40 signaling promoted fatty acid oxidation (FAO) to trigger the epigenetic reprogramming of anti-tumor polarization of macrophage.[28]

Therefore, we postulated that *Trem2*-KO and CD40 agonism might share mechanistically similar pathways in reeducating TAMs. This suggested that combining GPC3-CAR-T therapy with either *Trem2*-KO or CD40 agonism could represent a promising therapeutic paradigm to substantially enhance immunotherapeutic outcomes.

RESULTS

TREM2$^+$ TAMs hinder the therapeutic effectiveness of GPC3-CAR-T cells

A recent clinical trial revealed that the GPC3-CAR-T therapy demonstrated unsatisfactory efficacy in HCC treatment,[8] that the conventional GPC3-CAR-T therapy showed a 0% objective response rate (ORR), and that the IL-15-modified GPC3-CAR-T achieved a 30% ORR. Single-cell sequencing data of the above study was obtained from the Gene Expression Omnibus (no. GSE253352), which was based on tumor biopsy samples after taking IL-15-GPC3-CAR-T therapy (Figure 1A). Myeloid cells were categorized into nine subpopulations, with their subset-specific gene signatures also characterized (Figure 1B). Macro-TREM2 was identified as the only myeloid subset showing significant intergroup differences between progressive disease (PD) and partial response (PR) groups (p = 0.008); they were highly enriched in the PD group (Figures 1C and 1D). This suggests that TREM2$^+$ macrophages in HCC immune microenvironment may contribute to GPC3-CAR-T resistance. To verify this and investigate the underlying mechanism of CAR-T resistance in orthotopic liver TME, the Hepa1-6 tumor cell line stably expressing human GPC3 was established in the immunocompetent murine model. The generated human-GPC3-targeted CAR-T cells from murine CD8$^+$ T cells were used to evaluate tumor-killing activity. *In vitro* experiments demonstrated that GPC3-CAR-T cells exhibited potent cytotoxicity against GPC3-HCC cells (Figure 1E). Subcutaneous *in vivo* experiments demonstrated that GPC3-CAR-T cells significantly suppressed tumor growth and prolonged survival in immunocompetent mice (Figure 1F). Notably, the antitumor efficacy of GPC3-CAR-T cells was significantly attenuated in orthotopic HCC models, exhibiting limited growth-inhibitory effects (Figure 1G), consistent with the clinical study.[8] This discrepancy may stem from the liver-specific immunosuppressive niche. Therefore, we analyzed the proportion of immune cell subsets between these two models by flow cytometry. It revealed an increase in myeloids in orthotopic HCC, which may be associated with the CAR-T resistance (Figure 1H). Quantitative reverse-transcription PCR (RT-qPCR) analysis of sorted TAMs revealed a significant up-regulation of *Trem2* gene expression in orthotopic HCC (Figure 1I), suggesting that TREM2$^+$ TAMs acted as a barrier hindering GPC3-CAR-T therapy in orthotopic HCC. For mechanistic exploration, orthotopic HCC was induced in *Trem2-KO* mice, followed by GPC3-CAR-T infusion. Tumor tissues and spleens were processed for single-cell RNA sequencing (scRNA-seq) at progression endpoints (Figures 1J and S1A–S1D). It revealed that the *Trem2*-KO mice following GPC3-CAR-T intervention exhibited significantly enhanced tumor control, while wild-type (WT) mice displayed marginal therapeutic response (Figure 1K). Uniform manifold approximation and projection (UMAP) visualization and cellular proportion analysis revealed an

(E) *In vitro* cytotoxicity measured by lactate dehydrogenase (LDH) release at indicated effector cell vs. target cell ratios (E:T ratios) using Hepa1-6 cells over-expressing human GPC3 or vector control were co-cultured with Ctrl-CAR-T (targeted CD19) or GPC3-CAR-T cells.
(F) Subcutaneous tumor growth and survival in C57BL/6J mice (n = 6/group) inoculated with GPC3-Hepa1-6 cells and treated with Ctrl- or GPC3-CAR-T cells (1×10^6) via tail vein.
(G) Orthotopic liver tumor model: tumor weight at day 28 and survival in mice receiving intrahepatic GPC3-Hepa1-6 implants followed by CAR-T treatment.
(H) Flow cytometric analysis of immune and stromal cell populations in tumors from subcutaneous and orthotopic models following GPC3-CAR-T treatment.
(I) Expression of TAM-associated genes (Trem2, Spp1, Cd274, Arg1, Tgfb1, and Cd206) in F4/80$^+$ TAMs sorted from tumors of GPC3-CAR-T-treated mice, measured by RT-qPCR.
(J) Schematic of the orthotopic HCC model for CAR-T therapy in *Trem2*-WT/KO mice.
(K) Tumor representative photographs of different groups were shown. Dot plots showed liver weight, liver-to-body weight ratio, per liver, n = 8.
(L) UMAP plots of individual groups displayed distinct intratumoral cell populations. n = 2 for all treatment groups on scRNA sequence.
(M) Proportion of the distinct intratumoral cell populations. Data were represented by mean ± SEM. ns, no significance, $*p < 0.05$, $**p < 0.01$, $***p < 0.001$.

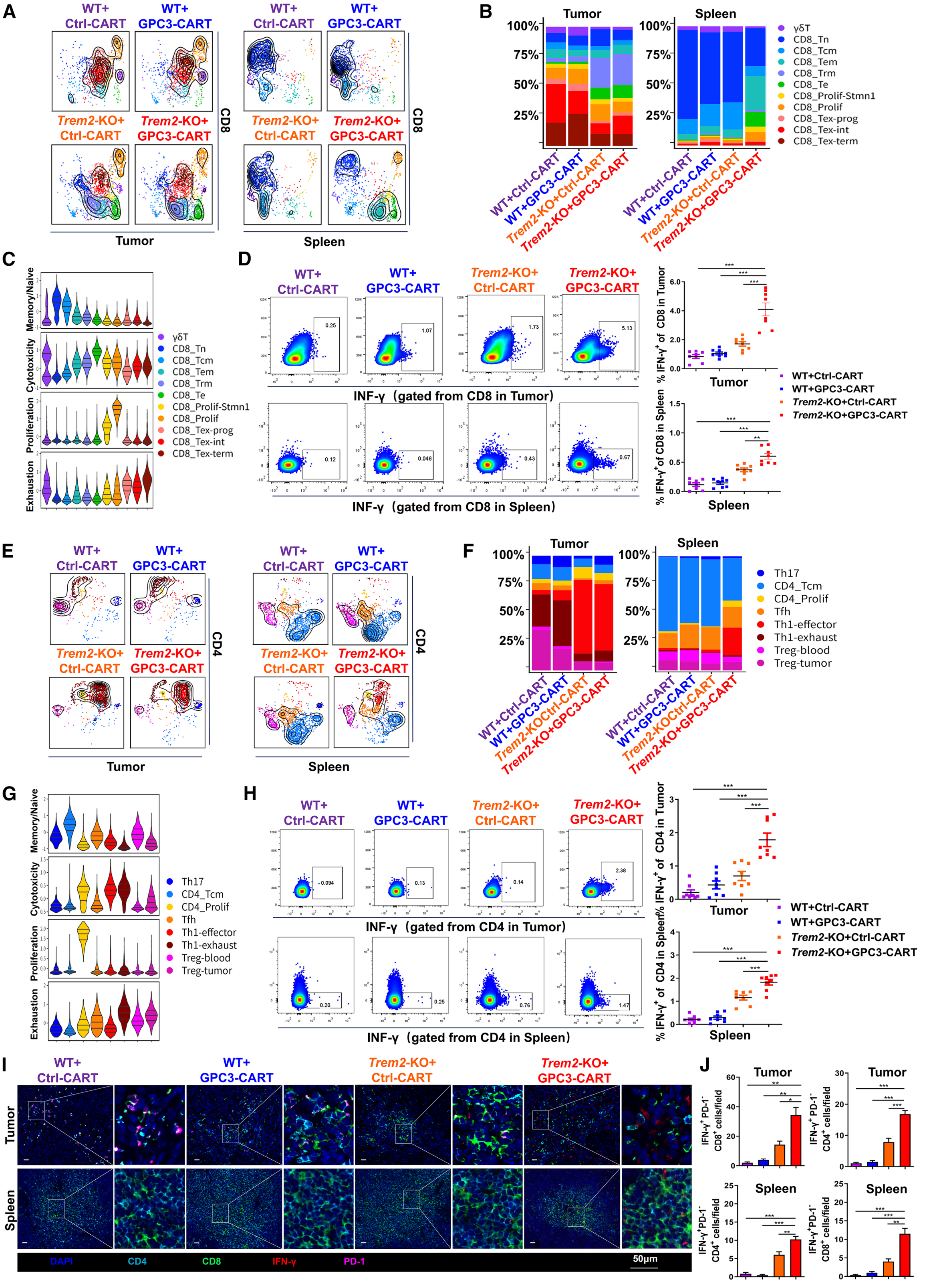

(legend on next page)

increase in T cell infiltration in *Trem2*-KO tumors, and both *Trem2*-KO samples were similar (Figures 1L and 1M), suggesting potential alterations in immune cell landscape.

The dual intervention of *Trem2*-KO and GPC3-CAR-T induced profound remodeling of T cell ecosystems in both tumor and splenic microenvironments

Single-cell subpopulation analysis stratified $CD8^+$ T cells into 11 transcriptionally unique subsets (Figures S1C, S1E, and S1G). Subpopulation analysis of $CD4^+$ T cells at single-cell resolution identified 8 heterogeneous clusters (Figures S1D, S1F, and S1H). Clustering methodology followed the established protocols.[29–31] Cellular proportion analysis revealed that *Trem2*-KO increased CD8_Trm (tissue-resident memory) and CD8_Te (effector cytotoxic) subsets, while depleting CD8_Tex (exhausted) populations in tumors. Combined *Trem2*-KO and GPC3-CAR-T therapy increased the proportions of CD8_Te and CD8_Tem (effector memory) subsets in the spleen (Figures 2A and 2B). These increased CD8 subsets exhibited enhanced cytotoxic phenotype and minimal exhaustion levels (Figure 2C). Flow cytometric validation revealed a significant expansion of IFN-$\gamma^+$$CD8^+$ T cell populations in both tumor and splenic compartments following combined *Trem2*-KO and GPC3-CAR-T therapy (Figure 2D). Cell proportion analysis revealed that *Trem2*-KO significantly expanded the Th1_effector compartment and concurrently diminished Th1_exhausted and Treg_tumor subsets in the TME. Notably, combinatorial therapy of *Trem2*-KO and GPC3-CAR-T synergistically augmented the Th1_effector subset in splenic lymphocytes (Figures 2E and 2F). Importantly, the Th1_effector subset exhibited robust cytotoxic potential while maintaining minimal exhaustion level, suggesting enhanced antitumor phenotypic characterization (Figure 2G). Flow cytometry confirmed significant expansion of IFN-$\gamma^+$$CD4^+$ T cells in both tumor and spleen following combined *Trem2*-KO and GPC3-CAR-T therapy (Figure 2H). Multiplex immunofluorescence consistently demonstrated co-localized accumulation of IFN-γ^+PD-$1^-$$CD8^+$ and IFN-γ^+PD-$1^-$$CD4^+$ T cells in both tumor parenchyma and splenic white pulp following *Trem2*-KO and GPC3-CAR-T combination therapy (Figures 2I and 2J). This dual intervention reshaped both adaptive immune compartments toward enhanced effector functionality with reduced exhaustion, suggesting potent synergy for antitumor immunity.

The dual intervention of *Trem2*-KO and GPC3-CAR-T promoted the expansion of non-exhausted tumor-specific T cells

To investigate the cellular origin of the expanded effector subpopulations resulting from the dual intervention, we performed analysis on CAR-T cell components and the T cell receptor (TCR) sequencing profiling on non-CAR-T cells. Results showed CAR-T cells were predominantly enriched in the TME, with the majority localized on the exhausted subpopulations (Figures 3A and 3B). Surprisingly, we found no significant differences in either the exhaustion score or cytotoxicity score of CAR-T cells between *Trem2*-KO and WT groups (Figure 3C). Flow cytometry validation further demonstrated no significant differences in either the quantity of CAR-T cells or the proportion of IFN-γ^+CAR-T cells between the above two groups (Figure 3D). These findings indicated that the expansion of the effector T cell subpopulations we identified originated from endogenous adaptive immune T cells, most likely tumor-specific T cells. We scored the anti-tumor activity using multiple tumor-specific T cell markers (including *ENTPD1*, *ITGAE*, *CXCR6*, *PDCD1*, *TNFRSF9*, *HAVCR2*, and GZMB),[32–34] revealing that tumor-specific $CD8^+$T cells were predominantly enriched in CD8_Tex, and partial CD8_Te/Trm subpopulations (Figure 3E). It showed strong transcriptional similarities to the experimentally confirmed tumor-reactive tumor-infiltrating lymphocytes (TILs), whereas T-naive/Tcm (central memory) cluster was enriched in signatures of T cells with reported viral antigen-specific TILs.[32,35] The TCR repertoire profiling of these tumor-specific $CD8^+$T cells, including clonotype distribution and clone sequence, further corroborated these findings (Figure 3F). To further validate, we classified all expanded tumor-specific TCR clones into four categories based on clonal abundance: large, medium, small, and single (Figure 3G). Notably, the combination intervention group exhibited a higher proportion of large and medium TCR clones, with preferential enrichment in CD8_Te/Trm subsets. Moreover, the splenic TCR clonal expansion in the combination therapy group surpassed that of other groups, with these expanded clones exclusively belonging to CD8_Te subset (Figure 3H). These results indicated that the dual intervention not only enhanced tumor-specific TCR clonal expansion but also improved functional quality, suggesting superior anti-tumor score (Figure 3I). The analysis of $CD4^+$T cells similarly revealed a higher proportion of medium-sized TCR clones in the combination therapy group, with predominant enrichment in the Th1-effector subset (Figures S2A–S2C).

Figure 2. The dual intervention of *Trem2*-KO and GPC3-CAR-T induced profound remodeling of T cell ecosystems in both tumor and splenic microenvironments

(A) UMAP clusters showed projection of cells onto a reference $CD8^+$T cell atlas (colored T cell subtypes) in tumor and spleen.

(B) Proportion of the distinct $CD8^+$ TILs and intrasplenic $CD8^+$ T cell subtypes in tumor and spleen.

(C) Violin plots revealed cell function based on each cell state scored by gene expressions of exhaustion, proliferation, cytotoxicity, and memory/naive phenotypes from $CD8^+$T subtypes in tumor, with medians and quartiles indicated.

(D) Flow cytometry and quantification plots of IFN-$\gamma^+$$CD8^+$ T cells (effector CD8) in tumor and spleen. $n = 8$.

(E) UMAP clusters showed projection of cells onto a reference $CD4^+$T cell atlas (colored T cell subtypes) in tumor and spleen.

(F) Proportion of the distinct $CD4^+$ TILs and intrasplenic $CD4^+$ T cell subtypes in tumor and spleen.

(G) Violin plots revealed cell function based on each cell state scored by gene expressions of exhaustion, proliferation, cytotoxicity, and memory/naive phenotypes from $CD4^+$T subtypes in tumor, with medians and quartiles indicated.

(H) Flow cytometry and quantification plots of IFN-$\gamma^+$$CD4^+$ T cells in tumor and spleen. $n = 8$.

(I and J) Multiple immunofluorescence images of tumor and spleen tissue between groups. Scale bars, 50 μm. Quantification of cells numbers from three random fields per mouse ($n = 8$ mice/group). Data were represented by mean ± SEM. $^*p < 0.05$, $^{**}p < 0.01$, $^{***}p < 0.001$.

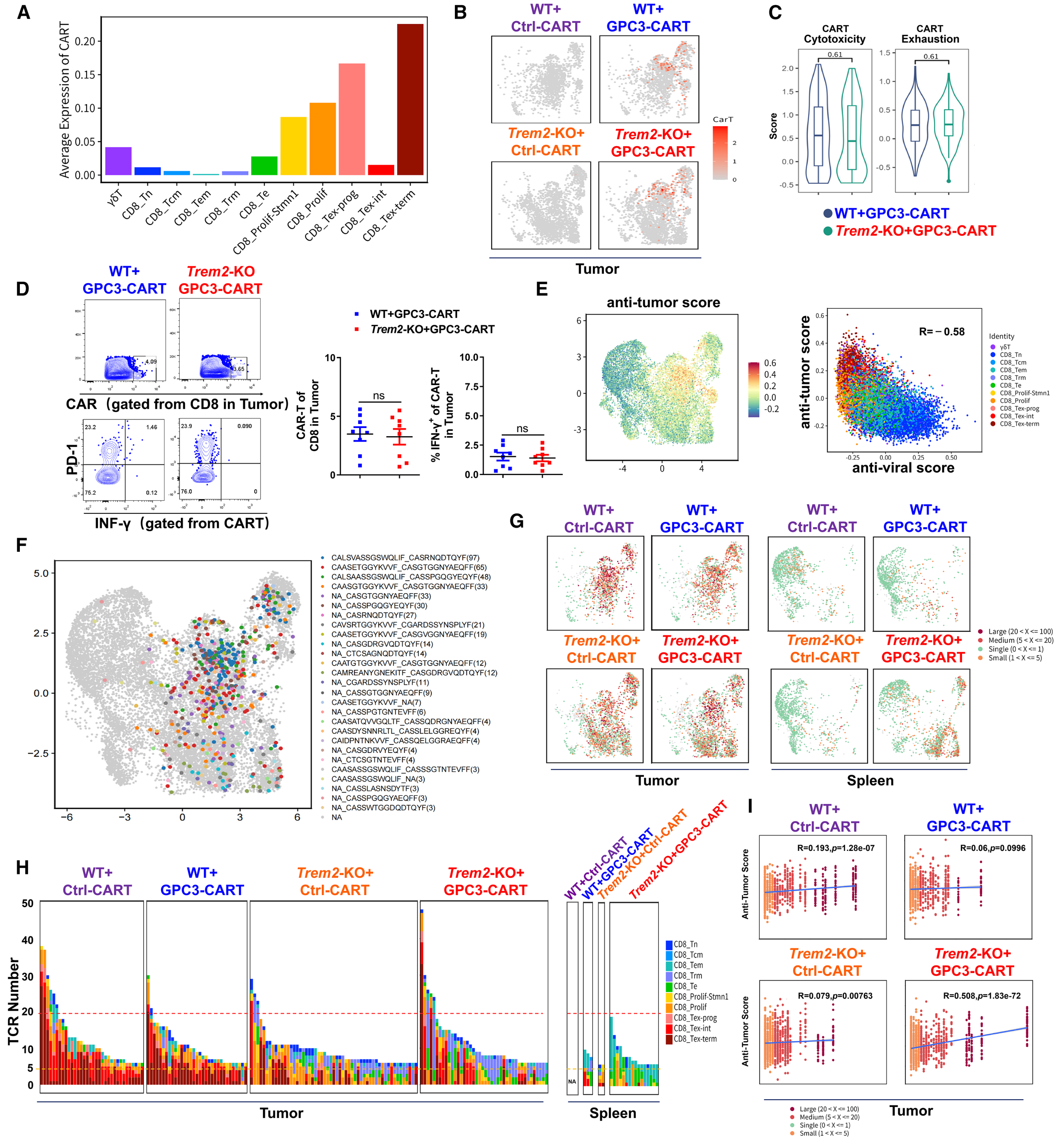

Figure 3. The dual intervention of *Trem2*-KO and GPC3-CAR-T promoted the expansion of non-exhausted tumor-specific T cells

(A) The average expression of CAR-T cells among different CD8$^+$ subsets.

(B) Normalized expression of the indicated GFP or CAR sequence among different groups.

(C) Violin plots showed the cytotoxicity and exhaustion for CAR-T cells.

(D) Flow cytometry plots and quantification of CAR-T cells from TILs. $n = 8$.

(E) UMAPs of CD8$^+$ T cells were colored based on the score of the published gene signatures of tumor-specific CD8$^+$ TILs and demonstrated by antitumor score (left plot). The right plot revealed the correlation between antiviral score (based on the score of the published gene signatures of bystander CD8$^+$ TILs) and antitumor score among different CD8$^+$ subsets.

(F) UMAPs of TCR clone types recognized from tumor-specific CD8$^+$ TILs in (E). The sequences of TCRs and their numbers were revealed.

(legend continued on next page)

These results demonstrated that dual targeting of *Trem2*-KO and GPC3-CAR-T synergistically expanded non-exhausted tumor-specific T cell clones, primarily by enhancing endogenous adaptive immunity rather than modulating CAR-T cell functionality. The TAM reprogramming may play a significant role in this process.

Trem2-KO combined with GPC3-CAR-T derived TAM reprogramming toward anti-tumor phenotype

TAMs were re-clustered into 14 sub-populations (Figures 4A and S3A). The study found that the *Trem2*-KO combined with GPC3-CAR-T treatment group exhibited the highest proportion of Macro_*Cxcl9* subset and the lowest proportion of Macro_*Spp1* subset (Figures 4B and S3B). Importantly, the *Cxcl9*/*Spp1* ratio was the highest in the *Trem2*-KO combined with GPC3-CAR-T treatment group, and this ratio had been confirmed as a key predictive biomarker for immunotherapy efficacy[36] (Figures 4C, S3C, and S3D). The immunofluorescence analysis in murine models demonstrated a significant reduction in SPP1$^+$TAMs but an increase in CXCL9$^+$TAMs, along with an elevated CXCL9/SPP1 ratio, in the *Trem2*-KO mice treated with GPC3-CAR-T therapy (Figures 4D and 4E). Clinically, the analysis of liver hepatocellular carcinoma (LIHC) data from The Cancer Genome Atlas (TCGA) database revealed that a high CXCL9/SPP1 ratio was associated with longer overall survival (Figure 4F). To confirm this finding, 12 HCC patients treated with transcatheter arterial chemoembolization (TACE) plus anti-PD-1 antibody afterward were included into study and were divided into responder and no-responder groups (Figure 4G). The numbers of CXCL9$^+$TAMs in the responder group were more than those in the no-responder group; the reverse is true for SPP1$^+$ TAMs. The ratio of CXCL9/SPP1 in the responder group was also higher than in the no-responder group (Figures 4H and 4I). These results disclosed that CXCL9/SPP1 ratio might be a potential prognostic indicator in HCC immunotherapy therapy.

Trem2-KO combined with IFN-γ promoted metabolic reprogramming of TAMs

Next, we performed Kyoto Encyclopedia of Genes and Genomes (KEGG) comparative analysis of *Cxcl9*$^+$*Spp1*$^-$TAMs versus *Cxcl9*$^-$*Spp1*$^+$TAMs, revealing distinct pathway associations: *Cxcl9* was linked to the JAK-STAT signaling pathway, interferon (IFN) signaling, fatty acid metabolism, and the tricarboxylic acid (TCA) cycle, whereas *Spp1* was associated with the PI3K-AKT signaling pathway, hypoxia, angiogenesis, and glycolysis (Figure 5A). The genes related to pathways of TCA and fatty acid metabolism and IFN response were significantly up-regulated in *Cxcl9*$^+$TAMs than *Spp1*$^+$TAMs, whereas the genes associated with glycolysis and PI3K-AKT pathways were up-regulated in *Spp1*$^+$TAMs than *Cxcl9*$^+$TAMs (Figures 5A and S3E–S3G). Consistent with this, *in vivo* TAMs from *Trem2*-KO mice receiving CAR-T therapy exhibited attenuated glycolytic capacity but enhanced TCA cycle and FAO activity (Figure 5B). To validate these findings, we established an *in vitro* tumor-educated macrophage model. Bone marrow-derived macrophages (BMDMs) isolated from *Trem2*-KO or WT mice were educated for 24 h in a transwell system with cancer cells, with or without IFN-γ intervention, since IFN-γ is known to be CAR-T cells' pivotal effector molecule[37] (Figure 5C). RT-qPCR analysis revealed that *Trem2*-KO combined with IFN-γ significantly elevated the *Cxcl9*/*Spp1* ratio (Figure 5D). Furthermore, this combined treatment upregulated the AMP/ATP ratio, the TCA cycle genes (*Idh2* and *Sdha*), and the FAO genes (*Cpt1a* and *Acaa1a*), while downregulating the glycolytic genes (*Ldha* and *Glut1*) (Figures 5E and S4A).

Studies have reported that adenosine monophosphate-activated protein kinase (AMPK) and mammalian target of rapamycin (mTOR) are master regulators of these metabolic pathways.[38,39] Building on the observed metabolic shift, we explored AMPK/mTOR regulation. *Trem2*-KO combined with IFN-γ robustly enhanced AMPK phosphorylation, while suppressing mTOR activation. This was accompanied by increased protein expression of CPT1 (FAO rate-limiting enzyme) and IDH2 (TCA cycle enzyme), with decreased LDHA (glycolytic enzyme)—findings consistent with transcriptional data (Figure 5F). Together, these data suggest a model in which *rem2*-KO synergized with IFN-γ to redirect metabolic flux toward FAO and TCA cycles in a process associated with AMPK-driven mTOR suppression. To dissect AMPK-mTOR crosstalk, we employed pharmacological inhibitors. AMPK inhibition attenuated AMPK phosphorylation, restored mTOR activity, suppressed CPT1/IDH2 expression, and enhanced LDHA (Figure 5G). Conversely, mTOR inhibition potentiated AMPK phosphorylation and further elevated CPT1/IDH2 levels (Figure 5H), supporting the existence of reciprocal regulation in the maintenance of metabolic homeostasis. Mechanistically, as AMPK activation is governed by AMP/ATP ratio,[38] we quantified these metabolites. The mTOR inhibition significantly increased AMP/ATP ratio, while AMPK inhibition reduced it under IFN-γ co-stimulation (Figure S4B), which is consistent with AMPK's role as an energy-sensing effector.

To further validate the functional phenotypes associated with metabolic reprogramming, we designed seahorse experiments based on the aforementioned *in vitro* model. We observed that IFN-γ-treated *Trem2*-KO TAMs showed a robust increase in basal oxygen consumption rate (OCR) and maximal OCR, indicating enhanced oxidative respiration. The addition of etomoxir,[40,41] an inhibitor of FAO, revealed an increased etomoxir-sensitive OCR in *Trem2*-KO TAMs treated with IFN-γ, which suggested enhanced FAO level (Figures 5I and 5J). Meanwhile, IFN-γ-treated *Trem2*-KO TAMs showed a decrease in basal electron consumption rate (ECR), compensatory glycolysis ECR, and eto-sensitive extracellular acidification rate (ECAR), suggesting impaired glycolytic activity (Figures 5K and 5L).

(G) Visualization of tumor-specific TCR clonal expansion classes identified in CD8$^+$ T cell clusters between groups. Four expansion classes were listed.
(H) Bar chart depicting tumor-specific TCR clonotype frequencies across groups. Each bar on the *x* axis represents a TCR clone type. The *y* axis represented the cell number of each TCR. The red dotted line represented the dividing line of large-expansion TCR clone, and the orange dotted line represented the dividing line of medium-expansion TCR clone.
(I) Correlation analysis between expanded cell number of tumor-specific TCRs and anti-tumor score in different groups in tumor (tested using the Spearman correlation test).

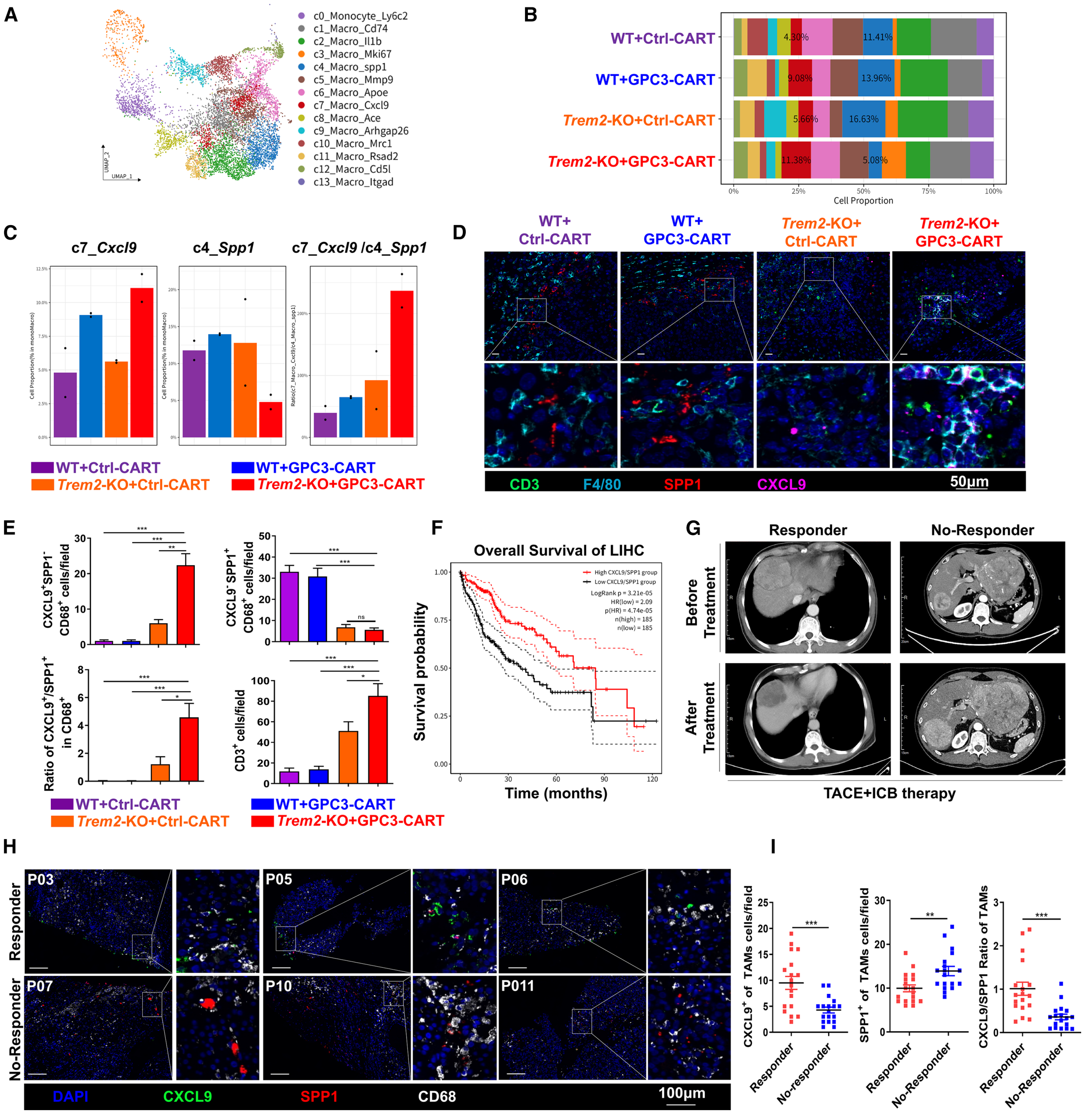

Figure 4. ***Trem2*-KO combined with GPC3-CAR-T-derived TAMs reprogramming toward anti-tumor phenotype**

(A) UMAP of subclusters of TAMs.

(B) Proportion of the distinct TAMs in different groups.

(C) Bar plots revealed the proportion of Macro_*Cxcl9* and Macro_*Spp1* in TAMs and the ratio of Macro_*Cxcl9*/Macro_*Spp1* among different groups.

(D and E) Multiple immunofluorescence images of tumor tissue between groups. Scale bar, 50 μm. Statistical plot represented the relative number of TAM subsets. Quantification of cells numbers from three random fields per mouse (n = 8 mice/group).

(F) Plots of Kaplan-Meier overall survival curves from the TCGA LIHC database, grouped by low and high *CXCL9/SPP1* ratio.

(G) Computed tomography scans of representative patients from responder and no-responder groups were performed before and after TACE combined with anti-PD-1 immunotherapy. The treatment evaluation criteria referred to imRECIST Refining Guidelines.

(H and I) Multiple immunofluorescence images of CXCL9$^+$ and SPP1$^+$ TAMs in punctured tumor tissue between responder and no-responder groups. Scale bars, 100 μm. (Quantification) Data are presented as mean ± SEM. The quantification of cell numbers was performed by analyzing three random low-power fields per patient.

$^*p < 0.05$, $^{**}p < 0.01$, $^{***}p < 0.001$.

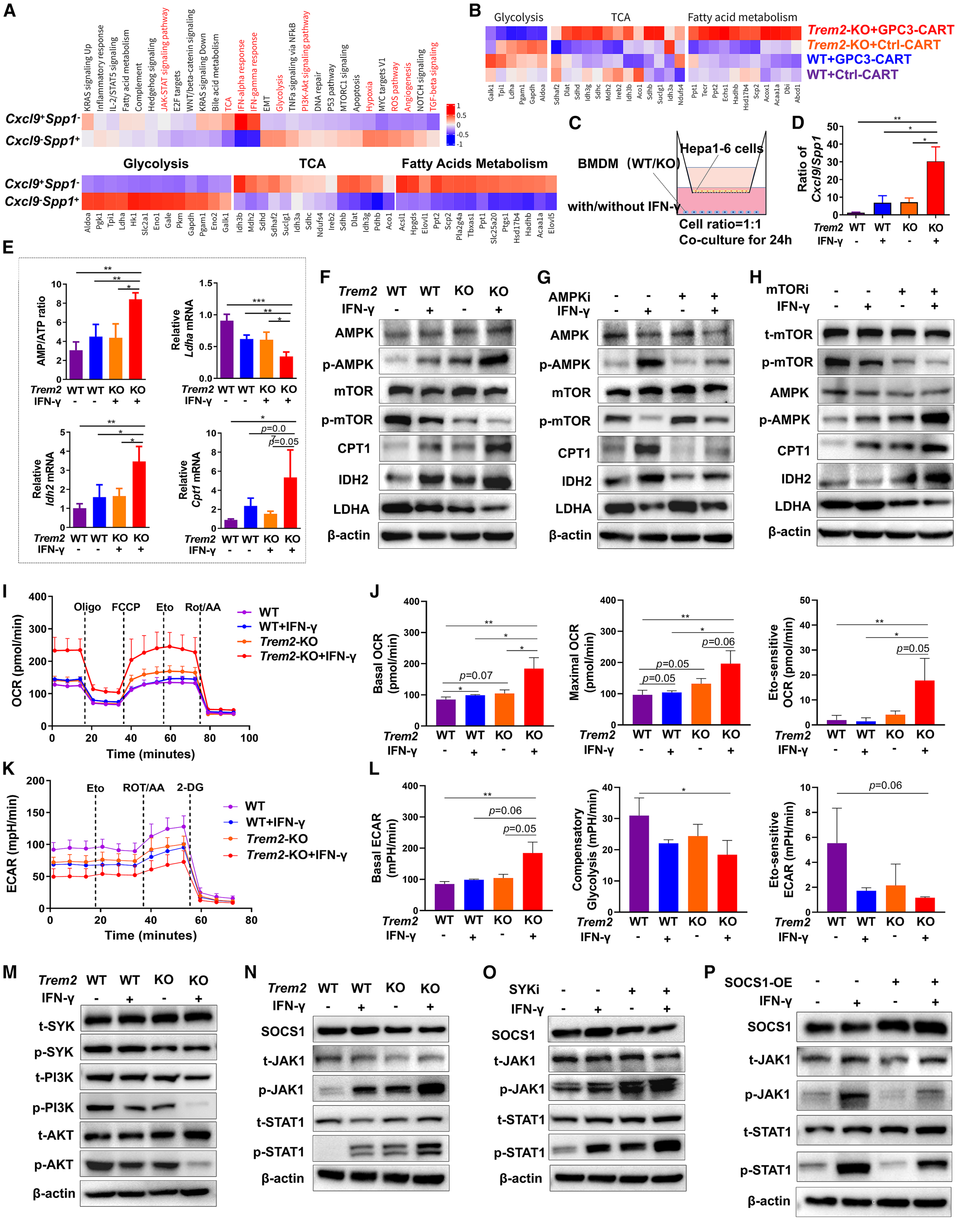

(legend on next page)

Given *Trem2* signals through SYK to activate PI3K-AKT-mTOR,[42] we hypothesized that *Trem2*-KO disrupts this axis. Combined *Trem2*-KO and IFN-γ treatment drastically reduced phosphorylation of SYK, PI3K, and AKT (Figure 5M). Critically, SYK inhibitor treatment elevated *Cxcl9/Spp*1 ratio and phenocopied *Trem2*-KO, suppressing PI3K/AKT phosphorylation (Figures S4C and S4D), indicating that *Trem2*-ablation is associated with disruption of SYK-dependent immunometabolic signaling. Consistent with our prior observation that JAK-STAT signaling regulates CXCL9 expression in Figure 5A, we found that *Trem2*-KO combined with IFN-γ robustly amplified JAK1-STAT1 phosphorylation while suppressing SOCS1 (Figure 5N)—a canonical inhibitor of IFN-γ-driven JAK-STAT activation.[43] Notably, SYK inhibition mirrored these effects, enhancing JAK1-STAT1 activation while reducing SOCS1 levels (Figure 5O), which was consistent with previous studies.[44] To functionally validate SOCS1's role, we over-expressed it in immortalized BMDMs (iBMDMs). SOCS1 overexpression attenuated JAK1-STAT1 phosphorylation and reduced *Cxcl9/Spp1* ratios (Figure 5P), supporting the conclusion that SOCS1 functions as a gatekeeper in IFN-γ-driven polarization.

In summary, our findings support a model whereby *Trem2*-KO synergized with IFN-γ to reprogram TAMs toward an anti-tumor $CXCL9^{hi}/SPP1^{lo}$ phenotype. This reprogramming was accompanied by coordinated suppression of SYK signaling along with enhanced oxidative metabolism. Concurrently we observed potentiated JAK1-STAT1 signaling, SOCS1 downregulation, and AMPK-mediated energy sensing, which collectively correlated with metabolic rewiring away from glycolysis.

CD40 agonism triggers a metabolic reprogram of TAMs similar to that of Trem2-KO

The above results suggested that metabolic reprogramming of TAMs represented a viable strategy to enhance CAR-T therapy efficacy. However, clinical translation of TREM2-targeted agents faces challenges due to potential neurotoxicity risks, given TREM2's predominant expression in the central nervous system (CNS) compared to peripheral tissues.[45] Consequently, we pursued safer TAM reeducation strategies via repurposing clinically approved drugs. We focused on highly expressed genes within *Cxcl9*-positive TAM subpopulation, identifying *Cd40* as significantly enriched in this subset (Figure 6A). Molecular analysis indicated that *Cd40* and *Cxcl9* share key transcriptional regulators including STAT1 (Figure 6B) and exhibit strongly correlated expression patterns (Figure 6C). Critically, *in vitro* experiments showed that *Trem2*-KO TAMs stimulated with IFN-γ exhibited markedly elevated CD40 protein expression (Figure 6D). These findings lend support to our hypothesis that CD40 agonism combined with IFN-γ may effectively reeducate TAMs.

Using the *in vitro* model (Figure 6E), we observed that co-administration of CD40 agonism (FGK45) and IFN-γ synergistically enhanced *Cxcl9* transcription, while suppressing *Spp1* expression, consequently elevating the *Cxcl9/Spp1* ratio (Figure 6F). To assess whether CD40 agonism recapitulates the metabolic reprogramming phenotype of *Trem2*-KO in TAMs, we performed seahorse assays on CD40 agonism-treated macrophages. The IFN-γ plus CD40 agonism group exhibited markedly elevated basal and maximal OCR, demonstrating enhanced oxidative metabolism. Etomoxir treatment revealed increased FAO-dependent OCR (Figures 6G and 6H). Conversely, these cells showed reduced basal ECAR and glycolytic capacity, indicating suppressed glycolysis (Figures 6I and 6J).

Further analysis revealed that CD40 agonism with IFN-γ suppressed mTOR phosphorylation and enhanced AMPK activation, alongside corresponding decreases in LDHA and increases in CPT1 and IDH2 (Figure 6K). This metabolic profile mirrors that seen with Trem2-KO. AMPK inhibitor treatment reversed these CD40-agonism-mediated changes in mTOR, LDHA, CPT1, and IDH2 (Figure 6L) and abrogated the increase in AMP/ATP ratio (Figures 6M and 6N), supporting a role for AMPK in CD40-driven metabolic rewiring, as reported.[28] CD40 agonism plus IFN-γ also enhanced CD40 expression and STAT1 phosphorylation, similar to Trem2-KO (Figure 6O). STAT1 inhibition blocked this activation (Figure 6P), suggesting that both AMPK and STAT1

Figure 5. *Trem2*-KO combined with IFN-γ promotes metabolic reprogramming in TAMs

(A) Heatmap of KEGG pathways and differential gene set analysis of glycolysis, TCA, and fatty acid metabolism. Results were applied separately to $Cxcl9^{+}$ and $Spp1^{+}$ subtypes.

(B) Heatmap of differential gene set analysis of glycolysis, TCA, and fatty acid metabolism. Results were applied separately to each group of CAR-T-treated WT or *Trem2*-KO mice.

(C) Schematic representation of *in vitro* tumor-educated macrophage model. BMDMs isolated from *Trem2*-KO or WT mice were educated for 24 h in a transwell system with cancer cells, with or without IFN-γ (40 ng/mL) intervention.

(D) Relative mRNA expression levels of *Cxcl9* and *Spp1* in BMDMs were quantified by RT-qPCR, and the ratio of *Cxcl9/Spp1* was calculated.

(E) AMP and ATP concentrations in BMDMs were measured using dedicated assay kits, and the AMP/ATP ratio was calculated from these values. Relative mRNA expression levels of Ldha, Idh2, and Cpt1 in BMDMs were quantified by RT-qPCR.

(F) Relative protein expression levels of indicated molecules were assessed by western blot (WB) and normalized to β-actin.

(G, H, and O) BMDMs were pretreated for 2 h with the AMPK inhibitor compound C (20 μM; G), mTOR inhibitor rapamycin (1 μM; H), or SYK inhibitor R406 (1 μM; O), followed by 24 h co-culture with tumor cells in the presence or absence of IFN-γ (40 ng/mL). Protein expression was analyzed by WB and normalized to β-actin.

(I and J) The real-time changes of OCR of BMDMs were stimulated with or without IFN-γ in the basal state and following the additions of oligomycin (Oligo), fluorocarbonyl cyanide phenylhydrazone (FCCP), etomoxir (Eto), and rotenone + antimycin A (Rot/AA). The average of basal OCR, maximal OCR, and Eto-sensitive OCR were revealed.

(K and L) The real-time measurement of ECAR of BMDMs was stimulated with or without IFN-γ in the basal state and following the additions of glucose (Gluc), oligomycin (Oligo) and 2-deoxy-D-glucose (2-DG). The average of basal ECAR, maximal ECAR, and glycolytic reserve were revealed.

(M and N) The relative protein expression levels of indicated molecular were assessed by WB and normalized to β-actin.

(P) The iBMDMs were transiently transfected to overexpress SOCS1 for 48 h, followed by 24-h stimulation with or without IFN-γ (40 ng/mL). Indicated molecular protein expression levels were tested by WB and normalized to β-actin. Data were represented by mean ± SEM. ns, no significance, *$p < 0.05$, **$p < 0.01$, ***$p < 0.001$.

CellPress
OPEN ACCESS

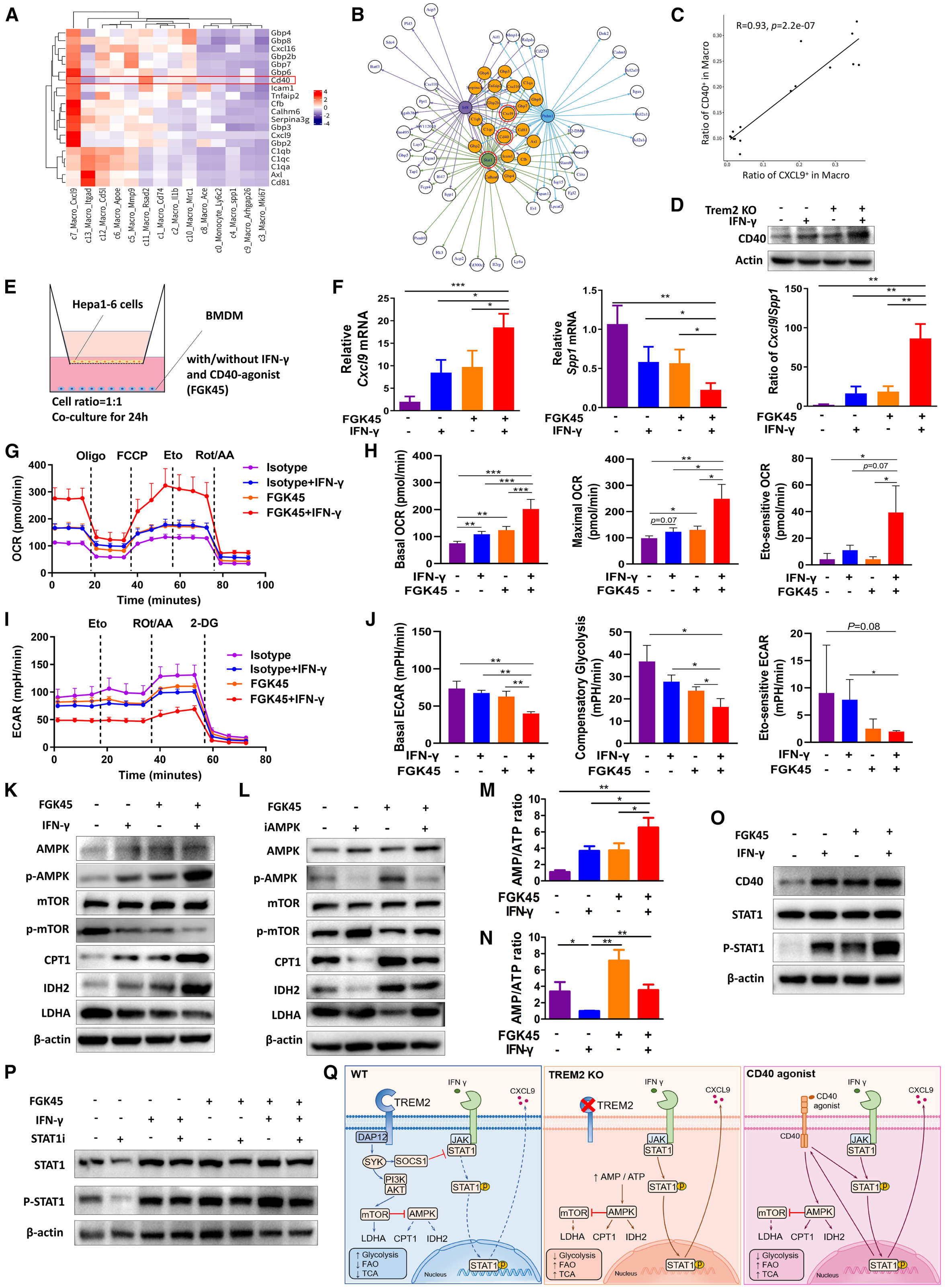

(legend on next page)

contribute to the recapitulation of Trem2-KO-like effects and associated antitumor immunity.

Collectively, we propose a model wherein TREM2 associates with SYK and a PI3K-AKT-mTOR-glycolysis profile, alongside suppressed AMPK and oxidative metabolism. SYK signaling correlates with SOCS1 elevation and reduced JAK-STAT1 activation and CXCL9 production. TREM2 KO disrupts this network, enhancing AMPK activity and, with IFN-γ, the JAK-STAT1-CXCL9 axis. CD40 agonism mirrors this by engaging both AMPK and STAT1, which synergizes with IFN-γ signaling (Figure 6Q).

The combination of CD40 agonism with GPC3-CAR-T therapy held promising application prospects in the treatment of HCC

In vivo testing of CD40 agonism FGK45 combined with GPC3-CAR-T was performed. The tumor model and experimental procedure were shown (Figure 7A). The $FGK45^{+}$GPC3-CAR-T group demonstrated the strongest therapeutic effects (Figures 7B–7D), with superior tumor control compared to other treatment groups. Multiple immunofluorescence assays revealed a decrease in $SPP1^{+}$ TAMs and an increase in $CXCL9^{+}$ TAMs, along with a higher CXCL9/SPP1 ratio in the $FGK45^{+}$GPC3-CAR-T group. Besides, it also demonstrated an elevated numbers of IFN-$\gamma^{+}CD8^{+}$ and IFN-γ^{+} $CD4^{+}$ T cells in the $FGK45^{+}$GPC3-CAR-T group (Figures 7E and 7F). The trend was further confirmed by flow cytometry (Figures 7G and 7H). Notably, neither the number nor the proportion of effector and exhausted CAR-T cells differed significantly across groups (Figure S5A). In the splenic microenvironment, both flow cytometry (Figures S5B and S5C) and immunofluorescence assays (Figures S5D–S5F) showed an increase in the frequency and number of IFN-γ^{+}PD-1^{-} $CD8^{+}$/$CD4^{+}$ T cells in the $FGK45^{+}$GPC3-CAR-T group.

We evaluated the combination of CD40 agonist FGK45 with GPC3–CAR-T cells *in vivo* (Figure 7A). The $FGK45^{+}$GPC3-CAR-T group showed superior tumor control versus other groups (Figures 7B–7D). Multiple immunofluorescence assays revealed a shift in TAM polarization, with decreased $SPP1^{+}$ and increased $CXCL9^{+}$ TAMs and a higher CXCL9/SPP1 ratio in this group, along with elevated numbers of IFN-$\gamma^{+}CD8^{+}$ and IFN-$\gamma^{+}CD4^{+}$ T cells (Figures 7E and 7F). Flow cytometry corroborated these trends (Figures 7G and 7H). Notably, neither CAR-T cell number nor exhaustion proportion differed significantly across groups (Figure S5A). In the splenic microenvironment, flow cytometry and immunofluorescence both indicated increased frequency and number of IFN-γ^{+}PD-$1^{-}CD8^{+}$ and $CD4^{+}$ T cells with combination treatment (Figures S5B–S5F). We further tested this strategy in a breast cancer liver metastasis model using 4T1-hTrop2 cells and $Trop2^{-}$CAR-T cells (Figure 7I). $FGK45^{+}Trop2^{-}$CAR-T resulted in stronger antitumor efficacy (Figures 7K and 7L). Safety assessment showed that combining FGK45 with CAR-T did not increase IL-6 and only modestly elevated IFN-γ, both remaining low compared to a cytokine release syndrome positive control, supporting its favorable safety profile (Figure S6A).

Sotigalimab, a humanized monoclonal antibody, exhibits high-affinity binding to CD40 and effectively activates antigen-presenting cells, being generally well tolerated and achieving path complete response (CR) rates that compare favorably with historical data.[46,47] The transendothelial migration assays revealed that an increased number of migrated $CD8^{+}$ T cells was observed when TAMs were treated with human IFN-γ plus sotigalimab, compared to other groups (Figures 7M and S7A). The maximum migration of $CD8^{+}$ T cells was observed in the group with the digested tumor suspension (DTS) plus CAR-T and sotigalimab (Figures 7N and S7B). These *in vitro* results demonstrated the therapeutic potential of sotigalimab combined with GPC3-CAR-T in HCC, mediated through TAM reeducation to enhance $CD8^{+}$ T cell recruitment.

DISCUSSION

Although GPC3-CAR-T therapy for HCC has shown promising clinical potential, its therapeutic outcomes have not yet met expectations.[4,5,48–50] To enhance the effectiveness of CAR-T cell therapy in solid tumors, it is critical to elucidate the key factors within the TME that influence treatment success. In this study, the conventional GPC3-CAR-T production was used for *in vivo* testing, which was introduced into clinical practice and

Figure 6. CD40 agonism triggers a metabolic reprogram of TAMs similar to that of *Trem2*-KO
(A) Heatmap of differential gene set analysis in TAM subpopulations. Genes were enriched in Mac_Cxcl9 cluster.
(B) The network diagram illustrated the highly expressed genes in Mac_Cxcl9 cluster and their interactions with shared transcription factors.
(C) Correlation analysis between the ratio of *$Cd40^{+}$TAMs* and *$Cxcl9^{+}$TAMs*. Each dot represented a single-cell sequencing sample of tumor and spleen.
(D) BMDMs isolated from *Trem2*-KO or WT mice were educated for 24 h in a transwell system with cancer cells, with or without IFN-γ intervention. Relative protein expression levels of CD40 were assessed by WB and normalized to β-actin.
(E) Schematic representation of *in vitro* tumor-educated macrophage model. BMDMs isolated from WT mice were educated for 24 h in a transwell system with cancer cells, with or without IFN-γ (40 ng/mL) and CD40 agonism (crosslinked FGK45, 20 ng/mL) intervention.
(F) Relative mRNA expression levels of *Cxcl9* and *Spp1* in BMDMs were quantified by RT-qPCR, and the ratio of *Cxcl9/Spp1* was calculated. $n = 3$.
(G and H) The real-time changes of OCR of BMDMs were stimulated with or without IFN-γ and CD40 agonism in the basal state and following the additions of oligomycin (Oligo), FCCP, etomoxir (Eto), and rotenone + antimycin A (Rot/AA). The average of basal OCR, maximal OCR, and Eto-sensitive OCR were revealed. $n = 3$.
(I and J) The real-time measurement of ECAR of BMDMs were stimulated with or without IFN-γ and CD40 agonism in the basal state and following the additions of glucose (Gluc), oligomycin (Oligo), and 2-deoxy-D-glucose (2-DG). The average of basal ECAR, maximal ECAR, and glycolytic reserve were revealed. $n = 3$.
(K, L, O, and P) BMDMs from WT mice were co-cultured with tumor cells in transwells for 24 h with or without IFN-γ (40 ng/mL) and CD40 agonism (crosslinked FGK45, 20 ng/mL) (K and O). For inhibitor studies, BMDMs were pre-treated with AMPK inhibitor (20 μM) (L) or STAT1 inhibitor (100 μM) (P) for 2 h prior to co-culture. Protein levels were analyzed by western blot and normalized to β-actin.
(M and N) AMP and ATP concentrations in BMDMs were measured using dedicated assay kits, and the AMP/ATP ratio was calculated from these values. $n = 3$.
(Q) Mechanism schema diagram. Data were represented by mean ± SEM. ns, no significance, $^{*}p < 0.05$, $^{**}p < 0.01$, $^{***}p < 0.001$.

CellPress
OPEN ACCESS

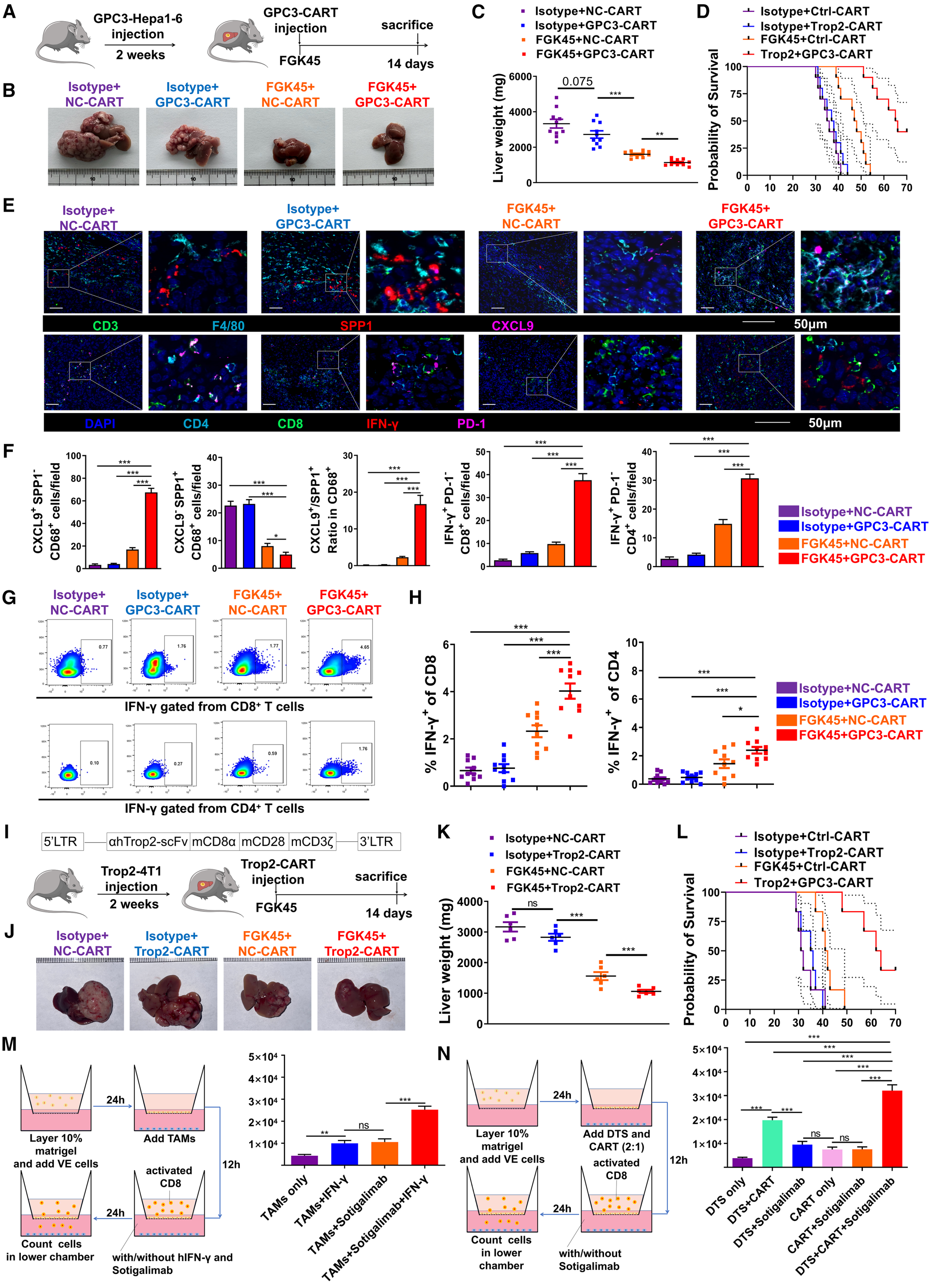

(legend on next page)

demonstrated an excellent safety profile.[11,12] Hepa1-6 cells expressing hGPC3 were combined with hGPC3-targeted murine CAR-T cells to create an immunocompetent gene-KO model. This system allowed the study of TME dynamics under combined CAR-T and genetic modification pressures, offering advantages over immune KO models for assessing intact immune responses. In this study, GPC3-CAR-T therapy showed reduced efficacy against orthotopic versus subcutaneous HCC, due to differences in TME composition. Orthotopic tumors had more TAMs, particularly *Trem2*$^{+}$ subsets, creating a stronger immunosuppressive environment. GPC3-CAR-T alone showed limited efficacy against the “cold” Hepa1-6 orthotopic HCC, unlike the more responsive MC38 model.[51,52] Combining *Trem2*-KO with GPC3-CAR-T cells better controlled orthotopic HCC progression than monotherapies and reshaped the TME immune landscape. The combination therapy effectively remodeled the TME, characterized by increased CD8_Trm, CD8_Tem, CD8_Te, and Th1_effector populations. Notably, *Trem2*-KO plus CAR-T therapy expanded cytotoxic, less-exhausted CD8_Tem, CD8_Te, and Th1_effector subsets in spleen.

CAR-T cells alone showed no enhancement in infiltration or function when combined with other therapies. Notably, the combination therapy increased effector and memory TCR clones in both the TME and spleen, suggesting accelerated renewal of tumor-reactive T cells. These cells likely originated from infiltrating peripheral effectors and expanded in the spleen, resembling stem-like effector memory T cells found in tumor-draining lymph nodes—a subset known to robustly respond to PD-1/PD-L1 blockade.[53,54] While this spleen-mediated expansion is atypical, it may be attributed to the liver tumor’s proximity to the spleen.[55,56]

Accumulating data highlighted the profound effect of TAM reprogramming on both tumor prognosis and therapeutic response to immunotherapies.[57,58] However, the conventional M1/M2 polarization has proven inadequate for characterizing TAMs’ heterogeneity and function.[59] Recent studies have demonstrated a high CXCL9/SPP1 ratio in TAMs correlated with improved clinical outcomes, and it served as a functional marker for antitumor activity.[36] Our data demonstrated that *Trem2*-KO combined with CAR-T therapy markedly elevated the CXCL9/SPP1 ratio, suggesting CAR-T-derived IFN-γ may drive *Trem2*-KO TAMs toward an antitumor phenotype. The TREM2 ligation-induced SYK phosphorylation, followed by activation of the PI3K-AKT-mTOR pathway, was consistent with their involvement in hypoxia response, angiogenic promotion, and glycolytic reprogramming,[20,60] which was resulting in protumorous cellular activation.[42,61] Meanwhile, SYK can suppress JAK1/STAT1 phosphorylation via SOCS1, thereby inhibiting CXCL9 transcription.[44,62] The TREM2/SYK-driven mTOR activation inhibited AMPK phosphorylation, leading to compromised oxidative metabolism in TAMs.[38,39,42,63]

The anti-tumorous CXCL9^{+} TAMs derived from IFN-γ stimulation on *Trem2*-KO TAMs were mainly powered by oxidative metabolism and IFNgR/JAK1/STAT1 pathway. Currently, TREM2-targeting antibodies or inhibitors remain unavailable in clinical trials, primarily due to potential CNS-related adverse effects.[45] This concern arises from the substantially higher expression levels of TREM2 in the CNS compared to other tissues.[64] Consequently, we identified CD40 as a marker strongly correlated with CXCL9 expression and hypothesized that CD40 agonism may promote the differentiation of TAMs into an anti-tumorigenic phenotype. Notably, sotigalimab has been verified safe and can achieve path CR rates that compare favorably with historical data.[46,47]

CD40 agonism mediated signaling-derived pro-inflammatory and anti-tumorigenic polarization in TAMs.[28] Consistent with prior findings, our study demonstrated that CD40 agonism combined with IFN-γ shifted TAMs toward an anti-tumorigenic phenotype by elevating the CXCL9/SPP1 ratio via enhanced oxidative metabolism. This mechanism contrasted with the classical lipopolysaccharide (LPS)-driven M1 polarization of infectious macrophage model, which depends on increased glycolysis and TCA cycle disruption.[65,66] Notably, while LPS-mediated M1 polarization inactivated AMPK,[66] CD40 signaling was shown to induce AMPK phosphorylation at Thr172, thereby promoting macrophage survival.[67] Importantly, both CD40 agonism plus IFN-γ and *Trem2*-KO combined with IFN-γ enhance AMPK phosphorylation at Thr172—a key indicator of oxidative metabolism and improved mitochondrial function.[68] In the

Figure 7. The combination of CD40 agonism with GPC3-CAR-T therapy held promising application prospects in the treatment of HCC

(A) Schematic procedure of HCC model constructed by Hepa1-6 cell orthotopic injection. GPC3-CART and CD40 agonist (FGK45) were injected.

(B) Tumor representative photographs of different groups were shown.

(C) Dot plots showed liver weight of mice in different groups. $n = 8$.

(D) Survival curves of each group of mice. $n = 8$.

(E and F) Multiple immunofluorescence images of TAMs, CD8^{+}, and CD4^{+} T cells in tumor tissue between groups. Scale bar, 50 μm. Quantification of cells numbers from three random fields per mouse ($n = 8$ mice/group). Data were represented by mean ± SEM. ***$p < 0.001$.

(G and H) Flow cytometry and quantification plots of IFN-γ^{+}CD8^{+} T cells and IFN-γ^{+}CD4^{+} T cells, as well as IFN-γ^{-}CD8^{+} T cells and IFN-γ^{-}CD4^{+} T cells from TILs *in vivo*. $N = 8$. Data were represented by mean ± SEM. *$p < 0.01$, ***$p < 0.001$.

(I) Schematic procedure of a therapeutic breast cancer liver metastasis model constructed by 4T1 cell expressing hTrop2 protein. Murine hTrop2-CAR-T and CD40 agonist (FGK45) were injected.

(J) Tumor representative photographs of different groups were shown.

(K) Dot plots showed liver weight of mice in different groups. $n = 8$. Data were represented by mean ± SEM. ns, no significance, ***$p < 0.001$.

(L) Survival curves of each group of mice. $n = 8$.

(M) Schematic representation of the transendothelial migration assay. TAMs were isolated from human HCC tissue. Sotigalimab (20 ng/mL) and h-IFN-γ (40 ng/mL) were added. Flow cytometry quantification of migrated CD8^{+} T cells (CellTrace Far Red labeled, $n = 5$). Data were represented by mean ± SEM. ns, no significance, **$p < 0.01$, ***$p < 0.001$.

(N) Schematic representation of the transendothelial migration assay. DTS was isolated from human GPC3^{+} HCC tissue. GPC3-CAR-T was added at indicated ratio, with or without sotigalimab. Flow cytometry quantification of migrated CD8^{+} T cells (CellTrace Far Red labeled, $n = 5$). Data were represented by mean ± SEM. ns, no significance, ***$p < 0.001$.

clinical application prospect, sotigalimab plus CAR-T therapy showed strong potential to enhance T cell infiltration *in vitro*.

The combination of CD40 agonism with GPC3-CAR-T for HCC treatment offered several advantages as below. GPC3-CAR-T directly eliminates $GPC3^+$ HCC cells, releasing neoantigens to initiate subsequent adaptive immune responses. The CD40 agonism and IFN-γ secreted by CAR-T promoted TAM reeducation toward an anti-tumor phenotype, with CXCL9 secretion recruiting endogenous $CD8^+$ T cells to infiltrate tumors, establishing an endogenous immune response. While CAR-T served as the triggering mechanism, the endogenous adaptive immune response became the main anti-tumor force. Compared to CAR-T monotherapy that relied solely on CAR-T persistence, endogenous $CD8^+$ T cells exhibit broader antigen recognition and can eliminate GPC3-negative tumor cells, reducing immune escape. Given that HCC was generally resistant to radiotherapy and chemotherapy, and lacks effective neoadjuvant options commonly used in other cancers,[3] this approach may represent a promising neoadjuvant strategy, particularly for "immune-cold" HCC.

In conclusion, combining CD40 agonism or *Trem2*-KO with CAR-T therapy may reeducate TAMs toward an anti-tumorigenic phenotype, promoting tumor-reactive T cell recruitment, although CAR-T infiltration and function in solid tumors were not enhanced. This suggests an alternative strategy to improve CAR-T efficacy: rather than solely modifying CAR-T cells, targeting TAMs offers a promising approach to boost adaptive anti-tumor immunity.

Limitations of the study

This study is limited by its reliance on *in vitro* data using only sotigalimab, which does not fully capture the *in vivo* TME or enable comparison with other CD40 agonists. To address this, we will utilize humanized NSG-SGM3 mice reconstituted with human $CD34^+$ cells and orthotopic HCC models to evaluate both sotigalimab and selicrelumab in combination with GPC3-CAR-T cells. This will allow us to optimize dosing, assess safety and efficacy, and validate the therapeutic potential of this combination approach for clinical translation. Further experiments are warranted to evaluate the hypothesis in the future.

RESOURCE AVAILABILITY

Lead contact

Requests for further information and resources should be directed to and will be fulfilled by the lead contact, Jizhou Tan (tanjzh@mail.sysu.edu.cn).

Materials availability

All unique/stable reagents generated in this study will be made available on request, but we may require payment and/or a completed materials transfer agreement if there is potential for commercial application.

Data and code availability

- The long-term outcome data of GPC3-CAR-T-treated patients were obtained from the published article in *Nature* (2025) by Steffin et al. (https://doi.org/10.1038/s41586-024-08261-8). The single-cell RNA-seq data of patients were obtained from the Gene Expression Omnibus (no. GSE253352). The raw fastqs generated in this study are available on the public NCBI Database of Sequence Read Archive (SRA) website with the accession number PRJNA1303879 (https://www.ncbi.nlm.nih.gov/bioproject/PRJNA1303879/).
- This paper does not report original code.
- Any additional information required to reanalyze the data reported in this work paper is available from the lead contact upon request.

ACKNOWLEDGMENTS

This work was supported by grants from National Natural Science Foundation of China (82202986, 82574817, and 82472080), the Natural Science Foundation of Guangdong Province (2023A1515220017, 2024A1515012867, 2025A1515010135, and 2023A1515010481), Young Doctor "Sailing" project of Science and Technology Department of Guangzhou (2024A04J3291), Outstanding Young Talents Seedling Program of Guangdong Hospital of Traditional Chinese Medicine (SZ2023QN03), Youth Science and Technology Innovation Talent of Guangdong TeZhi plan (0720240275), Scientific research project of Guangdong provincial administration of Traditional Chinese Medicine (20251147), and the national nature cultivation project of Guangdong Hospital of Traditional Chinese Medicine (YN2024GZRPY061).

AUTHOR CONTRIBUTIONS

J.T., T.L., H.G., and Z.X. wrote the manuscript. J.T. and T.L. designed the experiments. T.L., H.G., Z.X., T.Y., Y.G., H.M., H.Y., H.L., and Q.Z. performed experiments and analyzed data. X.H. participated in article revision, W.F. provided clinical samples and data, and J.T., T.L., and W.F. supervised the project.

DECLARATION OF INTERESTS

The authors have declared that no conflict of interest exists.

STAR★METHODS

Detailed methods are provided in the online version of this paper and include the following:

- KEY RESOURCES TABLE
- EXPERIMENTAL MODEL AND STUDY PARTICIPANT DETAILS
  - Mice and *in vivo* studies
  - Cell lines
  - Human tissue samples
- METHOD DETAILS
  - CART generation
  - Single cell suspension preparation for tumor of mice
  - Sorting for live $CD45^+$ cells
  - Single cell library construction
  - Single cell RNAseq (scRNAseq) preprocess
  - Detection of CAR-T insertion and *Trem2* deletion in cells
  - Identification of cell clusters
  - Differential expressed gene analysis
  - Single cell TCRseq (scTCRseq) data analysis
  - Cell development trajectory for $CD8^+$ T cells
  - Definition of cell scores and signatures
  - Pathway analysis
  - Survival analysis and validation on TCGA
  - Reverse transcription Quantitative PCR (RT-QPCR)
  - Multiple immunofluorescence staining
  - Flow cytometry
  - Bone marrow-derived macrophages (BMDMs) isolation and culture
  - Plasmid construction and cell transfection
  - $CD8^+$ T cell transendothelial migration assay
  - *In vitro* tumor-educated macrophage model
  - Intracellular ATP and AMP measurement
  - Western blot
  - Seahorse XF Cell Mito stress assay and seahorse XF Cell glycolysis rate assay
- QUANTIFICATION AND STATISTICAL ANALYSIS

SUPPLEMENTAL INFORMATION

Supplemental information can be found online at https://doi.org/10.1016/j.xcrm.2025.102539.

Received: January 7, 2025
Revised: September 17, 2025
Accepted: November 28, 2025
Published: January 20, 2026

REFERENCES

1. Sung, H., Ferlay, J., Siegel, R.L., Laversanne, M., Soerjomataram, I., Jemal, A., and Bray, F. (2021). Global Cancer Statistics 2020: GLOBOCAN Estimates of Incidence and Mortality Worldwide for 36 Cancers in 185 Countries. CA Cancer J. Clin. *71*, 209–249. https://doi.org/10.3322/caac.21660.
2. Zhang, Z.Z., Wang, T., Wang, X.F., Zhang, Y.Q., Song, S.X., and Ma, C.Q. (2022). Improving the ability of CAR-T cells to hit solid tumors: Challenges and strategies. Pharmacol. Res. *175*, 106036. https://doi.org/10.1016/j.phrs.2021.106036.
3. Llovet, J.M., Castet, F., Heikenwalder, M., Maini, M.K., Mazzaferro, V., Pinato, D.J., Pikarsky, E., Zhu, A.X., and Finn, R.S. (2022). Immunotherapies for hepatocellular carcinoma. Nat. Rev. Clin. Oncol. *19*, 151–172. https://doi.org/10.1038/s41571-021-00573-2.
4. Zhou, F., Shang, W., Yu, X., and Tian, J. (2018). Glypican-3: A promising biomarker for hepatocellular carcinoma diagnosis and treatment. Med. Res. Rev. *38*, 741–767. https://doi.org/10.1002/med.21455.
5. Maalej, K.M., Merhi, M., Inchakalody, V.P., Mestiri, S., Alam, M., Maccalli, C., Cherif, H., Uddin, S., Steinhoff, M., Marincola, F.M., and Dermime, S. (2023). CAR-cell therapy in the era of solid tumor treatment: current challenges and emerging therapeutic advances. Mol. Cancer *22*, 20. https://doi.org/10.1186/s12943-023-01723-z.
6. Yong, C.S.M., Dardalhon, V., Devaud, C., Taylor, N., Darcy, P.K., and Kershaw, M.H. (2017). CAR T-cell therapy of solid tumors. Immunol. Cell Biol. *95*, 356–363. https://doi.org/10.1038/icb.2016.128.
7. Batra, S.A., Rathi, P., Guo, L., Courtney, A.N., Fleurence, J., Balzeau, J., Shaik, R.S., Nguyen, T.P., Wu, M.F., Bulsara, S., et al. (2020). Glypican-3-Specific CAR T Cells Coexpressing IL15 and IL21 Have Superior Expansion and Antitumor Activity against Hepatocellular Carcinoma. Cancer Immunol. Res. *8*, 309–320. https://doi.org/10.1158/2326-6066.CIR-19-0293.
8. Steffin, D., Ghatwai, N., Montalbano, A., Rathi, P., Courtney, A.N., Arnett, A.B., Fleurence, J., Sweidan, R., Wang, T., Zhang, H., et al. (2025). Interleukin-15-armoured GPC3 CAR T cells for patients with solid cancers. Nature *637*, 940–946. https://doi.org/10.1038/s41586-024-08261-8.
9. Weber, E.W., Parker, K.R., Sotillo, E., Lynn, R.C., Anbunathan, H., Lattin, J., Good, Z., Belk, J.A., Daniel, B., Klysz, D., et al. (2021). Transient rest restores functionality in exhausted CAR-T cells through epigenetic remodeling. Science *372*, eaba1786. https://doi.org/10.1126/science.aba1786.
10. Chen, R., Chen, L., Wang, C., Zhu, H., Gu, L., Li, Y., Xiong, X., Chen, G., and Jian, Z. (2023). CAR-T treatment for cancer: prospects and challenges. Front. Oncol. *13*, 1288383. https://doi.org/10.3389/fonc.2023.1288383.
11. Zheng, R., Zhu, X., and Xiao, Y. (2024). Advances in CAR-T-cell therapy in T-cell malignancies. J. Hematol. Oncol. *17*, 49. https://doi.org/10.1186/s13045-024-01568-z.
12. Baybutt, T.R., Flickinger, J.C., Jr., Caparosa, E.M., and Snook, A.E. (2019). Advances in Chimeric Antigen Receptor T-Cell Therapies for Solid Tumors. Clin. Pharmacol. Ther. *105*, 71–78. https://doi.org/10.1002/cpt.1280.
13. Wang, H., Shao, Q., Wang, J., Zhao, L., Wang, L., Cheng, Z., Yue, C., Chen, W., Wang, H., and Zhang, Y. (2022). Decreased CXCR2 expression on circulating monocytes of colorectal cancer impairs recruitment and induces Re-education of tumor-associated macrophages. Cancer Lett. *529*, 112–125. https://doi.org/10.1016/j.canlet.2022.01.004.
14. van Elsas, M.J., Middelburg, J., Labrie, C., Roelands, J., Schaap, G., Sluijter, M., Tonea, R., Ovcinnikovs, V., Lloyd, K., Schuurman, J., et al. (2024). Immunotherapy-activated T cells recruit and skew late-stage activated M1-like macrophages that are critical for therapeutic efficacy. Cancer Cell *42*, 1032–1050.e10. https://doi.org/10.1016/j.ccell.2024.04.011.
15. Yofe, I., Shami, T., Cohen, N., Landsberger, T., Sheban, F., Stoler-Barak, L., Yalin, A., Phan, T.S., Li, B., Monteran, L., et al. (2023). Spatial and Temporal Mapping of Breast Cancer Lung Metastases Identify TREM2 Macrophages as Regulators of the Metastatic Boundary. Cancer Discov. *13*, 2610–2631. https://doi.org/10.1158/2159-8290.CD-23-0299.
16. Park, M.D., Reyes-Torres, I., LeBerichel, J., Hamon, P., LaMarche, N.M., Hegde, S., Belabed, M., Troncoso, L., Grout, J.A., Magen, A., et al. (2023). TREM2 macrophages drive NK cell paucity and dysfunction in lung cancer. Nat. Immunol. *24*, 792–801. https://doi.org/10.1038/s41590-023-01475-4.
17. Yu, M., Chang, Y., Zhai, Y., Pang, B., Wang, P., Li, G., Jiang, T., and Zeng, F. (2022). TREM2 is associated with tumor immunity and implies poor prognosis in glioma. Front. Immunol. *13*, 1089266. https://doi.org/10.3389/fimmu.2022.1089266.
18. Binnewies, M., Pollack, J.L., Rudolph, J., Dash, S., Abushawish, M., Lee, T., Jahchan, N.S., Canaday, P., Lu, E., Norng, M., et al. (2021). Targeting TREM2 on tumor-associated macrophages enhances immunotherapy. Cell Rep. *37*, 109844. https://doi.org/10.1016/j.celrep.2021.109844.
19. Molgora, M., Esaulova, E., Vermi, W., Hou, J., Chen, Y., Luo, J., Brioschi, S., Bugatti, M., Omodei, A.S., Ricci, B., et al. (2020). TREM2 Modulation Remodels the Tumor Myeloid Landscape Enhancing Anti-PD-1 Immunotherapy. Cell *182*, 886–900.e17. https://doi.org/10.1016/j.cell.2020.07.013.
20. Tan, J., Fan, W., Liu, T., Zhu, B., Liu, Y., Wang, S., Wu, J., Liu, J., Zou, F., Wei, J., et al. (2023). TREM2(+) macrophages suppress CD8(+) T-cell infiltration after transarterial chemoembolisation in hepatocellular carcinoma. J. Hepatol. *79*, 126–140. https://doi.org/10.1016/j.jhep.2023.02.032.
21. Reifschneider, A., Robinson, S., van Lengerich, B., Gnörich, J., Logan, T., Heindl, S., Vogt, M.A., Weidinger, E., Riedl, L., Wind, K., et al. (2022). Loss of TREM2 rescues hyperactivation of microglia, but not lysosomal deficits and neurotoxicity in models of progranulin deficiency. EMBO J. *41*, e109108. https://doi.org/10.15252/embj.2021109108.
22. Deczkowska, A., Weiner, A., and Amit, I. (2020). The Physiology, Pathology, and Potential Therapeutic Applications of the TREM2 Signaling Pathway. Cell *181*, 1207–1217. https://doi.org/10.1016/j.cell.2020.05.003.
23. Jonsson, T., Stefansson, H., Steinberg, S., Jonsdottir, I., Jonsson, P.V., Snaedal, J., Bjornsson, S., Huttenlocher, J., Levey, A.I., Lah, J.J., et al. (2013). Variant of TREM2 associated with the risk of Alzheimer's disease. N. Engl. J. Med. *368*, 107–116. https://doi.org/10.1056/NEJMoa1211103.
24. Guerreiro, R., Wojtas, A., Bras, J., Carrasquillo, M., Rogaeva, E., Majounie, E., Cruchaga, C., Sassi, C., Kauwe, J.S.K., Younkin, S., et al. (2013). TREM2 variants in Alzheimer's disease. N. Engl. J. Med. *368*, 117–127. https://doi.org/10.1056/NEJMoa1211851.
25. Beatty, G.L., Chiorean, E.G., Fishman, M.P., Saboury, B., Teitelbaum, U.R., Sun, W., Huhn, R.D., Song, W., Li, D., Sharp, L.L., et al. (2011). CD40 agonists alter tumor stroma and show efficacy against pancreatic carcinoma in mice and humans. Science *331*, 1612–1616. https://doi.org/10.1126/science.1198443.
26. Ho, P.C., Meeth, K.M., Tsui, Y.C., Srivastava, B., Bosenberg, M.W., and Kaech, S.M. (2014). Immune-based antitumor effects of BRAF inhibitors rely on signaling by CD40L and IFNgamma. Cancer Res. *74*, 3205–3217. https://doi.org/10.1158/0008-5472.CAN-13-3461.
27. Kashyap, A.S., Schmittnaegel, M., Rigamonti, N., Pais-Ferreira, D., Mueller, P., Buchi, M., Ooi, C.H., Kreuzaler, M., Hirschmann, P., Guichard, A., et al. (2020). Optimized antiangiogenic reprogramming of the tumor

microenvironment potentiates CD40 immunotherapy. Proc. Natl. Acad. Sci. USA *117*, 541–551. https://doi.org/10.1073/pnas.1902145116.

28. Liu, P.S., Chen, Y.T., Li, X., Hsueh, P.C., Tzeng, S.F., Chen, H., Shi, P.Z., Xie, X., Parik, S., Planque, M., et al. (2023). CD40 signal rewires fatty acid and glutamine metabolism for stimulating macrophage anti-tumorigenic functions. Nat. Immunol. *24*, 452–462. https://doi.org/10.1038/s41590-023-01430-3.
29. Oliveira, G., Stromhaug, K., Klaeger, S., Kula, T., Frederick, D.T., Le, P.M., Forman, J., Huang, T., Li, S., Zhang, W., et al. (2021). Phenotype, specificity and avidity of antitumour CD8(+) T cells in melanoma. Nature *596*, 119–125. https://doi.org/10.1038/s41586-021-03704-y.
30. Oliveira, G., Egloff, A.M., Afeyan, A.B., Wolff, J.O., Zeng, Z., Chernock, R.D., Zhou, L., Messier, C., Lizotte, P., Pfaff, K.L., et al. (2023). Preexisting tumor-resident T cells with cytotoxic potential associate with response to neoadjuvant anti-PD-1 in head and neck cancer. Sci. Immunol. *8*, eadf4968. https://doi.org/10.1126/sciimmunol.adf4968.
31. Oliveira, G., and Wu, C.J. (2023). Dynamics and specificities of T cells in cancer immunotherapy. Nat. Rev. Cancer *23*, 295–316. https://doi.org/10.1038/s41568-023-00560-y.
32. Lowery, F.J., Krishna, S., Yossef, R., Parikh, N.B., Chatani, P.D., Zacharakis, N., Parkhurst, M.R., Levin, N., Sindiri, S., Sachs, A., et al. (2022). Molecular signatures of antitumor neoantigen-reactive T cells from metastatic human cancers. Science *375*, 877–884. https://doi.org/10.1126/science.abl5447.
33. Subramanian, A., Tamayo, P., Mootha, V.K., Mukherjee, S., Ebert, B.L., Gillette, M.A., Paulovich, A., Pomeroy, S.L., Golub, T.R., Lander, E.S., and Mesirov, J.P. (2005). Gene set enrichment analysis: a knowledge-based approach for interpreting genome-wide expression profiles. Proc. Natl. Acad. Sci. USA *102*, 15545–15550. https://doi.org/10.1073/pnas.0506580102.
34. Tooley, K., Jerby, L., Escobar, G., Krovi, S.H., Mangani, D., Dandekar, G., Cheng, H., Madi, A., Goldschmidt, E., Lambden, C., et al. (2024). Pan-cancer mapping of single CD8(+) T cell profiles reveals a TCF1:CXCR6 axis regulating CD28 co-stimulation and anti-tumor immunity. Cell Rep. Med. *5*, 101640. https://doi.org/10.1016/j.xcrm.2024.101640.
35. Mackay, L.K., Rahimpour, A., Ma, J.Z., Collins, N., Stock, A.T., Hafon, M.L., Vega-Ramos, J., Lauzurica, P., Mueller, S.N., Stefanovic, T., et al. (2013). The developmental pathway for CD103(+)CD8+ tissue-resident memory T cells of skin. Nat. Immunol. *14*, 1294–1301. https://doi.org/10.1038/ni.2744.
36. Bill, R., Wirapati, P., Messemaker, M., Roh, W., Zitti, B., Duval, F., Kiss, M., Park, J.C., Saal, T.M., Hoelzl, J., et al. (2023). CXCL9:SPP1 macrophage polarity identifies a network of cellular programs that control human cancers. Science *381*, 515–524. https://doi.org/10.1126/science.ade2292.
37. Zou, F., Tan, J., Liu, T., Liu, B., Tang, Y., Zhang, H., and Li, J. (2021). The CD39(+) HBV surface protein-targeted CAR-T and personalized tumor-reactive CD8(+) T cells exhibit potent anti-HCC activity. Mol. Ther. *29*, 1794–1807. https://doi.org/10.1016/j.ymthe.2021.01.021.
38. Steinberg, G.R., and Hardie, D.G. (2023). New insights into activation and function of the AMPK. Nat. Rev. Mol. Cell Biol. *24*, 255–272. https://doi.org/10.1038/s41580-022-00547-x.
39. Mossmann, D., Park, S., and Hall, M.N. (2018). mTOR signalling and cellular metabolism are mutual determinants in cancer. Nat. Rev. Cancer *18*, 744–757. https://doi.org/10.1038/s41568-018-0074-8.
40. Huang, S.C.C., Everts, B., Ivanova, Y., O'Sullivan, D., Nascimento, M., Smith, A.M., Beatty, W., Love-Gregory, L., Lam, W.Y., O'Neill, C.M., et al. (2014). Cell-intrinsic lysosomal lipolysis is essential for alternative activation of macrophages. Nat. Immunol. *15*, 846–855. https://doi.org/10.1038/ni.2956.
41. van der Windt, G.J.W., Everts, B., Chang, C.H., Curtis, J.D., Freitas, T.C., Amiel, E., Pearce, E.J., and Pearce, E.L. (2012). Mitochondrial respiratory capacity is a critical regulator of CD8+ T cell memory development. Immunity *36*, 68–78. https://doi.org/10.1016/j.immuni.2011.12.007.
42. Wang, S., Sudan, R., Peng, V., Zhou, Y., Du, S., Yuede, C.M., Lei, T., Hou, J., Cai, Z., Cella, M., et al. (2022). TREM2 drives microglia response to amyloid-beta via SYK-dependent and -independent pathways. Cell *185*, 4153–4169.e19. https://doi.org/10.1016/j.cell.2022.09.033.
43. Sk, M.F., Jonniya, N.A., Roy, R., and Kar, P. (2022). Phosphorylation-Induced Conformational Dynamics and Inhibition of Janus Kinase 1 by Suppressors of Cytokine Signaling 1. J. Phys. Chem. B *126*, 3224–3239. https://doi.org/10.1021/acs.jpcb.1c10733.
44. Eberle, M.E., and Dalpke, A.H. (2012). Dectin-1 stimulation induces suppressor of cytokine signaling 1, thereby modulating TLR signaling and T cell responses. J. Immunol. *188*, 5644–5654. https://doi.org/10.4049/jimmunol.1103068.
45. Nugent, A.A., Lin, K., van Lengerich, B., Lianoglou, S., Przybyla, L., Davis, S.S., Llapashtica, C., Wang, J., Kim, D.J., Xia, D., et al. (2020). TREM2 Regulates Microglial Cholesterol Metabolism upon Chronic Phagocytic Challenge. Neuron *105*, 837–854.e9. https://doi.org/10.1016/j.neuron.2019.12.007.
46. O'Hara, M.H., O'Reilly, E.M., Varadhachary, G., Wolff, R.A., Wainberg, Z.A., Ko, A.H., Fisher, G., Rahma, O., Lyman, J.P., Cabanski, C.R., et al. (2021). CD40 agonistic monoclonal antibody APX005M (sotigalimab) and chemotherapy, with or without nivolumab, for the treatment of metastatic pancreatic adenocarcinoma: an open-label, multicentre, phase 1b study. Lancet Oncol. *22*, 118–131. https://doi.org/10.1016/S1470-2045(20)30532-5.
47. Padron, L.J., Maurer, D.M., O'Hara, M.H., O'Reilly, E.M., Wolff, R.A., Wainberg, Z.A., Ko, A.H., Fisher, G., Rahma, O., Lyman, J.P., et al. (2022). Sotigalimab and/or nivolumab with chemotherapy in first-line metastatic pancreatic cancer: clinical and immunologic analyses from the randomized phase 2 PRINCE trial. Nat Med *28*, 1167–1177. https://doi.org/10.1038/s41591-022-01829-9.
48. Taniguchi, M., Mizuno, S., Yoshikawa, T., Fujinami, N., Sugimoto, M., Kobayashi, S., Takahashi, S., Konishi, M., Gotohda, N., and Nakatsura, T. (2020). Peptide vaccine as an adjuvant therapy for glypican-3-positive hepatocellular carcinoma induces peptide-specific CTLs and improves long prognosis. Cancer Sci. *111*, 2747–2759. https://doi.org/10.1111/cas.14497.
49. Sawada, Y., Yoshikawa, T., Nobuoka, D., Shirakawa, H., Kuronuma, T., Motomura, Y., Mizuno, S., Ishii, H., Nakachi, K., Konishi, M., et al. (2012). Phase I trial of a glypican-3-derived peptide vaccine for advanced hepatocellular carcinoma: immunologic evidence and potential for improving overall survival. Clin. Cancer Res. *18*, 3686–3696. https://doi.org/10.1158/1078-0432.CCR-11-3044.
50. Pang, N., Shi, J., Qin, L., Chen, A., Tang, Y., Yang, H., Huang, Y., Wu, Q., Li, X., He, B., et al. (2021). IL-7 and CCL19-secreting CAR-T cell therapy for tumors with positive glypican-3 or mesothelin. J. Hematol. Oncol. *14*, 118. https://doi.org/10.1186/s13045-021-01128-9.
51. Lu, Y., Wang, Y., Liu, W., Ma, H., Yang, B., Shao, K., Long, S., Sun, W., Du, J., Fan, J., et al. (2023). Photothermal "nano-dot" reactivate "immune-hot" for tumor treatment via reprogramming cancer cells metabolism. Biomaterials *296*, 122089. https://doi.org/10.1016/j.biomaterials.2023.122089.
52. Kienzl, M., Maitz, K., Sarsembayeva, A., Valadez-Cosmes, P., Gruden, E., Ristic, D., Herceg, K., Kargl, J., and Schicho, R. (2024). Comparative Study of the Immune Microenvironment in Heterotopic Tumor Models. Cancers (Basel) *16*, 295. https://doi.org/10.3390/cancers16020295.
53. Huang, Q., Wu, X., Wang, Z., Chen, X., Wang, L., Lu, Y., Xiong, D., Liu, Q., Tian, Y., Lin, H., et al. (2022). The primordial differentiation of tumor-specific memory CD8(+) T cells as bona fide responders to PD-1/PD-L1 blockade in draining lymph nodes. Cell *185*, 4049–4066.e25. https://doi.org/10.1016/j.cell.2022.09.020.
54. Rahim, M.K., Okholm, T.L.H., Jones, K.B., McCarthy, E.E., Liu, C.C., Yee, J.L., Tamaki, S.J., Marquez, D.M., Tenvooren, I., Wai, K., et al. (2023). Dynamic CD8(+) T cell responses to cancer immunotherapy in human

regional lymph nodes are disrupted in metastatic lymph nodes. Cell *186*, 1127–1143.e18. https://doi.org/10.1016/j.cell.2023.02.021.

55. Jia, H., Zhao, T., Zou, D., Jia, X., Gao, J., and Song, X. (2016). Therapeutic Injection of a C-Type CpG ODN Induced an Antitumor Immune Response in C57/BL6 Mice of Orthotopically Transplanted Hepatocellular Carcinoma. Oncol. Res. *23*, 321–326. https://doi.org/10.3727/096504016X14570992647041.

56. Li, D., Li, N., Zhang, Y.F., Fu, H., Feng, M., Schneider, D., Su, L., Wu, X., Zhou, J., Mackay, S., et al. (2020). Persistent Polyfunctional Chimeric Antigen Receptor T Cells That Target Glypican 3 Eliminate Orthotopic Hepatocellular Carcinomas in Mice. Gastroenterology *158*, 2250–2265.e20. https://doi.org/10.1053/j.gastro.2020.02.011.

57. Yu, M., Yu, H., Wang, H., Xu, X., Sun, Z., Chen, W., Yu, M., Liu, C., Jiang, M., and Zhang, X. (2024). Tumor-associated macrophages activated in the tumor environment of hepatocellular carcinoma: Characterization and treatment (Review). Int. J. Oncol. *65*, 100. https://doi.org/10.3892/ijo.2024.5688.

58. Luo, F., Li, H., Ma, W., Cao, J., Chen, Q., Lu, F., Qiu, M., Zhou, P., Xia, Z., Zeng, K., et al. (2024). The BCL-2 inhibitor APG-2575 resets tumor-associated macrophages toward the M1 phenotype, promoting a favorable response to anti-PD-1 therapy via NLRP3 activation. Cell. Mol. Immunol. *21*, 60–79. https://doi.org/10.1038/s41423-023-01112-y.

59. Sun, Y., Wu, L., Zhong, Y., Zhou, K., Hou, Y., Wang, Z., Zhang, Z., Xie, J., Wang, C., Chen, D., et al. (2021). Single-cell landscape of the ecosystem in early-relapse hepatocellular carcinoma. Cell *184*, 404–421.e16. https://doi.org/10.1016/j.cell.2020.11.041.

60. Zhang, L., Li, Z., Skrzypczynska, K.M., Fang, Q., Zhang, W., O'Brien, S.A., He, Y., Wang, L., Zhang, Q., Kim, A., et al. (2020). Single-Cell Analyses Inform Mechanisms of Myeloid-Targeted Therapies in Colon Cancer. Cell *181*, 442–459.e29. https://doi.org/10.1016/j.cell.2020.03.048.

61. Reinfeld, B.I., Madden, M.Z., Wolf, M.M., Chytil, A., Bader, J.E., Patterson, A.R., Sugiura, A., Cohen, A.S., Ali, A., Do, B.T., et al. (2021). Cell-programmed nutrient partitioning in the tumour microenvironment. Nature *593*, 282–288. https://doi.org/10.1038/s41586-021-03442-1.

62. Martin, M., Rehani, K., Jope, R.S., and Michalek, S.M. (2005). Toll-like receptor-mediated cytokine production is differentially regulated by glycogen synthase kinase 3. Nat. Immunol. *6*, 777–784. https://doi.org/10.1038/ni1221.

63. Trefts, E., and Shaw, R.J. (2021). AMPK: restoring metabolic homeostasis over space and time. Mol. Cell *81*, 3677–3690. https://doi.org/10.1016/j.molcel.2021.08.015.

64. Zhang, L., Xiang, X., Li, Y., Bu, G., and Chen, X.F. (2025). TREM2 and sTREM2 in Alzheimer's disease: from mechanisms to therapies. Mol. Neurodegener. *20*, 43. https://doi.org/10.1186/s13024-025-00834-z.

65. O'Neill, L.A.J., Kishton, R.J., and Rathmell, J. (2016). A guide to immunometabolism for immunologists. Nat. Rev. Immunol. *16*, 553–565. https://doi.org/10.1038/nri.2016.70.

66. Fan, K., Lin, L., Ai, Q., Wan, J., Dai, J., Liu, G., Tang, L., Yang, Y., Ge, P., Jiang, R., and Zhang, L. (2018). Lipopolysaccharide-Induced Dephosphorylation of AMPK-Activated Protein Kinase Potentiates Inflammatory Injury via Repression of ULK1-Dependent Autophagy. Front. Immunol. *9*, 1464. https://doi.org/10.3389/fimmu.2018.01464.

67. Hoves, S., Ooi, C.H., Wolter, C., Sade, H., Bissinger, S., Schmittnaegel, M., Ast, O., Giusti, A.M., Wartha, K., Runza, V., et al. (2018). Rapid activation of tumor-associated macrophages boosts preexisting tumor immunity. J. Exp. Med. *215*, 859–876. https://doi.org/10.1084/jem.20171440.

68. Liao, S., Wang, Q., Chen, S., Huang, Q., Zhou, L., Liu, H., He, S., and Zhou, Z. (2024). Mito-LND and (E)-Akt inhibitor-IV: novel compounds inducing endoplasmic reticulum stress and ROS accumulation against hepatocellular carcinoma. J. Transl. Med. *22*, 792. https://doi.org/10.1186/s12967-024-05545-5.

69. Chen, H., Yang, Y., Deng, Y., Wei, F., Zhao, Q., Liu, Y., Liu, Z., Yu, B., and Huang, Z. (2022). Delivery of CD47 blocker SIRPalpha-Fc by CAR-T cells enhances antitumor efficacy. J. Immunother. Cancer *10*, e003737. https://doi.org/10.1136/jitc-2021-003737.

70. Yoo, I., Ahn, I., Lee, J., and Lee, N. (2024). Extracellular flux assay (Seahorse assay): Diverse applications in metabolic research across biological disciplines. Mol. Cells *47*, 100095. https://doi.org/10.1016/j.mocell.2024.100095.

71. Li, J., Guo, T., Li, Y., Wang, Q., Du, Y., Li, R., Lin, J., Fu, J., Chen, X., and Luo, S. (2024). Adipose stem cells regulate lipid metabolism by upregulating mitochondrial fatty acid beta-oxidation in macrophages to improve the retention rate of transplanted fat. Stem Cell Res. Ther. *15*, 328. https://doi.org/10.1186/s13287-024-03953-4.

72. Mayberry, C.L., Wilson, J.J., Sison, B., and Chang, C.H. (2024). Protocol to assess bioenergetics and mitochondrial fuel usage in murine autoreactive immunocytes using the Seahorse Extracellular Flux Analyzer. STAR Protoc. *5*, 102971. https://doi.org/10.1016/j.xpro.2024.102971.

CellPress
OPEN ACCESS

STAR★METHODS

KEY RESOURCES TABLE

REAGENT or RESOURCE	SOURCE	IDENTIFIER
Antibodies		
anti-CD3	eBioscience	Cat#16-0031-85
anti-CD28	eBioscience	Cat#16-0281-85
anti-mouse CD40 (FGK45)	BioXCell	Cat#BP0016-2
anti-rat IgG2a isotype control	BioXCell	Cat#BP0089
anti-CD45-Pacifific Blue	Biolegend	Cat#304022
Goat Anti-Rabbit IgG H&L (HRP)	Abcam	Cat#ab205718
Rabbit Anti-mouse CD8 alpha antibody	Abcam	Cat#ab209775
TSA reagent iFluor® 488 tyramide	aatbio	Cat#16985
rabbit Anti-mouse CD4 antibody	Abcam	Cat#ab183685
TSA reagent iFluor® 430 Tyramide	aatbio	Cat#45096
Rabbit anti-mouse IFN-γ antibody	Affinity	Cat#DF6045
TSA reagent CY3 tyramide	aatbio	Cat#11065
Rat anti-mouse PD-1 antibody	Abcam	Cat#ab214421
TSA reagent CY5 tyramide	aatbio	Cat#11066
Rabbit anti-mouse F4/80 antibody	CST	Cat#70076
Rabbit anti-mouse CD3 antibody	Abcam	Cat#ab237721
Rabbit anti-Mouse CXCL9 Antibody	Abcam	Cat#ab202961
Rabbit anti-Mouse SPP1 antibody	Abcam	Cat#ab283656
Rabbit anti-mouse CD3 antibody	Abcam	Cat#ab237721
DAPI	Thermo Fisher	Cat#D1306
anti-CD16/CD32	BioLegend	Cat#101302
PerCP/Cyanine5.5 anti-mouse CD3	Biolegend	Cat#100218
PE anti-mouse CD8a	Biolegend	Cat#100708
Brilliant Violet 421 anti-mouse PD-1	Biolegend	Cat#135217
Pacific Blue anti-mouse IFN-γ	Biolegend	Cat#505818
Pacific Blue anti-mouse CD3	Biolegend	Cat#100213
Alexa Fluor® 488 anti-mouse CD11b	ThermoFisher	Cat#53-0112-82
APC anti-mouse F4/80	Biolegend	Cat#123116
PE anti-mouse CXCL9	Biolegend	Cat#515603
PE anti-mouse SPP1	R&D	Cat#IC808P
human IgG1 antibody	Selleck	Cat#A2051
Sotigalimab	Selleck	Cat#A2635
goat anti-rat immunoglobulin G	BioLegend	Cat#405401
Syk (PT0468R) PT® Rabbit mAb	CST	Cat. #YM83025
Syk (Phospho Tyr525/526) (PT0753R) PT® Rabbit mAb	Immunoway	Cat. #YM8562
Jak1 Rabbit mAb	Immunoway	Cat. #YM8467
JAK1 (Phospho Tyr1022) Rabbit pAb	Immunoway	Cat. #YP0154
STAT1 α Rabbit mAb	CST	Cat. #9172
Phospho-Stat1 (Tyr701) Rabbit mAb	CST	Cat. #9167
SOCS-1 Rabbit pAb	Immunoway	Cat. #YT4362
CD40 Rabbit pAb	Immunoway	Cat.#YT0763
PI3 Kinase p85 Rabbit mAb	CST	Cat. #4292

(Continued on next page)

Continued		
REAGENT or RESOURCE	SOURCE	IDENTIFIER
PI 3 kinase p85/p55 (Phospho Tyr467/199) Rabbit pAb	Immunoway	Cat. #YP0224
Akt1 Rabbit mAb	CST	Cat. #2938
Phospho-Akt (Thr450) Rabbit mAb	CST	Cat.#4060
mTOR Rabbit mAb	CST	Cat.#2972
Phospho-mTOR (Ser2448) Rabbit mAb	CST	Cat. #2971
LDHA Antibody Rabbit	CST	Cat. #2012
AMPKα Rabbit mAb	CST	Cat. # 5831T
Phospho-AMPKα (Thr 172) Rabbit mAb	CST	Cat. #2535T
IDHP Rabbit pAb	CST	Cat. #YT6168
CPT1A (PT0640R) PT® Rabbit mAb	CST	Cat. #YM8449
anti-HA-Tag	CST	Cat. #3724S
anti-β-actin	ABCAM	Cat. #ab6276
Biological samples		
Human tissue samples	The First Affiliated Hospital of Sun Yat-sen University	N/A
Chemicals, peptides, and recombinant proteins		
murine IL-2	R&D	Cat#402-ML-050
RetroNectin	Takara Bio	Cat#T100A/B
cyclophosphamide	Sigma	Cat#C0768
complete DMEM medium	GIBCO	Cat#11054001
Fetal calf serum (FBS)	GIBCO	Cat#16140071
ACK red blood cell lysis buffer	GIBCO	Cat#A1049201
LIVE/DEAD Zombie NIR Fixable Viability Kit	Biolegend	Cat#423105
Intracellular Fixation & Permeabilization Buffer Set	Thermo Fisher	Cat#88-8824-00
Lipofectamine 2000	Thermofisher	Cat#11668019
Opti-MEM medium	GIBCO	Cat#31985070
matrigel matrix	Corning	Cat#354230
rh-IFN-γ	PeproTech	Cat#300-02-100UG
CellTrace Farred	Cat#C34572	ThermoFisher
rm-IFN-γ	PeproTech	Cat#315-05-20UG;
SYK inhibitor R406	Selleck	Cat. #S1533
STAT1 inhibitor Fludarabine	Selleck	Cat. #S1491
AMPK inhibitor Dorsomorphin 2HCl	Selleck	Cat. #S7306
mTOR inhibitor Rapamycin	Selleck	Cat. #S1039
sensor cartridge	Agilent	Cat#103793-100
calibration buffer	Agilent	Cat#103793-100
10 mM glucose	Agilent	Cat #103577-100
1 mM pyruvate	Agilent	Cat#103578-100
2 mM glutamine	Agilent	Cat #103579-100
etomoxir	Selleck	Cat#S8244
Critical commercial assays		
mouse CD8+T cell negative enrichment kit	BD	Cat#558471
Tumor Dissociation Kit	MiltenyiBiotec	Cat#130-096-730
gentle MACS Dissociator	MiltenyiBiotec	Cat#130-093-235
FITC Annexin V Apoptosis Detection Kit II	BD	Cat#556570
human CD3/CD28 Streptamer Kit	IBA	Cat#6-8900-050
ATP Content Assay Kit	Beyotime	S0026
AMP Content Assay Kit	Abcam	ab273275

(*Continued on next page*)

Continued

REAGENT or RESOURCE	SOURCE	IDENTIFIER
Seahorse 96-well culture plates	Agilent	Cat#103793-100
XF DMEM assay medium	Agilent	Cat#103575-100
Seahorse XF Glycolytic Rate Assay Kit	Agilent	Cat#103015-100
Seahorse XF Glycolytic Rate Assay Kit	Agilent	Cat#103344-100
Mouse IL-6 ELISA Kit	MultiSciences	Cat#70-EK206/3-96
Mouse IFN-γ (Interferon Gamma) ELISA Kit	MultiSciences	Cat#E-HSEL-M0007
**Deposited data**		
single-cell sequencing of HCC patients	Gene Expression Omnibus	no. GSE253352
Original western blot images	This paper	Supplemental figures
Original data of the single-cell sequencing of mice	This paper	https://www.ncbi.nlm.nih.gov/bioproject/PRJNA1303879/
**Experimental models: Cell lines**		
Hepa1-6 cell line	ATCC	Cat#CRL-1830
233T cell line	ATCC	Cat#CRL-1573
Human vascular endothelial (VE) cells	Procell	Cat#CRL-0310
4T1 cell line		Cat#CRL-2539
**Experimental models: Organisms/strains**		
C57BL/6-Trem2em1Smoc	Shanghai Model Biological Center	Cat#NM-KO-190402
**Oligonucleotides**		
Primers	See Table S1 for primers	N/A
**Recombinant DNA**		
anti-human GPC3 scFv- mouse CD8α	This paper	N/A
antihuman CD19 scFv-mouse CD8α	This paper	N/A
**Software and algorithms**		
Prism 9.0	GraphPad	https://www.graphpad.com/scientific-software/prism/
FlowJo v10	TreeStar	https://www.flowjo.com/solutions/flowjo/downloads
Imaris (version 7.4)	BITPLANE (Oxford Instruments)	https://imaris.oxinst.com/; RRID: SCR_007370
ImageJ	National Institutes of Health	https://imagej.en.softonic.com

EXPERIMENTAL MODEL AND STUDY PARTICIPANT DETAILS

Mice and *in vivo* studies

Male *Trem2*-KO mice (C57BL/6-*Trem2*em1Smoc) were purchased from Model Organisms, Shanghai, China, which knockout exon2 zone of *Trem2*. The wild-type (WT) control male C57BL/6 mice were purchased from the same Model Organisms, and were used as WT controls. For HCC subcutaneous tumor model, C57BL/6J mice were subcutaneously inoculated with GPC3-Hepa1-6 cells of 5×10^5 cells. Fifteen days post-inoculation, mice received an intravenous tail vein injection of Control (Ctrl)-CAR-T or GPC3-CAR-T cells of 1×10^6 cells. Tumor volumes were measured every 3–4 days. Survival rates were monitored at the endpoint of death or 2000 mm^2 tumor volume. For HCC orthotopic tumor model, 5×10^5 Hepa1-6-GPC3 cells or 4T1 cells were injected in 6-week-old mice orthotopically into the liver. 13days after tumor inoculation the cyclophosphamide at 80 mg/kg was administered intraperitoneally (i.p.), and 1×10^6 GPC3-CAR-T cells or Trop2-CAR-T cells were injected intravenously at next day. For antibody treatment, 10 mg/kg *InVivo* Plus anti-mouse CD40 or rat IgG2a isotype control was administered by i.p. injection every 3 days after the injection of CAR-T cells. All experimental protocols were approved by the Animal Care Committee of Sun-Yat sen University (SYSU-IACUC-2022-000394).

Cell lines

Hepa1-6, an HCC cell derived from C57BL/6 was transduced with recombinant retroviruses carrying human GPC3 and GFP moiety to establish Hepa1-6-GPC3$^+$GFP$^+$ cells, followed by FACS sorting. 4T1, a breast cancer cell derived from BALB/C was transduced with recombinant retroviruses carrying human Trop2 and GFP moiety to establish 4T1-Trop2$^+$GFP$^+$ cells, followed by FACS sorting.

These cell lines were maintained in Dulbecco's modified Eagle's medium (DMEM) (Gibco, Grand Island, NY, USA). Media were supplemented with 10% heat-inactivated FBS, 10 mM HEPES, 2 mM glutamine and 1% penicillin/streptomycin. All cells were cultured at 37°C in an atmosphere of 5% carbon dioxide.

Human tissue samples

Tumor samples were obtained from patients with hepatocellular carcinoma (HCC) after obtaining written informed consent. All procedures were approved by the Institutional Ethics Committee of The First Affiliated Hospital of Sun Yat-sen University (protocol 2024[400]) under the supervision of Professor Wenzhe Fan. A total of 12 patients with HCC at Interventional oncology of The First Affiliated Hospital of Sun Yat-sen University from 2023 to 2024 were enrolled in the research. Samples were taken by needle biopsy before treatment, and then received drug-eluting beads transcatheter hepatic arterial chemoembolization (DEB-TACE) plus anti-PD-1 therapy. Based on treatment response, six patients were classified as responders and six as non-responders (age range 36–71 years, median 57.5; all males). Additionally, surgical specimens were obtained from 5 HCC patients (age range 49–54 years, median 52; all males) enrolled from 2024 to 2025. Fresh tissues from these patients were used for macrophage isolation and preparation of digested tumor solution for *in vitro* studies.

METHOD DETAILS

CART generation

The human-GPC3-specific murine CAR was generated by linking antihuman GPC3 single-chain variable fragment (scFv) to the mouse CD8α hinge domain, mouse CD28 transmembrane and the cytoplasmic domain, and the intracellular signaling domain of mouse CD3ζ. The human Trop2-specific murine CAR (T2-m28z) was generated by linking anti-human Trop2 scFv to the mouse CD8α hinge domain, mouse CD28 transmembrane and the cytoplasmic domain, and the intracellular signaling domain of mouse CD3ζ. For negative control, the human-CD19-specific murine CAR was generated by linking antihuman CD19 scFv to the mouse CD8α hinge domain, mouse CD28 transmembrane and the cytoplasmic domain, and the intracellular signaling domain of mouse CD3ζ. CAR genes were then cloned into the retroviral vector MIGR1 which was used as previously described.[69] 293T cells were transfected with the CAR-expressing retrovirus plasmid and pCL-Eco packaging plasmid by using calcium phosphate method. The culture supernatants were harvested and frozen for gene transduction after 48 h. Mouse T cells isolated from WT C57BL/6 mouse splenocytes were purified with a mouse $CD8^+$T cell negative enrichment kit. T cells were activated with 0.25 μg/mL hamster anti-CD3, 1 μg/mL hamster anti-CD28 and 10 ng/mL murine IL-2 for 24 h. The activated T cells were then infected with retroviral supernatants in the presence of RetroNectin and transduction efficacy was determined in GFP^+ with flow cytometry analysis.

Single cell suspension preparation for tumor of mice

Fresh HCC tumor samples were surgically removed from mouse liver and transported by complete DMEM medium and 10% FBS in ice container. Selected samples were cut into small pieces of diameter 1mm, and removed necrotic, hemorrhagic and fibrous connective tissues. Fragments of tissue samples were digested with 5mL enzyme in Tumor Dissociation Kit. Then tissue were digested into single cell suspension by gentle MACS Dissociator followed by ordering instructions. Digested cells went through a 70 mm nylon mesh filter and then a 40 mm nylon mesh filter. Filtered cell suspensions were centrifuged and resuspended in 5 mL ACK red blood cell lysis buffer for 5 min, then immediately diluted with 10 mL of PBS buffer containing 0.5% FBS. Cells were centrifuged and subsequently resuspended in viability dye solution (1:500 in PBS) with FITC Annexin V Apoptosis Detection Kit II, followed by 15 min incubation in the dark. Following viability dye staining, cells were allocated for testing viability and sorting for single-cell RNA-seq by flow cytometry.

Sorting for live $CD45^+$ cells

Cells stained with viability dye (1×10^7) were resuspended in 100 mL of FACS wash buffer (PBS+2%FBS, 1mM EDTA) with the addition of the following antibodies: CD45-Pacifific Blue. Cells were incubated on ice for 20 min, washed with 1 mL PBS buffer containing 0.5% FBS, and then resuspended in 500 mL PBS buffer containing 0.5% FBS for sorting on a FACS Aria II. For most samples, 1×10^5 live $CD45^+$ cells were sorted into lymphocyte media (RMPI+10%FBS) and immediately transferred to ice.

Single cell library construction

The previous sorted $CD45^+$ single cells were loaded to the single-cell chip for GEM (Gel bead-in-emulsions) generation using the 10x Chromium controller to construct single cell library (https://www.10xgenomics.com/solutions/single-cell/). The single cell 5′ cDNA and TCR V(D)J library were constructed using the Chromium Single cell 5′ Regent Kit v3 and Single cell V(D)J Reagent kits (10x Genomics). In the kits, gel beads coated with unique primers consisting of 10x cell barcodes, unique molecular identifiers (UMI) and poly-dT sequences were used to separately index the transcriptomes of each cell. After reverse transcription and amplification, the constructed libraries were sequenced on the Illunima NovaSeq 6000 platform to generate the 2x 150-bp paired end reads of which R1 holds the UMI and cell barcodes and R2 holds transcriptomes.

Single cell RNAseq (scRNAseq) preprocess

The sequenced raw fastq reads were processed by Cell Ranger toolkit (10x Genomics, version 7.0.0) with "count" sub program to generate the count matrix and immune repertoire of TCR. Reference genome (version refdata-gex-mm10-2020-A-GFP-CART, see below) was used for read alignment. The gene-barcode matrix of UMI counts were then analyzed with Seurat (version 4.0.4) for quality control, data normalization, batch effect removal, dimensional reduction, clustering and visualization. Quality control on scRNAseq data was performed by filtering cells expressing less than 500 genes or more than 6000 genes. We also filtered out cells with more than 10% transcripts from mitochondrial genes. Then R package DoubletFinder (version 2.0.3) was used to identify the potential doublet cells which would be filtered out from the count matrix. In addition, cells expressing multiple canonical cell type-specific markers were also filtered out. In the end, we retained a total of 83191 cells for further downstream analysis.

Detection of CAR-T insertion and *Trem2* deletion in cells

To identify the CAR-T insertion status in cells, we integrated the sequences of GFP-CAR transcripts into the original reference genome as an extra chromosome-like sequence, then rebuilt the refdata-gex-mm10-GFP-CART reference genome with Cell Ranger "mkref" sub program. CAR-T cells were identified by the detection of GFP or CAR transcripts. To identify the *Trem2* deletion status, we first extracted reads mapped to *Trem2* in each cell from the bam file generated by Cell Ranger and assigned reads to the exact exons in *Trem2*. Based on the validation result of our knock-out experiment, expression of exon-2 in *Trem2* was used to identify the deletion status and the expression level of *Trem2*.

Identification of cell clusters

Based on the quality-controlled count matrix, R package Seurat was applied for downstream analysis. Function "SCTransform" from Seurat was used for gene expression normalization and highly variable gene identification with default parameters. To avoid the cluster enriched by highly expression of mitochondrial genes or other similar situations, mitochondrial genes were removed, and cell cycle effect was regressed out. Principal component analysis was performed by "RunPCA" function. To remove the potential batch effects between batches, function "RunHarmony" from the R package harmony (version 0.1.0) was applied on the PCA matrix. After batch correction, function "ElbowPlot" in Seurat was used to identify the significant corrected components for cell clustering. Dimension reduction based on the selected components were performed by uniform manifold approximation and project (UMAP) and t-distributed stochastic neighbor embedding (tSNE) via the function "RunUMAP" and "RunTSNE". The selected components were also used to get the final clusters with the specific resolution parameters by function "FindNeighbors" and "FindClusters". Finally, we utilized the function "FindAllMarkers" function to detect the cluster-specific expressed genes which were identified as > 0.25-fold difference (log-scale) on average between two groups of cells and detectable expression in more than 25% of cells in either of the two cell populations. For the clustering of all cells and sub clustering of monocytes and macrophages, the top 30 PCs were selected with a resolution parameter 0.6. For the sub clustering of $CD4^+$ and $CD8^+$ T cells, the top 30 PCs and resolution 0.8 were used. Based on the cell clustering, we annotated the cell clusters manually with canonical cell type-specific markers, including B cells (*Ms4a1, Cd79a, Cd79b, Jchain, Sdc1*, the last 2 additionally for plasma cell), T cells (*Cd3d, Cd3e, Cd3g, Cd4* for CD4 T cells, *Foxp3* and *Ctla4* for Treg, *Cd8a* and *Cd8b1* for CD8 T cells), NK cells (*Ncr1, Ncam1, Prf1, Klrd1*), cDC (*Clec9a, Cd209a, Xcr1, Ccr7, Fscn1, Ccl22*), pDC(*Lilra4, Irf8, Siglech*), Monocytes or Macrophages (*Ms4a4c, Cd14, Cd68, Csf1r, Adgre1, C1qa, C1qb, C1qc*), Neutrophils (*Csf3r, G0s2, s100A8, S100A9, Clec4a, Ly6g*), Basophils (*Prss34, Mcpt8, Cpa3, Cd200r3*), Platelet (*Pf4, Ppbp, Gp9*), fibroblast (*Dcn, Col3a1*), endothelial cells (*Eng, Kdr*), epithelial cells (*Krt8, Epcam*). The same workflow was performed for the sub-clustering of each interested cell type and sub-clusters were annotated based on the cluster markers and their functionality.

$CD8^+$ T cell subsets were classified as follows: γδ T cells were identified based on TCR γ/δ chain expression. CD8_Tn (naive) was characterized by CCR7, LEF1, and TCF7. CD8_Tcm (central memory) was defined by IL7R, TCF7, and SELL. CD8_Tem (effector memory) exhibited an effector-memory profile by IL7R, IFNG, and GZMM. CD8_Trm (tissue-resident memory) was identified by CXCR3, CXCR6, and TNF. CD8_Te (effector cytotoxic) displayed high expression of cytotoxicity-related genes of GZMB, PRF1, but lacked exhaustion markers. CD8_prolif (proliferating) was two clusters showing proliferation signatures of MKI67, and TOP2A. CD8_Tex (exhausted) was further stratified into three progressive states based on exhaustion marker levels of PDCD1, LAG3, and HAVCR2. CD8_Tex-prog (progenitor exhausted) was low exhaustion signature. CD8_Tex-int (intermediate exhausted) was moderate exhaustion. CD8_Tex-term (terminally exhausted) was high exhaustion. Th17 cells exhibited high expression of IL17A, IL23R, and RORC. CD4_Tcm (central memory T cells) displayed a canonical memory phenotype, marked by CCR7, LEF1, and SELL. CD4_prolif, a proliferative subset, was characterized by elevated MKI67 and STMN1. The Tfh (follicular helper T cell) cluster expressed key markers such as CXCR5, BCL6, and BTLA. Th1_effector cells showed robust expression of cytotoxicity-associated genes but minimal exhaustion-related signatures. In contrast, Th1_exhausted cells exhibited high levels of exhaustion markers alongside moderate cytotoxicity gene expression. Two distinct Treg populations were identified: Treg_blood, expressing FOXP3, IL2RA, and IKZF2; and Treg_tumor, which upregulated FOXP3, TNFRSF9, and TNFRSF18.

For macrophages, C0_Monocyte_*Ly6c2* displayed a monocyte-like phenotype, marked by high expression of *Ly6c2, Lyz2* and *S100A4*. C1_Macro_*Cd74* represented inflammatory TAMs, characterized by *IL1b, Cxcl2* and *Ccl2*. C3_Macro_*Mki67* was identified as a proliferating subset. C4_Macro_*Spp1* exhibited immunosuppressive and pro-angiogenic features of *Spp1, Arg1*, and *Vegfa*. C6_Macro_*Apoe* resembled lipid-associated TAMs, with elevated *Apoe, Trem2* and *C1qa/b/c*. C7_Macro_*Cxcl9* demonstrated

pro-inflammatory and antigen-presenting capabilities expressed *Cxcl9, Cxcl10* and MHC class II genes. C8_Macro_*Ace*matched a previously described subset expressed *Ace, Adgre4* and *Nr4a1*. C12_Macro_*Rsad2* was enriched for interferon-inducible regulatory genes. The remaining clusters were annotated based on their top marker genes, with no direct correlates in existing literature.

Differential expressed gene analysis

Function "FindMarkers" in the Seurat was used to identify the differential expressed genes between different groups (KO vs. WT, GPC3-CART vs. NC-CART) in macrophages, $CD8^+$ T cells and $CD4^+$ T cells. Significant differential genes were identified by absolute log2-scale fold change >0.25 and adjusted *p* value <0.05 by Wilcoxon Rank-Sum Test.

Single cell TCRseq (scTCRseq) data analysis

The sequenced raw TCR fastq reads were also processed by Cell Ranger toolkit with "vdj" sub program and the R package scRepertoire (version 1.0.0). Briefly, fastq reads were aligned to mouse GRCm38 V(D)J reference genome (version refdata-cellranger-vdj-GRCm38-alts-ensembl-7.0.0) to generate the assembled TCR contigs, corresponding annotation to CDR3 region (V, D, J, C genes) and TCR clonotypes. Quality control on scTCRseq data was performed by filtering clonotypes with no "raw_consensus_id" and non-productive TCR. TCR that had no cells in filtered cells from scRNAseq data were also removed. After quality control, each TCR was assigned to corresponding cells in scRNAseq by their cell barcode. For cells with only one TRA or TRB chain, cells with identical TRA and TRB chains were defined as a T cell clone. For cells with paired TRA and TRB chains, cells shared at least one pair of identical TRA and TRB chains were defined as one clone, while identical paired TRA and TRB which were shared by three or more cells were defined as an expanded clone. A shared clone was an expanded clone from which cells were distributed in different clusters in each individual. The number of shared clone types were calculated by the sum of numbers of the shared clones. We grouped these TCR clonotypes based on their expansion level into single ($x = 1$), small ($1 < x <= 5$), medium ($5 < x <= 20$), large($20 < x < 100$), hyper ($100 < x$, which was not observed), where x was the number of cells sharing the same clonotype. To investigate the correlation between clonal expansion and anti-tumor function in $CD8^+$ T cells, the weighted Pearson correlation was used to adjust the size effects among different TCR clonotype groups.

Cell development trajectory for $CD8^+$ T cells

The cell lineage trajectory between different functional clusters of $CD8^+$ T cells was inferred by Monocle2 (version 2.18.0). During the trajectory construction, clusters like $\gamma\delta$ T cells and proliferative cells were removed according to their TCR identity or fixed phenotypes related to other $CD8^+$ T cells. Using function "as.CellDataSet" in Seurat, we converted the "Seurat object" to "Monocle CellDataSet". The converted data object was then analyzed following the Monocle2 workflow. The differential genes were identified by function "differentialGeneTest" in monocle and used to order the cells in the pseudotime analysis.

Definition of cell scores and signatures

To illustrate the cell function or status in each cell cluster, a set of genes was collected for visualization. The function "AddModuleScore" in Seurat was used for scoring relative change of such gene sets between clusters. For the functional annotation of T cells, four states were identified by the expression of related genes: a) Exhaustion (*Havcr2, Ctla4, Tox, Tigit, Lag3, Pdcd1*), b) Proliferation (*Mki67, Top2a, Ube2c, Stmn1, Pclaf, Birc5*), c) Cytotoxicity (*Gmza, Gzmb, Gzmk, Gzmm, Ifng, Nkg7, Tnf, Fasl*), d) Memory or Naive (*Ccr7, Il7r, Sell, Tcf7, Lef1*). To annotate the anti-viral or anti-tumor functionality in T cells, gene sets highly expressed by T cells with the experimental validated anti-viral or anti-tumor function were used: a) anti-viral (*Anxa1, Il7r, Ccr7, Gpr183, Tcf7, Glul, Perp, Tc2n, Epha4, Itga5, Klrg1, Cd300a, Klf3, Sell, Trmo, Dkk3, Gpr132, Aoah, Aust2, Samd3*), b) anti-tumor (*Mcm5, Itgae, Linc01871, Dusp16, Layn, Krt86, Igflr1, Slc2a8, Clic3, Kir2dL4, Cxcr6, Entpd1, Tox, Lmna, Batf, Cd27, Syngr2, Ctsw, Lag3, Lsp1, Ahi1, Tnfrsf18, Gem, Mtss1, Gzmb, Ptms, Acp5, Havcr2, Phlda1, Ctla4, Pdcd1, Rgs2, Hmox1, Id3, Cxcl13, Tnfrsf9, Vcam1, Hspb1, Hla-dra, Rgs1*). Genes were converted to homologous genes in mouse and scored by "AddModuleScore".

Pathway analysis

For the pathway annotation or metabolism phenotypes including glycolysis, TCA cycle and fatty acid metabolism of macrophages, gene sets related to each pathway and phenotype from GO/KEGG/MSigDB/PathCards were used as inputs to calculate average expression level in each sub-cluster by function "AverageExpression" and were then analyzed by Gene Set Variance Analysis (GSVA, version 1.38.2) for comparison.

Survival analysis and validation on TCGA

The Cancer Genome Atlas (TCGA) liver hepatocellular carcinoma (LIHC) data were used to evaluate the prognostic performance of individual genes or gene sets derived from specific cell clusters by the R package survival (version 3.2.7). The gene expression data and the clinical data were obtained from the GDC website (https://portal.gdc.cancer.gov/), UCSC Xena (http://xena.ucsc.edu/) and GEPIA2 (http://gepia2.cancer-pku.cn/#index). The gene expression of each cell signature was defined using the TPM or FPKM and visualized in the log2-scale. For cell signature in myeloid cells, gene expressions were normalized by the expression of canonical genes *C1qb, Lyz, Aif1, Cd68, Cd163* and *Cst3*. For cell signature in T cells, gene expressions were normalized by the expression of *Cd3d, Cd3e, Cd3g, Cd4, Cd8a, Cd8b* and *Foxp3* for each T cell sub lineages. For cell signatures in immune cells, PTPRC were

used for normalization. For the cytotoxic score, the mean expression of *Gzma, Gzmb, Gzmk, Gzmm, Ifng, Nkg7, Tnf* and *Fasl* were used. To validate the clinical effect of CXCL9 and SPP1 on prognosis, samples in LIHC were grouped into high or low groups based on the median values of expression ratio of *CXCL9* to *SPP1*. To validate the metabolism effects mediated by *SPP1* or *CXCL9*, the correlation between *SPP1* or *CXCL9* and metabolism related genes like *SCL2A*, *VEGFA* was also identified in the LIHC. Kaplan–Meier survival curves were plotted to show differences in overall survival time or disease-free survival time, and hazard ratio and log rank *p* values reported by the Cox regression models implemented in the R package survival were used to determine the statistical significance.

Reverse transcription Quantitative PCR (RT-QPCR)

The renal mRNA expression levels of IL-1βand TNF-αwere assessed using qPCR. Total RNA was extracted from tissues using Trizol reagent, following the supplier's instructions. Gene expression was normalized to β-actin, and relative transcript levels were calculated using the $2^{-\Delta\Delta C_t}$ method. Primer sequences used are listed in Table S1.

Multiple immunofluorescence staining

For immunofluorescence, sections of formalin-FIxed, paraffin-embedded tumors from mice were dewaxed in xylene, rehydrated through an alcohol gradient, and subjected to antigen retrieval in EDTA buffer (pH 8.0). Subsequently, sections were incubated with goat serum for 30 min at 37°C and incubated with primary antibodies overnight at 4°C, then with secondary antibodies 45min at 37°C. For details, all the primary antibodies were rabbit-derived mabs, and all secondary antibodies were Goat Anti-Rabbit IgG H&L (HRP). After staining with the primary and secondary antibodies, the corresponding TSA reagents were used for color development. DAPI was used for the staining of the nucleus. Fluorescent signals were detected using the laser scanning confocal microscope (ZEISS LSM 800). To quantify immunofluorescence results, the images were further analyzed with Imaris software (Version 7.4, BITPLANE). The "Spot" function was used to locate and enumerate cells based on size and intensity threshold. Alternatively, the absolute numbers of cells spotted per mm^2 in nine high-power fields of interested areas were also statistically analyzed (three separate fields from each mouse and three mice from each group).

Flow cytometry

Tumor cell suspensions were centrifuged and resuspended. Cells were resuspended in FACS buffer with 5μg/ml anti-CD16/CD32 for blocking Fc receptors and then first stained with LIVE/DEAD Zombie NIR Fixable Viability Kit for 30 min at 4°C. Following antibodies for cell surface staining were added for 30 min. Following incubation, cells were washed for intracellular staining. Cells were then fixed and permeabilized by Intracellular Fixation & Permeabilization Buffer Set for 30 min. Cells were washed before proceeding to intracellular staining with the antibody. Flow cytometric analysis was performed on a FACS Canton II or FACS Fortessa (BD, USA).

Bone marrow-derived macrophages (BMDMs) isolation and culture

BMDMs were isolated from tibiae of 4-6-week-old *Trem2* knockout (KO) and WT C57BL/6J mice as previously described,[20] with modifications. Briefly, bone marrow cells were flushed from dissected tibiae using cold PBS. After red blood cell lysis ACK buffer, cells were plated at 1×10^6 cells/mL in petri dishes and cultured in DMEM supplemented with 10% heat-inactivated FBS, 1% penicillin/streptomycin, and 30% L929 cell-conditioned supernatant (as a source of M-CSF) for 7 days, with media refreshment on day 3. Differentiated BMDM purity was consistently >95% as confirmed by flow cytometry analysis of CD11b and F4/80 expression.

Plasmid construction and cell transfection

Plasmids containing murine *Socs1* with an HA tag was constructed. Full length genes were amplified by reverse transcription-PCR and cloned into PSG5 vector. The constructed plasmid was confirmed by sequencing and their expressions in 293T cells were detected by western blot with anti-Tag antibodies. Immortalized BMDMs (iBMDM) were transfected with negative control (empty PSG5 vector control) or constructed plasmids using Lipofectamine 3000 according to the manufacturer's instructions. 6 h post transfection, Opti-MEM medium was discarded and refreshed with complete DMEM medium with 10% (v/v) FBS, L-Glutamine (2 mM), and penicillin-streptomycin (100 U/ml). 48 h later, cells were processed for the following experiments.

$CD8^+$ T cell transendothelial migration assay

Human vascular endothelial (VE) cells were seeded (10,000/well) on a transwell insert that was precoated with 10% matrigel matrix). The VE cells were cultured for 24 h to form a compact monolayer, which was verified by visual inspection using an inverted microscope. Then, TAMs (5×10^5) sorted by MACS as previous described,and human GPC3 positive digested tumor suspension (5×10^5) or human GPC3-CART cells (2.5×10^5) were added to the lower chamber of each transwell. The lower chambers were filled with RPMI plus 10% fetal calf serum, along with rh-IFN-γ (40 ng/mlA) or Sotigalimab (50ng/ml), which was a humanized monoclonal antibody. And a human IgG1 antibody was used as isotype control (50ng/ml) in TAMs only and TAMs+IFN-γ group. Digested tumor suspension (DTS) was derived from $GPC3^+$ HCC fresh samples surgically removed. The $CD8^+$ T cells were sorted from peripheral blood mononuclear cells and activated by the human CD3/CD28 Streptamer Kit for 24h. Then, $CD8^+$ T cells were dyed by CellTrace Farred, and then added to the upper layer of the transwell 24 h later. After 12h incubation, the medium in the lower chamber was collected, and the suspended cells that migrated were manually counted using a flow cytometry.

In vitro tumor-educated macrophage model

BMDMs or the immortalized BMDM (iBMDM) cell line were plated into 6-well transwell plates at a density of 5×10^5 cells per well and incubated for 4 h to allow adherence. Subsequently, digested Hepa1-6 cell suspension was added to the upper chamber containing a 0.4 μm porous membrane at a density of 5×10^5 cells per well. In the lower compartment, IFN-γ (40 ng/mL) was added to induce macrophage polarization as required by the experimental design. For inhibitor studies, BMDMs were pre-treated with inhibitors for 2 h prior to co-culture: SYK inhibitor R406 (1 μM), STAT1 inhibitor Fludarabine (100 μM), AMPK inhibitor Dorsomorphin 2HCl (20 μM), mTOR inhibitor Rapamycin (1 μM). Equivalent solvent controls (e.g., 0.1% DMSO) were included. For CD40 agonism, anti-mouse CD40 (FGK45; 20 ng/mL) was crosslinked with goat anti-rat immunoglobulin G for 30 min at room temperature before being added to the DMEM culture medium. Complete control groups: Rat IgG2a isotype control (20 ng/mL); IFN-γ (40 ng/mL) + IgG2a; FGK45 alone; IFN-γ + FGK45. Cells were co-cultured for 24 h (standard). Finally, BMDMs or iBMDMs were co-cultured with Hepa1-6 cells for 24 h, after which macrophages were collected at the indicated time points for RNA extraction and Western blot analysis.

Intracellular ATP and AMP measurement

The intracellular ATP or AMP levels of BMDM or iBMDM cultured in 6-well plates for 24 h were measured using the ATP Content Assay Kit or the AMP Content Assay Kit. Cells were collected and mixed with extraction buffer following the instructions of the ATP and AMP assay kit. The solution was maintained at 0°C for 10 min, after which it was centrifuged to isolate the supernatant fraction. A multifunctional microplate reader was used to measure the ATP or AMP content.

Western blot

Equivalent amount (20 μg) of protein extracts were separated by SDS-PAGE electrophoresis and then transferred to polyvinylidene difluoride (PVDF) membrane. Membranes were blocked in PBS-Tween20 (pH 7.4, 0.5% Tween 20) containing 5% BSA for 1 h at room temperature (RT) and then incubated overnight at 4°C with primary antibodies. Then the membranes were incubated with appropriate horseradish peroxidase (HRP)-conjugated secondary antibodies at RT for 1 h, followed by the visualization with GE Image Quant LAS 500 using an ECL kit (Fdbio Science).

Seahorse XF Cell Mito stress assay and seahorse XF Cell glycolysis rate assay

Oxygen consumption rate (OCR) and extracellular acidification rate (ECAR) were measured using the Agilent Seahorse XFe96 Analyzer, following the manufacturer's instructions. In brief, the sensor cartridge was hydrated overnight in a 37°C incubator with calibration buffer. TAMs ($3 \times 10\wedge^5$ cells/well) were then seeded in Seahorse 96-well culture plates for 12 h. The culture medium was washed twice with detection solution and replaced with 180 μL of fresh detection solution. The detection solution consisted of XF DMEM assay medium (pH 7.4), supplemented with 10 mM glucose, 1 mM pyruvate, and 2 mM glutamine. OCR was assessed through the acute injection of the Seahorse XF Glycolytic Rate Assay Kit and etomoxir, with the following final concentrations of inhibitors: oligomycin (4 μM), FCCP(1.6 μM), rotenone (0.5 μM), antimycin A (0.5 μM), and etomoxir (18 μM). ECAR levels were also measured using the Seahorse XF Glycolytic Rate Assay Kit and etomoxir, with cells treated with rotenone (0.5 μM), antimycin A (0.5 μM), etomoxir (18 μM), and 2-DG (50 mM). Each condition was performed in 3 replicates, and the OCR and ECAR readings from each well were normalized to the protein content.[70–72]

QUANTIFICATION AND STATISTICAL ANALYSIS

Statistical analyses were performed using Graphpad prism 9.5. Spearman's correlation coefficient was used for correlation matrices. Kaplan–Meier survival curves were compared by the log rank test. Data samples were compared using the Wilcoxon test. For immunofluorescence quantification, cell counts from three random fields per sample were averaged, with n representing biologically independent samples. Statistical significance was determined using Student's *t* test (two groups) or ANOVA with Tukey's multiple comparison test (three or more groups). Data were presented as mean ± SEM. $p > 0.05$ (NS), *$p < 0.05$, **$p < 0.01$, ***$p < 0.001$, and ****$p < 0.0001$ were considered statistically significant.

Cell Reports Medicine, Volume *7*

Supplemental information

CAR-T triggers TAM reeducation and adaptive anti-tumor response via *TREM2* deficiency or CD40 agonist

Ting Liu, Huixin Gao, Zhihui Xi, TianTian Yu, Yimei Gu, Hanbing Mai, Hui Yuan, Yafang Liu, Haikuan Liu, Qiaoxuan Zhang, Xianzhang Huang, Wenzhe Fan, and Jizhou Tan

Supplemental information

Supplemental Figures and figure legends

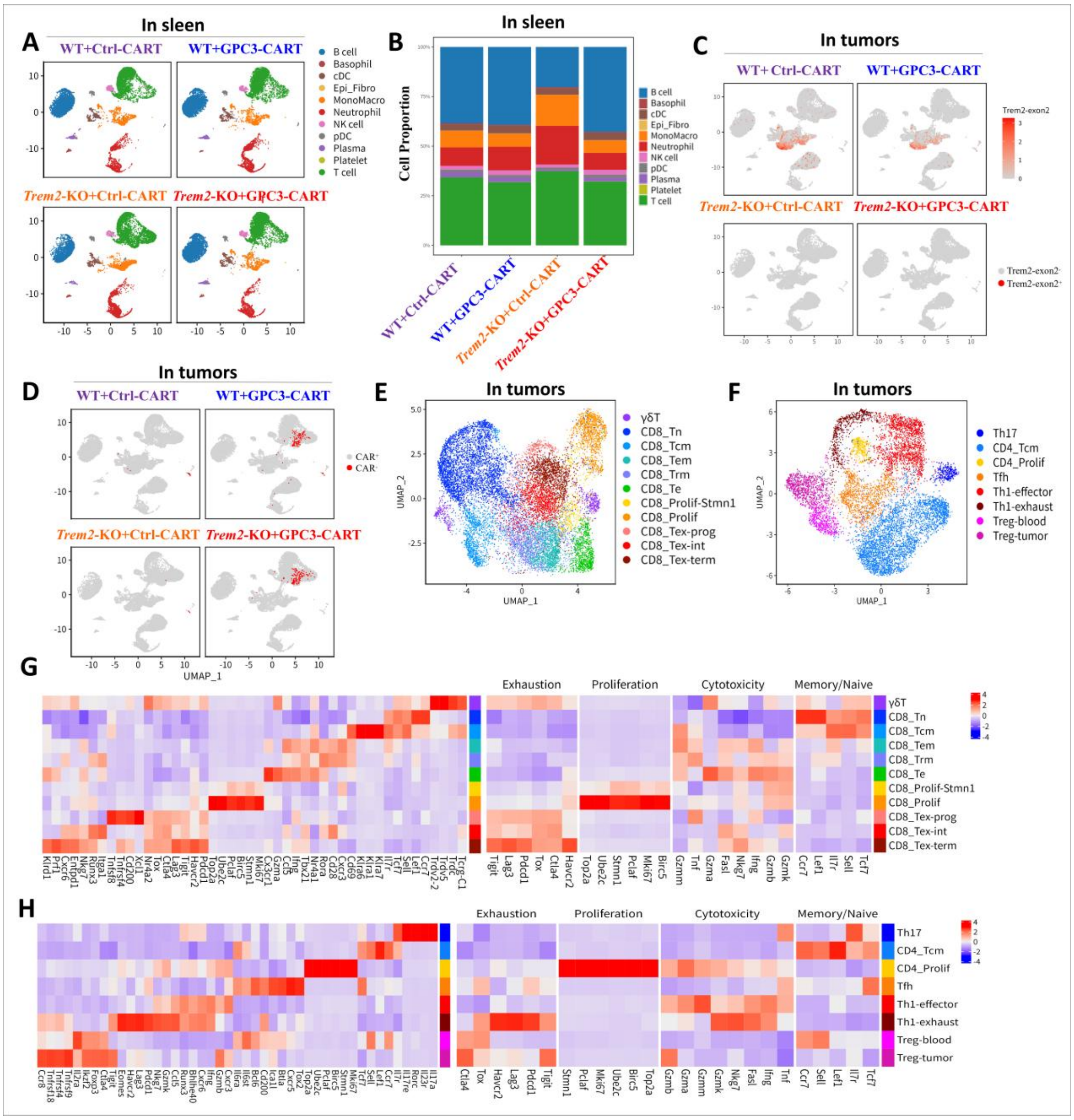

Figure S1. Cell clusters and corresponding markers in CD8^{+} and CD4^{+} T cells, related to Figure 1 and Figure 2.

(A) UMAP plots of individual group displayed distinct intrasplenic cell populations in spleen. N=2 for all treatment groups on scRNA sequence.

(B) Proportion of the distinct intrasplenic cell populations.

(C) Normalised expression of the indicated *Trem2*-exon2 in each tumor group.

(D) Normalised expression of the indicated CAR-T in each tumor group.

(E-F) Cell distribution in CD8$^+$ T (C) and CD4$^+$ T cells colored by distinct functional clusters.

(G) Heatmap of normalized expression level of top 4-6 cluster markers in each CD8$^+$ T cell clusters (left panel). Heatmaps express as Z score of gene collections on behalf of exhaustion, proliferation, cytotoxicity, and memory/naive phenotypes. CD8$^+$T subtypes are divided based on their function (right panel).

(H) Heatmap of normalized expression level of top 4-6 cluster markers in each CD4$^+$ T cell clusters (left panel). Heatmaps express as Z score of gene collections on behalf of exhaustion, proliferation, cytotoxicity, and memory/naive phenotypes from CD4$^+$T subtypes (right panel).

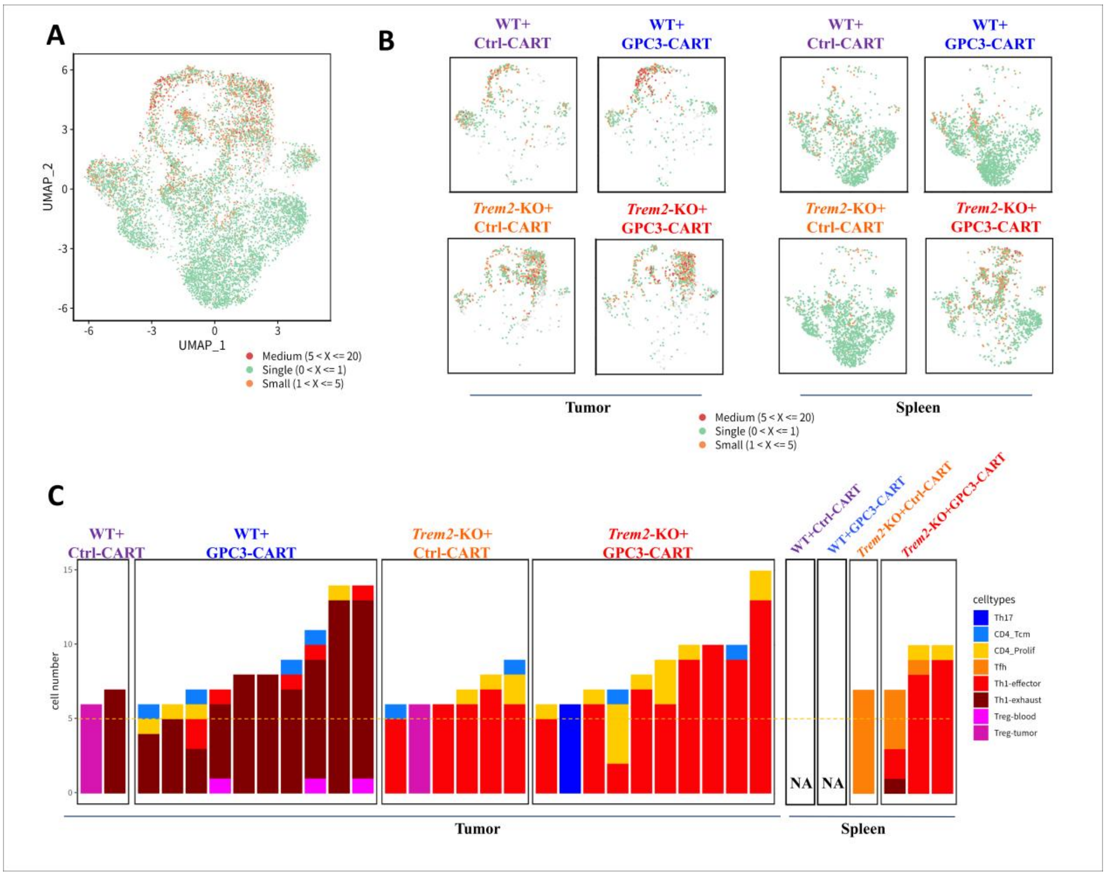

Figure S2. related to Figure 3.

(A) TCR expansion level of CD4$^+$ T cells in UMAP colored by different clonotype groups.

(B) Same to A. The plot was faceted by different groups.

(C) The bar chart revealed the number of TCR clones in each group. Each bar on the X-axis represents a TCR clone. The Y-axis represented the cell number of each TCR. The red dotted line represented the dividing line of large expansion TCR clone, and the orange dotted line represented the dividing line of

medium expansion TCR clone. Cell types were also colored.

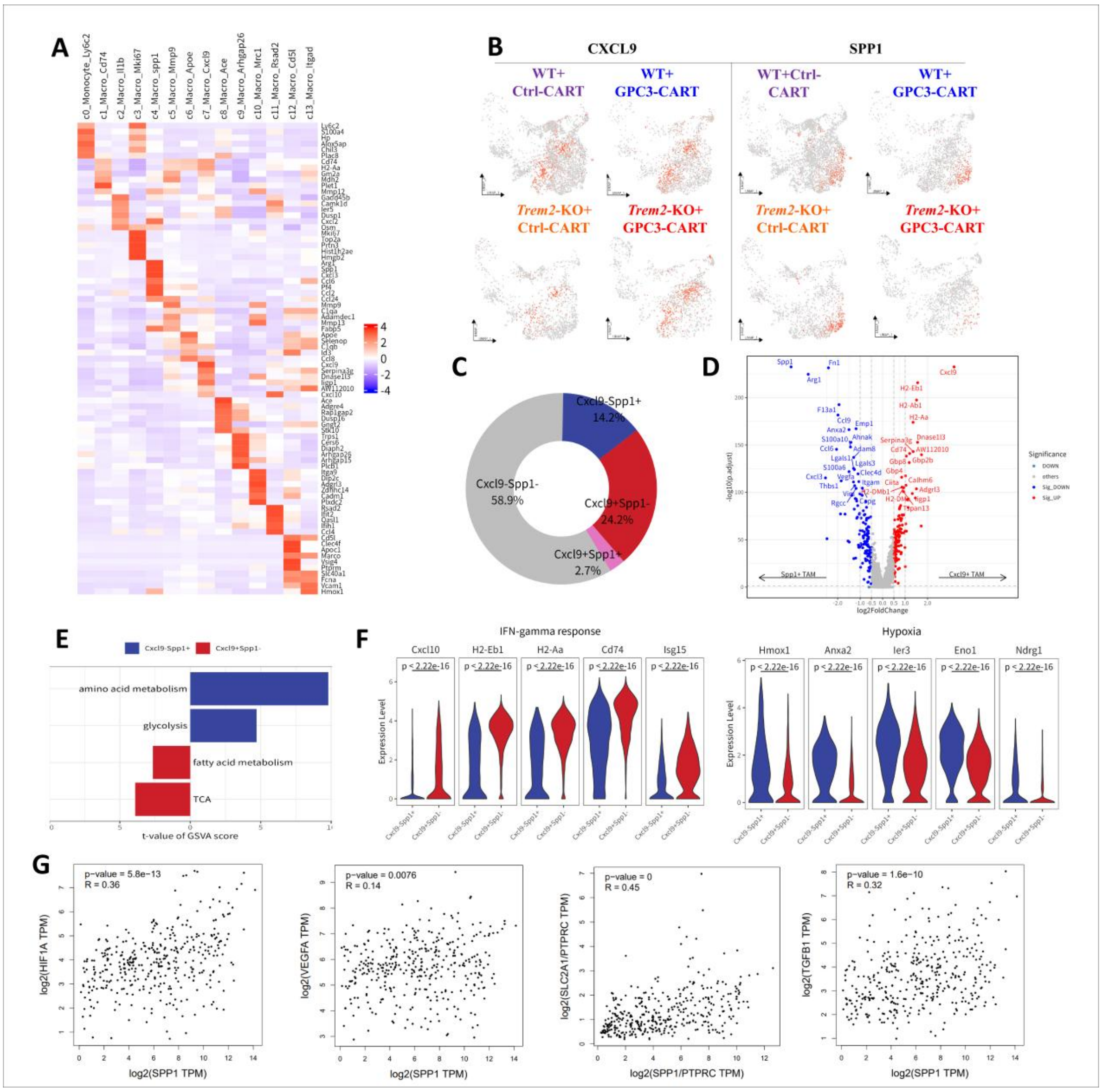

Figure S3. Different functions between *Cxcl9*$^{+}$ and *Spp1*$^{+}$ macrophages, related to Figure 4 and Figure 5.

(A) Heatmap of normalized expression level of top cluster markers in each monocyte or macrophage clusters.

(B) Normalised expression of *Cxcl9* and *Spp1* in TAMs among different groups.

(C) The cell proportion of *Cxcl9*$^{+}$*Spp1*$^{+}$, *Cxcl9*$^{+}$*Spp1*$^{-}$, *Cxcl9*$^{-}$*Spp1*$^{+}$ and *Cxcl9*$^{-}$*Spp1*$^{-}$ macrophages.

(D) Volcano plot shows the different expressed genes between *Cxcl9*$^{+}$*Spp1*$^{-}$ and *Cxcl9*$^{-}$*Spp1*$^{+}$ macrophages.

(E) Bar plot shows differential enriched metabolism pathways between *Cxcl9*$^{+}$*Spp1*$^{-}$ and *Cxcl9*$^{-}$*Spp1*$^{+}$ macrophages by Gene set variance analysis (GSVA). *Cxcl9*$^{+}$*Spp1*$^{-}$ macrophages exhibited increased levels in TCA cycle and fatty acid metabolism, while *Cxcl9*$^{-}$*Spp1*$^{+}$ macrophages increased in glycolysis

and amino acid metabolism.

(F) Violin plot shows the different expressed genes between *Cxcl9*$^+$*Spp1*$^-$ and *Cxcl9*$^-$*Spp1*$^+$ macrophages in multiple pathways including IFN-gamma response and hypoxia.

(G) Scatter plot shows the validation of the correlation between some genes related to pathways in E-G and *Spp1*$^+$ macrophages in TCGA-LIHC. TPM data was used as the expression level of each gene, and normalized to immune cells by PTPRC expression.

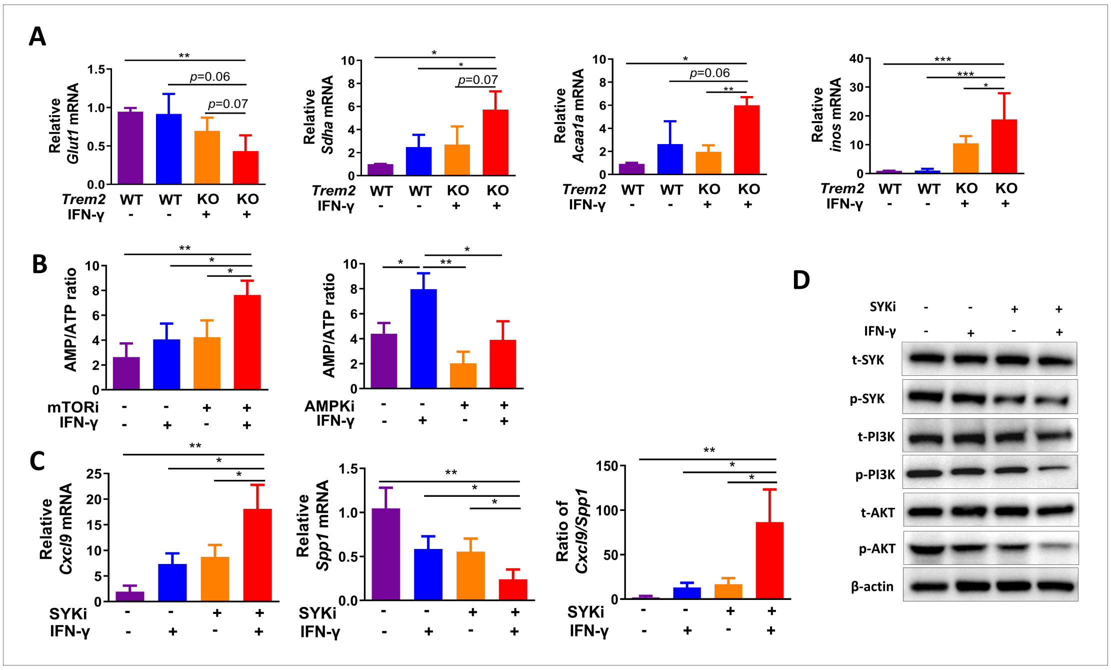

Figure S4. *Trem2*-KO regulates metabolism by activating the AMPK signal pathway, related to Figure 5.

(A) Relative mRNA expression levels of glycolysis, TCA and fatty acid oxidation marker genes (including *Ldha*, *Glut1*, *Sdha*, *Idh2*, Cpt1, and Acaa1a) in BMDMs were quantified by RT-qPCR. N=3, Wilcoxon tests, *p <0.05, **p <0.01, ***p <0.001.

(B) AMP and ATP concentrations in BMDMs were measured using dedicated assay kits, and the AMP/ATP ratio was calculated from these values. N=3. Wilcoxon tests, *p <0.05, **p <0.01, ***p <0.001.

(C) Relative mRNA expression levels of *Cxcl9, Spp1* in BMDMs were quantified by RT-qPCR, and the ratio of *Cxcl9/Spp1* was calculated. N=3. Wilcoxon tests, *p <0.05, **p <0.01, ***p <0.001.

(D) BMDMs isolated from WT mice were educated for 24 hours in a transwell system with cancer cells, with or without IFN-γ (40 ng/mL). BMDMs were pre-treated with SYK inhibitor (R406, 1μM)

for 2 hours prior to co-culture. Relative protein expression levels of indicated molecular were assessed by WB and normalized to β-actin.

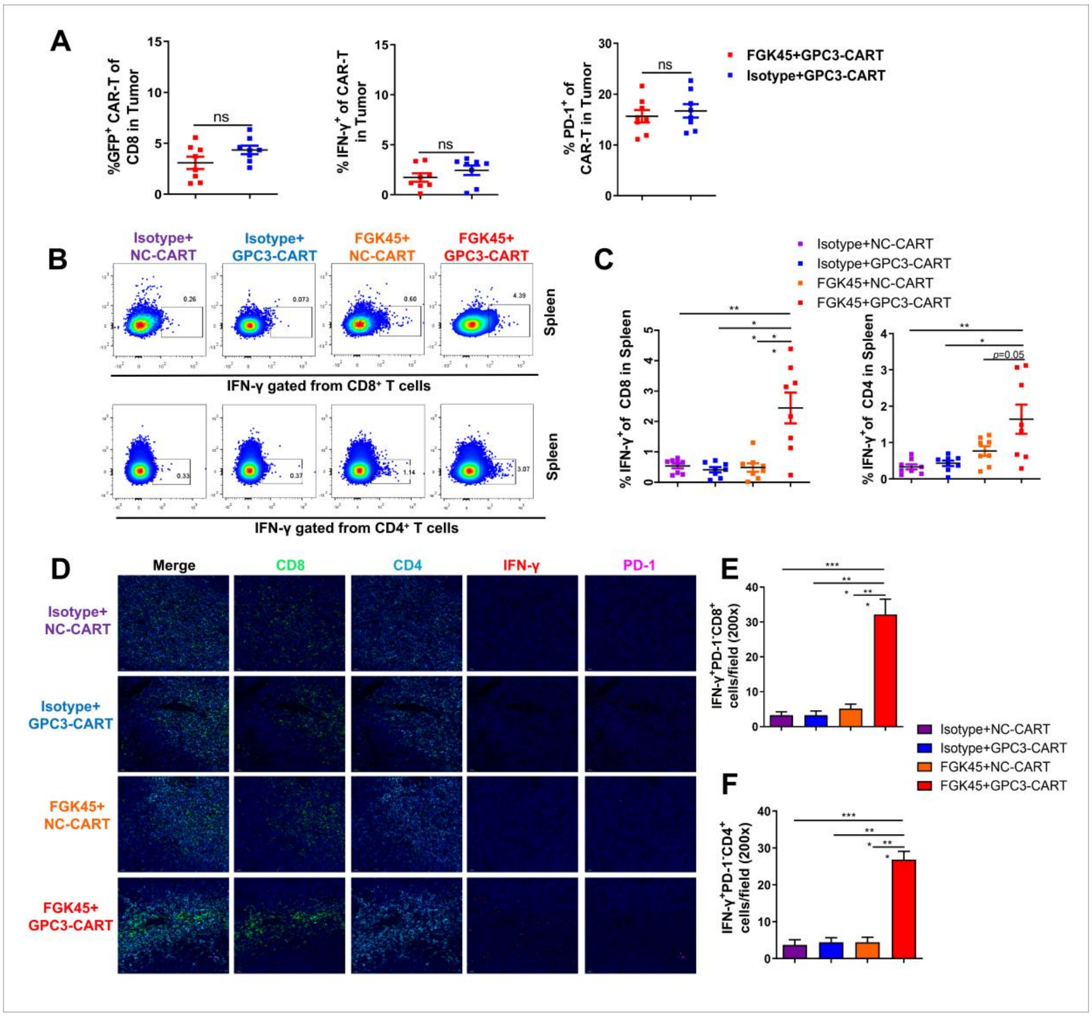

Figure S5. PD-1⁻IFN-γ⁺ CD8⁺/CD4⁺ T cells were increased in the splenic microenvironment of FGK45+GPC3-CAR-T group, related to Figure 7.

(A) Quantification of effector/exhausted CART cells from TILs, detecting by Flow cytometry. N = 8.

(B-C) Flow cytometry and quantification plots of IFN-γ^+CD8$^+$ T cells (effector CD8) and IFN-γ^+CD4$^+$ T cells (effector Th1), as well as IFN-γ^-CD8$^+$ T cells (exhausted CD8) and IFN-γ^-CD4$^+$ T cells (exhausted Th1) from spleen *in vivo*. N = 8.

(D-F) .Multiple immunofluorescence images of CD8$^+$ and CD4$^+$ T cells in spleen tissue between groups. Scale bar=50μm. Statistical plot representing the relative number of T cell subsets. (Quantification) Data are presented as mean ± SEM. The quantification of cell numbers was performed

by analyzing a minimum of three random low-power fields per condition per experiment. n = 3, *p <0.05, **p <0.01, ***p <0.001.

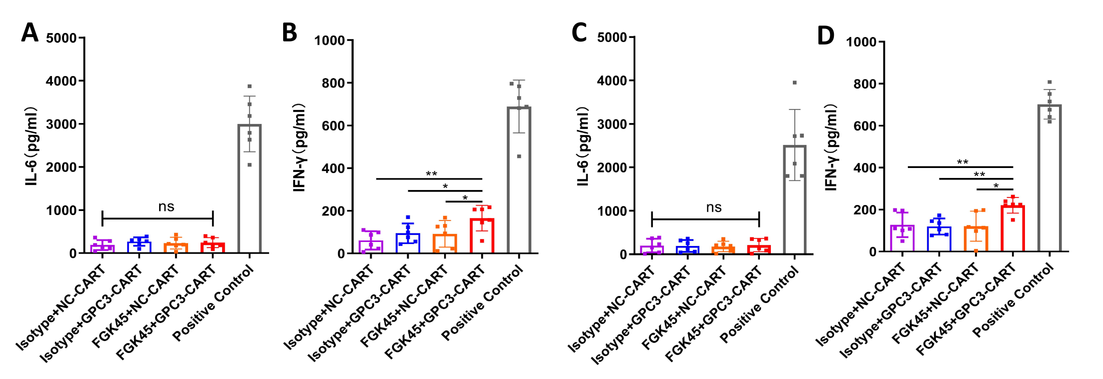

Figure S6. Safety Study of CD40 Agonist Combined with CAR-T Cell Therapy, related to Figure 7.

(A) Serum levels of mouse cytokines at 24 h following CAR T cell transfer (n= 6). A two-tailed unpaired two-sample t-test was used for statistical analysis. Positive control group for CRS. The murine lymphoma cell lines were transduced with recombinant retroviruses carrying human CD19. EL4 cells was used for C57 mice, and A20 cells was used for BALB/c mice. 3×10^6 cells was intraperitoneally injected into mice. Two weeks later, murine-derived αhCD19-CAR-T cells (3×10^7 cells) were adoptively transferred. After 24 hours, blood was collected and serum levels of mIL-6 and mIFN-γ were measured by ELISA.

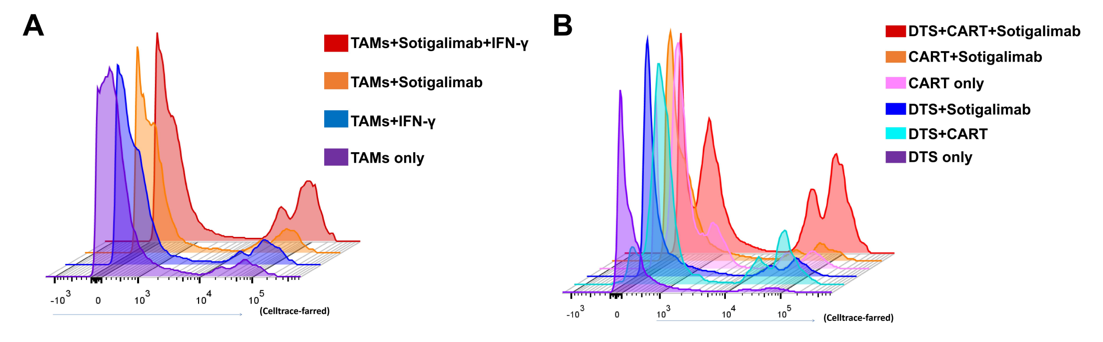

Figure S7. Detection of migrated CD8$^+$ T cells, related to Figure 7.

(A-B) Flow cytometry quantification of migrated CD8$^+$ T cells in different groups. (CellTrace-Far Red-labeled, n=5).

Table S1. Sequences of qRT-PCR Primers

Number	Gene	Primers
1	*Cxcl9*-5F	CCAGCCGAGGCACGATCC
2	*Cxcl9*-3R	ATCTCCGTTCTTCAGTGTAGCAATG
3	*Spp1*-5F	CCATCTCAGAAGCAGAATCTCCTTG
4	*Spp1*-3R	CTCCATCGTCATCATCATCGTCATC
5	*Ldha*-5F	GTGTGGAGTGGTGTGAATGTTGC
6	*Ldha*-3R	CTCCTTCCACTGCTCCTTGTCTG
7	*Idh2*-5F	GGATCATCTGGCAGTTCATCAAGG
8	*Idh2*-3R	CATTGGTCTGGTCACGGTTTGG
9	*Cpt1*-5F	ACGGCAGAGCAGAGGTTCAAG
10	*Cpt1*-3R	ACACCACATAGAGGCAGAAGAGG
11	*Glut1*-5F	TGAAAGAAGAGGGTCGGCAGATG
12	*Glut1*-3R	AGCACCACAGCGATGAGGATG
13	Sdha-5F	GGACATCAAGACTGGCAAGGTTAC
14	Sdha-3R	CAGTAGGAGCGGATAGCAGGAG
15	*Acaa1a*-5F	CCGCCTGCTGGAGAGTGAG
16	*Acaa1a*-3R	TCTGCCGTGAAATGCCAAACC
17	*Inos*-5F	TCACTCAGCCAAGCCCTCAC
18	*Inos*-3R	TCCAATCTCTGCCTATCCGTCTC

Data S1. Unedited gel images

Full unedited gel for Figure 5F

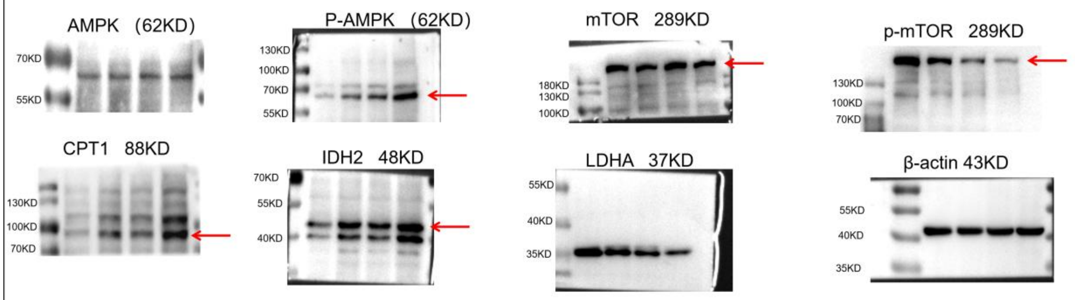

Full unedited gel for Figure 5G

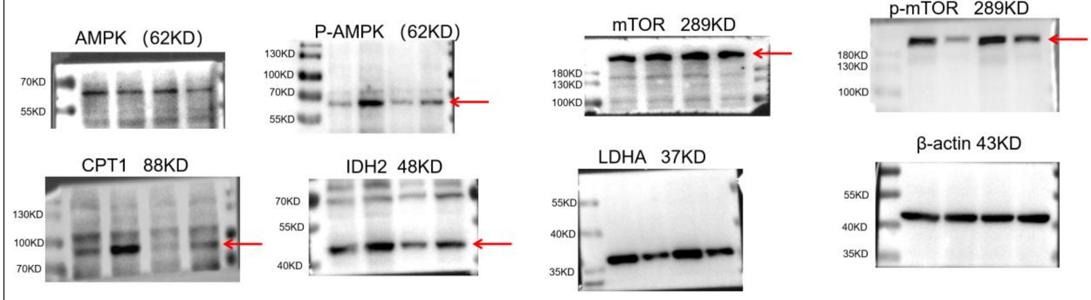

Full unedited gel for Figure 5H

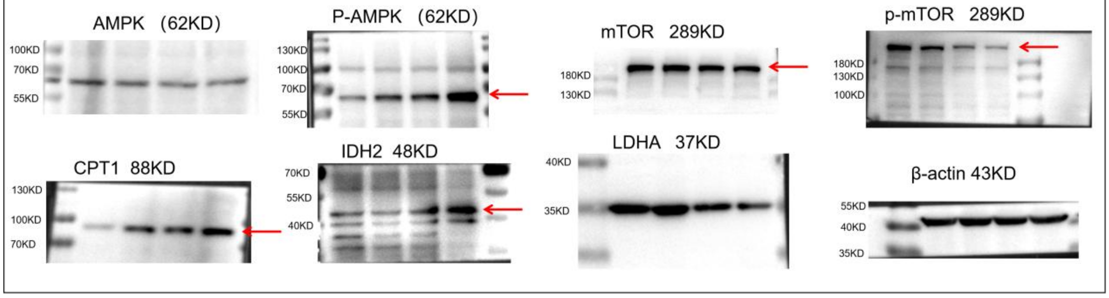

Full unedited gel for Figure 5M

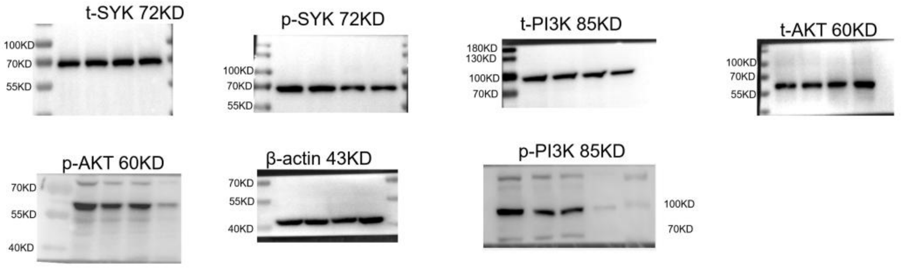

Full unedited gel for supplemental Figure S4D

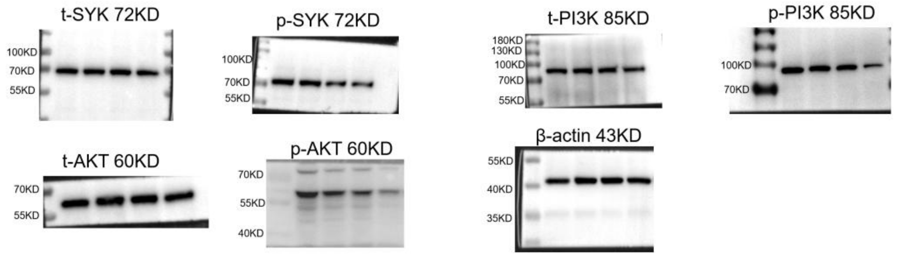

Full unedited gel for Figure 5N

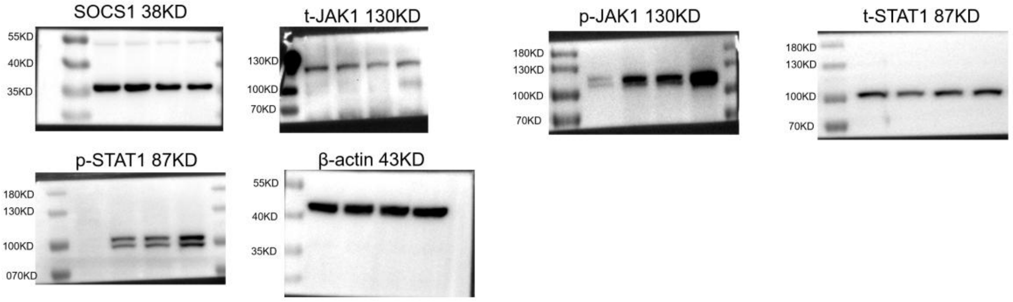

Full unedited gel for Figure 5O

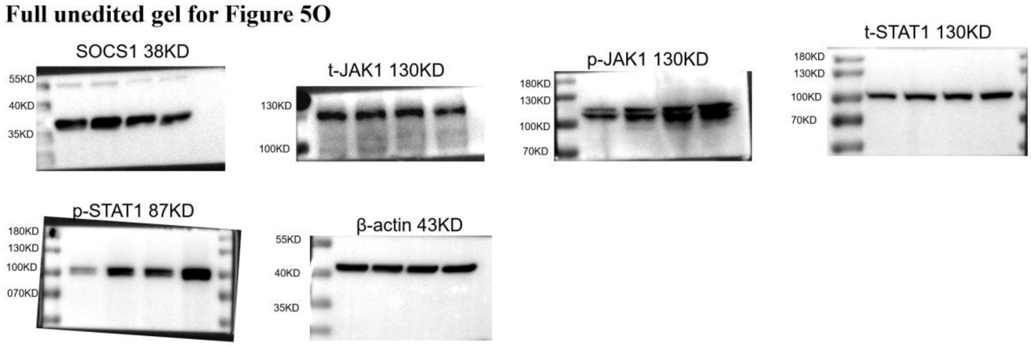

Full unedited gel for Figure 5P

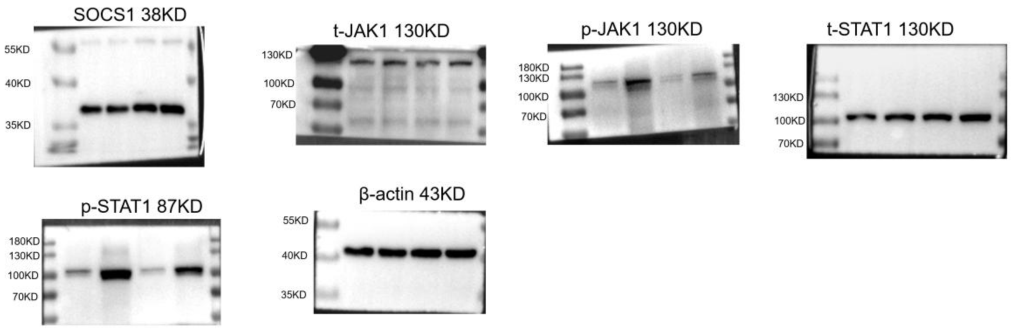

Full unedited gel for Figure 6K

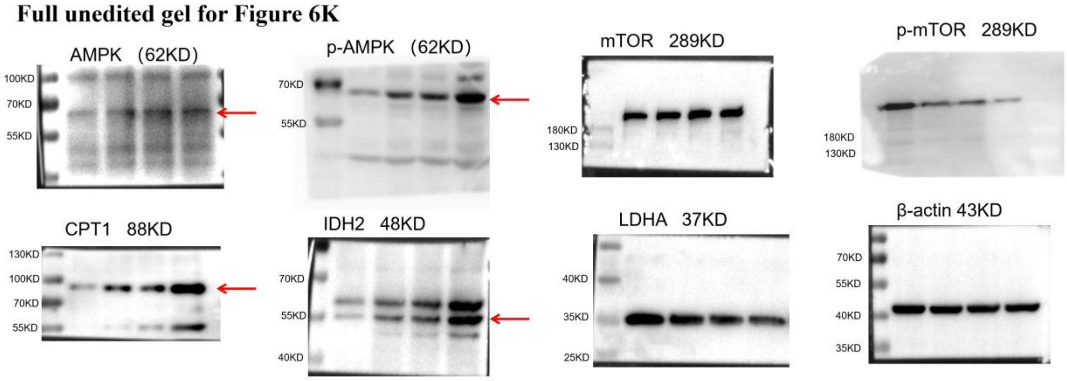

Full unedited gel for Figure 6L

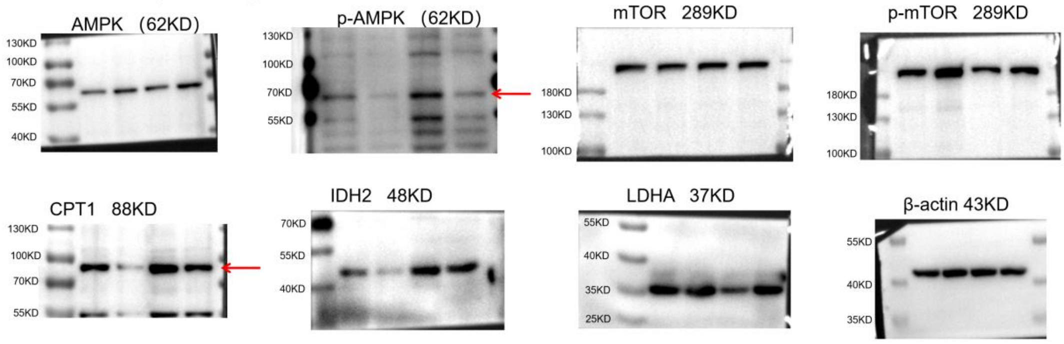

Full unedited gel for Figure 6D

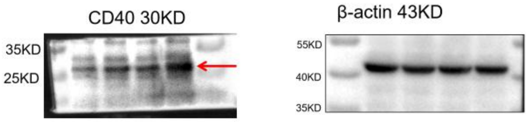

Full unedited gel for Figure 6O

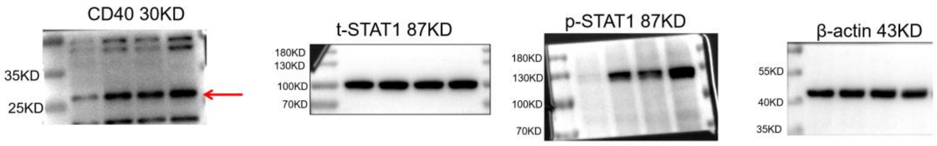

Full unedited gel for Figure 6P

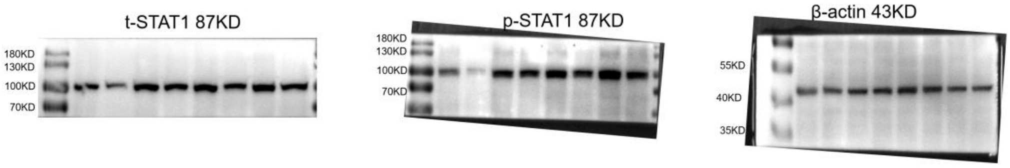

Supplement: Document S2. Article plus supplemental information [file mmc2.pdf]
